# Astrocytic monoamine oxidase B (MAOB)–gamma-aminobutyric acid (GABA) axis as a molecular brake on repair following spinal cord injury

**DOI:** 10.1038/s41392-025-02398-2

**Published:** 2025-09-11

**Authors:** Hye Yeong Lee, Jung Moo Lee, Hye-Lan Lee, Jiyeon Park, Heeyoung An, Eun Kyung Park, Sae Yeon Hwang, Sol lip Yoon, Gwang Yong Hwang, Keung Nyun Kim, Min-Ho Nam, Seung Eun Lee, Hyunji Kang, Joungha Won, Bo Ko Jang, Elijah Hwejin Lee, SunYeong Choi, Mingu Gordon Park, Sang Wook Kim, Ki Duk Park, SeungHwan Lee, C. Justin Lee, Yoon Ha

**Affiliations:** 1 Spine & Spinal Cord Institute, Department of Neurosurgery, College of Medicine, Yonsei University, Seoul, Republic of Korea; 2 Center for Cognition and Sociality, Institute for Basic Science, Daejeon, Republic of Korea; 3 Department of Clinical Pharmacology and Therapeutics, Seoul National University College of Medicine and Hospital, Seoul, Republic of Korea; 4 NeuroBiogen Co., LTD, Seocho-gu, Seoul, Republic of Korea; 5 Brain Science Institute, Korea Institute of Science and Technology (KIST), Seoul, Republic of Korea; 6 University of Science and Technology, Seoul, Republic of Korea; 7 Research Animal Resources Center, Korea Institute of Science and Technology (KIST), Seoul, Korea; 8 Department of Biomedical Engineering, Ulsan National Institute of Science and Technology (UNIST), Ulsan, Republic of Korea; 9 POSTECH Biotech Center, Pohang University of Science and Technology (POSTECH), Pohang, Republic of Korea

**Keywords:** Regeneration and repair in the nervous system, Diseases of the nervous system, Molecular neuroscience, Cellular neuroscience

## Abstract

Neuroregeneration and remyelination rarely occur in the adult mammalian brain and spinal cord following central nervous system (CNS) injury. The glial scar has been proposed as a major contributor to this failure in the regenerative process. However, its underlying molecular and cellular mechanisms remain unclear. Here, we report that monoamine oxidase B (MAOB)-dependent excessive γ-aminobutyric acid (GABA) release from reactive astrocytes suppresses the CNS repair system by reducing brain‒derived neurotrophic factor (BDNF) and tropomyosin receptor kinase B (TrkB) expression in severe spinal cord injury (SCI) animal models. Genetic deletion of MAOB in a mouse SCI model promotes both functional and tissue recovery. Notably, the selective MAOB inhibitor, KDS2010, facilitates recovery and regeneration by disinhibiting the BDNF-TrkB axis in a rat SCI model. Its dose-dependent effects were further validated in a monkey SCI model. Moreover, KDS2010 demonstrated a tolerable safety profile and dose-proportional pharmacokinetics in healthy humans during a phase 1 clinical trial. This pathway therefore represents a pivotal target for overcoming the intrinsic barriers to CNS repair after injury. Our findings identify the astrocytic MAOB‒GABA axis as a crucial molecular and cellular brake on the CNS repair system following SCI and highlight the translational potential of KDS2010 as a promising therapeutic candidate for SCI treatment.

## Introduction

Spinal cord injury (SCI) is a devastating neurological condition that can result from traumatic events such as motor vehicle accidents, falls, sports injuries, or acts of violence, and often leads to partial or complete loss of motor and sensory function below the site of injury. Beyond the profound physical disability, SCI imposes a substantial psychological, social, and economic burden on patients, families, and healthcare systems worldwide. It is firmly believed that once neurons die due to severe brain or SCI, regeneration of neurons rarely occurs in the adult mammalian central nervous system (CNS).^1,2^ This failure of the mammalian CNS repair system has been attributed to the notorious glial scar, which fills in and surrounds the injured site after severe damage to the CNS.^3–5^ Despite lines of evidence, how glial scars establish a brake on the CNS repair system has remained a mystery for several decades.

The role of the glial scar in injured mammalian tissue is complicated.^6^ Some studies reported a neuroprotective role of reactive astrocytes in mild acute injury conditions,^4,7^ where these cells can limit the spread of inflammatory cells, into adjacent intact parenchyma, secrete trophic factors such as brain-derived neurotrophic factor (BDNF) and ciliary neurotrophic factor (CNTF) to support neuronal survival, regulate extracellular ion homeostasis, and form a temporary physical and biochemical barrier that shields unaffected neural tissue from secondary damage. In contrast, numerous studies have suggested that in more severe or chronic injury settings, reactive astrocytes inhibit neuroregeneration and tissue recovery by releasing inhibitory molecules such as pro-inflammatory cytokines (e.g., interleukin-1 beta; IL-1β, tumor necrosis factor-alpha; TNF-α) and extracellular matrix components like chondroitin sulfate proteoglycan (CSPG), which alter the extracellular milieu and are proposed to serve as a non-permissive barrier to CNS axon extension.^8–10^ Although there appear to be sufficient lines of circumstantial evidence supporting the idea that scar-forming reactive astrocytes play a detrimental role in neuroregeneration and recovery, the debate continues. This ongoing debate is probably due to both the lack of identification of a precise molecular pathway that directly mediates this inhibitory effect, including signaling cascades within reactive astrocytes and their downstream targets on neurons and oligodendrocytes, and the limited availability of molecular and pharmacological tools capable of selectively modulating scar-forming astrocytes without disrupting their acute protective functions.

We have recently developed powerful molecular and pharmacological tools to abrogate reactive astrogliosis and glial scar in various neuroinflammatory conditions.^11–16^ These tools can effectively block γ-aminobutyric acid (GABA) and H_2_O_2_-production by targeting either the putrescine degradation pathway via monoamine oxidase B (MAOB) or the putrescine synthesis pathway via the urea cycle. Notably, the reversible and selective MAOB inhibitor, KDS2010, has been shown to reverse learning and memory impairment in Alzheimer’s disease mouse model,^11,12^ motor impairment in Parkinson’s disease mouse model,^14^ and improve motor performance in a white matter stroke rat model.^13^ In each of these disease contexts, pharmacological inhibition of MAOB by KDS2010 led to a marked suppression of excessive astrocytic GABA synthesis, accompanied by a restoration of excitatory synaptic transmission and enhanced neuronal circuit activity, as measured by both behavioral and electrophysiological endpoints. These findings collectively highlight the inhibitory roles of MAOB-dependent astrocytic GABA production via the putrescine degradation pathway in multiple pathological conditions, spanning both gray and white matter injury. However, despite these converging lines of evidence, the potential inhibitory role of astrocytic GABA in recovery after SCI has not been systematically investigated, and its contribution to post-injury neuroregeneration remains unexplored.

This study revealed that MAOB-dependent excessive GABA from scar-forming reactive astrocytes acts as a molecular and cellular brake on recovery after SCI by downregulating the expression of key neuroregenerative mediators, specifically BDNF and its high-affinity receptor, tropomyosin receptor kinase B (TrkB), within and around the lesion site. Disruption of the BDNF‒TrkB axis through genetic or pharmacological inhibition of the MAOB‒GABA axis not only restores BDNF and TrkB levels but also enhances axonal growth, synaptic connectivity, and functional motor recovery in severe SCI animal models, ranging from rodent to nonhuman primate. Additionally, findings from the phase 1 clinical trial of the KDS2010 conducted in healthy human volunteers demonstrate its safety and tolerability across multiple ascending dose cohorts, with dose-proportional pharmacokinetics and no serious adverse events reported. The integration of preclinical and clinical data provides strong support for the therapeutic potential of MAOB inhibition in SCI recovery, bridging mechanistic insights with translational application and offering a viable pathway toward clinical intervention.

## Results

### Astrocytic MAOB impedes functional and tissue recovery in the mouse SCI model

**Fig. 1:**
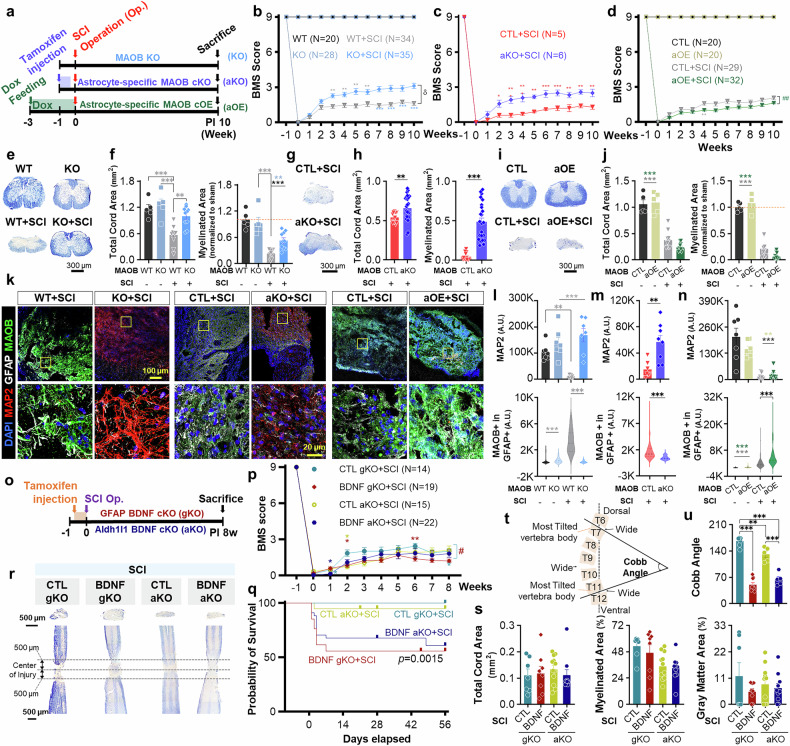
Astrocytic MAOB and GABA impede functional and tissue recovery, whereas astrocytic BDNF is critical for survival after SCI. **a** Experimental timelines using MAOB KO, aKO, and aOE. **b** BMS score of each group (MAOB WT, WT + SCI, MAOB KO, and KO + SCI) for a total of 11 weeks (1 week before and 10 weeks after the surgical operation). **c**, **d** BMS score of each group for aKO (**c**), and aOE (**d**) for a total of 11 weeks. **e**, **g**, **i** EC staining of spinal cord tissues from MAOB KO (**e**), aKO (**g**), and aOE (**i**) at PI 10w. **f**, **h**, **j** Total cord area (left) and myelinated area (right) of MAOB KO (**f**), aKO (**h**), and aOE (**j**) mice in each group, as determined by EC staining. The myelinated area was normalized to that of the WT in group the MAOB KO and aOE. **k** Confocal images of the injured areas of MAOB KO (left), aKO (middle), and aOE (right) stained with anti-MAP2 (red), anti-GFAP (white), and anti-MAOB (green) antibodies, and DAPI (blue) at PI 10w. Each yellow box in the merged images indicates the magnified region of interest. **l**–**n** Mean intensity of the MAP2 (top) and GFAP-positive MAOB (bottom) in each group for MAOB KO (**l**), aKO (**m**), aOE (**n**) at PI 10w. **o** Experimental timeline using BDNF gKO and aKO. **p** BMS score of each group for a total of 9 weeks. **q** Survival curves of each group. **r** EC staining of cross (top) and longitudinal (bottom) sections of spinal cord tissues in each group (CTL 80 gKO+SCI, BDNF gKO+SCI, CTL aKO+SCI, and BDNF aKO+SCI) at PI 8w. **s** Total cord, myelinated, 81 and gray matter areas did not show any difference among the groups. **t** Schematic figure showing the calculation method for the Cobb angle. **u** Calculated the Cobb angle of each group at PI 8w (left) and representative image showing spine deformity of BDNF gKO+SCI (right). All data are expressed as the means ± S.E.M.s *P < 0.05; **P < 0.01; ***P < 0.001; n.s., not significant. #, ##, ###, and δ indicate *P* < 0.05, *P* < 0.01, *P* < 0.001, and *P* < 0.0001, respectively, for the linear mixed model

To assess the pathological role of MAOB in SCI, we examined the recovery after SCI via three types of genetically modified *Maob* mouse line: MAOB knockout (KO), astrocyte-specific MAOB conditional knockout (aKO), and astrocyte-specific MAOB conditional overexpression (aOE) strains. Severe crush injury was carried out at thoracic vertebra number 10 in each mouse line. After generating the severe SCI model, we assessed the Basso Mouse Scale (BMS) for locomotion score^17^ to test functional recovery once a week for a total of 11 weeks (1 week before and 10 weeks after the surgical operation) (Fig. 1a). Compared with the WT with SCI group (WT + SCI), the MAOB-KO with SCI group (MAOB-KO + SCI) exhibited significant recovery of motor function beginning at 3 w post-injury (PI), whereas the sham-operated WT and MAOB-KO groups presented no signs of motor dysfunction (Fig. 1b). Notably, at PI 10w, MAOB KO + SCI achieved a score of 3.11 ± 0.22, whereas the WT + SCI group had a score of only 1.62 ± 0.16 (Fig. 1b). According to the BMS scoring criteria,^17^ a score of 1 ~ 2 is defined as slight or extensive ankle movement and a score of 3 indicates the threshold at which the animal can support its own body weight.^17,18^ Therefore, the difference between the scores of 1~2 and 3 in a severe SCI mouse model indicates substantial functional recovery. Similarly, compared with the CTL + SCI group, the aKO+SCI group also exhibited significant functional recovery of motor function at 2 w PI (Fig. 1c). At 10 weeks post-injury, the aKO+SCI group (15.58%) accounted for ~77% of the improvement in the BMS score observed in the MAOB KO + SCI group (20.16%) (Fig. 1b, c). Moreover, the combined total cord area and myelinated area of the aKO+SCI group (44.80%) accounted for ~81% of that of the KO + SCI group (55.15%) (Fig. 1e–g). These data suggest that astrocytic MAOB plays a dominant role in mediating the deleterious effects of SCI. In contrast, aOE+SCI exhibited more severe motor dysfunction than CTL + SCI did (Fig. 1d). These results indicate that MAOB, which is specifically expressed in astrocytes, hinders functional recovery after SCI.

We next examined the extent of injury at the tissue level via the Eriochrome Cyanine (EC) staining at PI 10w. Compared with WT and MAOB KO, WT + SCI presented significantly reduced total spinal cord, myelinated, and gray matter areas, which were all significantly restored in MAOB KO + SCI (Fig. 1e, f). Similarly, aKO+SCI presented significantly larger total spinal cord, myelinated, and gray matter areas than CTL + SCI did(Fig. 1g, h), whereas aOE+SCI presented a tendency toward smaller total spinal cord, myelinated, and gray matter areas than CTL + SCI did at PI 10w (Fig. 1i, j). These results indicate that astrocytic MAOB impedes functional and tissue recovery after SCI.

To test whether this functional/tissue recovery correlates with neuronal preservation, structural stabilization, and possible regeneration, and whether it is associated with changes in astrocyte reactivity, we performed immunohistochemistry with antibodies against microtubule-associated protein 2 (MAP2; a neuronal marker), glial fibrillary acidic protein (GFAP; an astrocytic marker), and MAOB (a reactive astrocytic marker)^11^ in the ventral region of the spinal cord. The intensity of MAP2 was significantly decreased in the WT + SCI, whereas it was fully recovered in MAOB KO + SCI (Fig. 1k, l and Supplementary Fig. 1a), indicating that neuronal preservation, structural stabilization, and possible regeneration positively correlate with functional and tissue recovery. On the other hand, the intensities of astrocytic GFAP and MAOB (94% reduction) in the WT + SCI were significantly greater than those in the WT, MAOB KO, or MAOB KO + SCI (Fig. 1k, l and Supplementary Fig. 1a), indicating a negative correlation between astrocyte reactivity and functional/tissue recovery. Additionally, we observed a significant increase in MAP2 intensity and a marked reduction in astrocytic GFAP and MAOB (70% reduction) in aKO+SCI compared with CTL + SCI (Fig. 1k, m and Supplementary Fig. 1b). In contrast, compared with the CTL + SCI group, the aOE+SCI showed no recovery in MAP2 intensity and a significant increase in astrocytic GFAP and MAOB (Fig. 1k, n and Supplementary Fig. 1c). These results further confirmed the positive correlation of neuronal preservation, structural stabilization, and possible regeneration with functional/tissue recovery, and the negative correlation of astrocyte reactivity with functional/tissue recovery.

### Astrocytic BDNF is critical for survival in mouse SCI model

To investigate the molecular and cellular identity of MAOB-dependent brakes in SCI, we considered the observed negative correlation between astrocyte reactivity and functional/tissue recovery after SCI, which is reminiscent of the proposed concept of a reciprocal relationship between MAOB-dependent GABA and the expression of the pro-form of brain-derived neurotrophic factor (proBDNF) in hypertrophic astrocytes.^19^ We previously defined two types of hypertrophic hippocampal astrocytes as either GABA-positive/proBDNF-negative ‘reactive astrocytes’ or proBDNF-positive/GABA-negative ‘active astrocytes’ in response to either aversive or beneficial environmental stimuli, respectively.^19^ This definition led us to investigate the roles of GABA and proBDNF in SCI.

This finding led us to hypothesize that astrocytic GABA might inhibit neuroregeneration by suppressing proBDNF expression after SCI. To test this hypothesis, we next examined the role of astrocytic BDNF in SCI via two types of astrocyte-specific BDNF cKO mice. These mice were generated by crossing GFAP-CreER^T2^ or Aldh1l1-Cre/ER^T2^ with *Bdnf* floxed mice and subsequently injecting tamoxifen for five consecutive days beginning at 7 weeks of age (BDNF gKO and BDNF aKO) (Fig. 1o). We observed that tamoxifen-treated mice (BDNF gKO+SCI and BDNF aKO+SCI) presented more severe motor dysfunction and lower survival rates than sunflower oil-treated control mice did (CTL gKO+SCI and CTL aKO+SCI) (Fig. 1p, q). Although total cord, myelinated, and gray matter areas did not differ (Fig. 1r, s), compared with sunflower oil-treated mice, tamoxifen-treated mice presented a more severe Cobb angle of the spine, indicating the extent of spine deformity (Fig. 1t, u). Interestingly, there were no significant differences in MAOB, GFAP, or astrocytic MAOB levels between the two groups (Supplementary Fig. 2a, b), suggesting that BDNF signaling is downstream of astrocyte reactivity. Moreover, tamoxifen-treated mice presented no change in GABA levels, accompanied by significantly lower proBDNF levels than sunflower oil-treated mice did (Supplementary Fig. 2c, d). These results provide novel mechanistic insights, positioning astrocytic GABA as an upstream regulator of BDNF signaling and astrocytic proBDNF as a critical contributor to survival after SCI. Together, these data support a mechanistic model in which elevated astrocytic GABA suppresses proBDNF expression, thereby impairing neuroregeneration following SCI.

### KDS2010, a selective MAOB inhibitor, promotes functional and tissue recovery in a rat SCI model

**Fig. 2:**
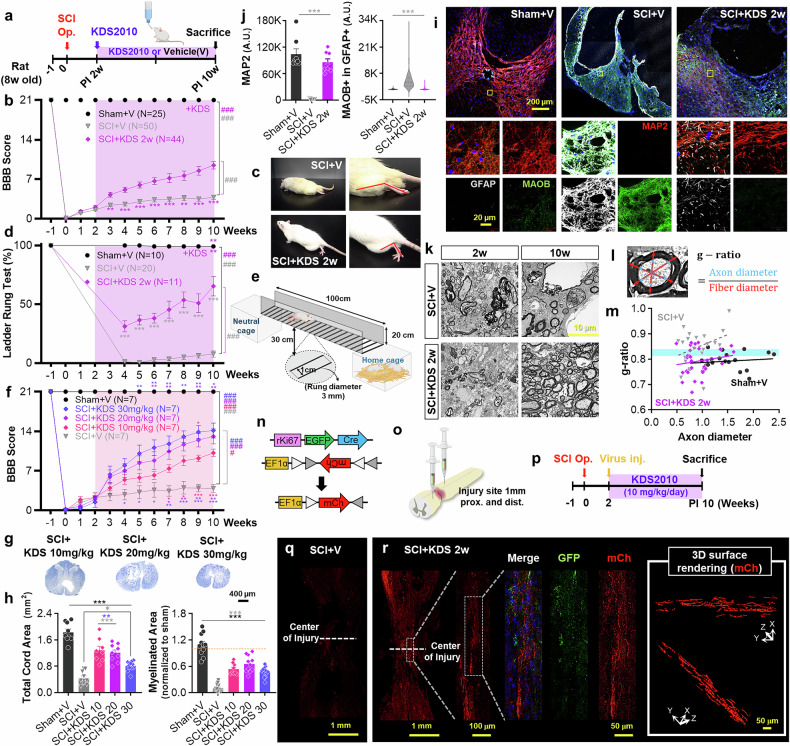
MAOB inhibition with KDS2010 causes functional/tissue recovery and neuroregeneration. **a** Experimental timelines for 8-week-old rats subjected to the SCI operation and treatment with the MAOB inhibitor, KDS2010, from 2 weeks after (subacute) SCI. **b** BBB score of each group (Sham+V, SCI + V, SCI + KDS 2w) for a total of 11 weeks (1 week before and 10 weeks after the surgical operation). **c** Hindlimb movement in the open field test for SCI + V and SCI + KDS 2w. The group labels indicate the treatment start point. **d** Percentage of hindlimb steps without slipping in each group with subacute phase KDS2010 treatment. **e** Schematic of the ladder rung test apparatus. Created with BioRender.com. **f** BBB score of each group with three different doses of KDS2010 (Sham+V, SCI + V, SCI + KDS 10 mpk, 20 mpk, and 30 mpk) for a total of 11 weeks. All doses of KDS2010 were administered from the subacute phase. **g** EC staining of spinal cord tissues at each dose at PI 10w. **h** Total cord area and myelinated area of each group via EC staining. **i** Confocal images of the injured area in each group stained with anti-MAP2 (red), anti-GFAP (white), and anti-MAOB (green) antibodies at PI 10w. Each yellow box in the merged images indicates the magnified region of interest. **j** Mean intensity of the MAP2 (left) and GFAP-positive MAOB (right) in each group. **k** TEM images of spinal cord tissues at PI 2w and 10w in SCI + V and SCI + KDS 2w. **l** Calculated method for g-ratio. **m** Calculated g-ratio in each group. The blue shading indicates the optimal range of g-ratio (0.790 ± 0.005). **n** Schematic figure showing DNA constructs in each virus and the result of recombination. **o** Schematic figure showing the virus injection sites at 1 mm proximal and distal the injured area. **p** Experimental timeline using rats with the SCI operation, virus injection, and subacute phase treatment with KDS2010 at 10 mpk. **q** Confocal images of the injured area in SCI + V stained with anti-GFP (green), mCh (red) antibodies and DAPI (blue) at PI 10w. **r** Confocal images of the injured area (left) and 3D surface rendering images of mCh signals in SCI + KDS 2w at PI 10w. Shaded areas in (**b**, **d**, and **k**) indicate the duration of KDS2010 administration. All the data are expressed as the means ± S.E.M.s *P < 0.05; **P < 0.01; ***P < 0.001; n.s., not significant. #, ##, and ### indicate *P* < 0.05, *P* < 0.01, *P* < 0.001, and *P* < 0.0001, respectively, for the linear mixed model

Next, we tested whether pharmacological inhibition of MAOB facilitates recovery after SCI. To test this hypothesis, we used the selective and reversible MAOB inhibitor KDS2010, which circumvents the shortcomings of irreversible MAOB inhibitors.^12^ We first evaluated the effect of KDS2010 on recovery after SCI at a dose of 10 mg/kg (mpk), which was previously reported to be effective in treating other neurological diseases.^12–14^ The pharmacokinetic (PK) analysis of KDS2010 was performed in mice, rats, and nonhuman primates. In rats, the area under the curve (AUC) at 9 mg/kg (9123.6 ± 4131.2 ng/h/mL) was sufficient for sustained CNS exposure and correlated well with therapeutic efficacy. In cynomolgus monkeys, a 10 mg/kg dose resulted in an AUC of 43,623.4 ± 1286.9 ng·h/mL with a half-life of 13.7 h. Increasing the dose to 30 mg/kg led to a disproportionate 5-fold increase in the AUC (~201,786.9 ng·h/mL) and a prolonged half-life (~31.1 h), suggesting a risk of off-target effects without corresponding efficacy gains (Supplementary Table 2). KDS2010 is highly permeable across the blood‒brain barrier.^12^ Since the blood‒spinal cord barrier (BSCB) is more permeable than the blood‒brain barrier^20^, KDS2010 is also expected to penetrate spinal cord tissue effectively. KDS2010 was administered via the drinking water ad libitum to both the sham-operated and SCI-operated groups, starting from the subacute phase (PI 2w) (Fig. 2a). We substituted the mouse SCI model with a rat SCI model, which has been reported to be better for pharmacological evaluation and closer to human SCI.^21^ We performed Basso, Beattie and Bresnahan (BBB) locomotor test^22^ to assess the functional recovery once a week for a total of 11 weeks (Fig. 2a). As a result, the SCI with KDS2010 treatment group (SCI + KDS 2w) showed significant motor recovery compared with the SCI with vehicle group (SCI + V) starting at PI 3w, reaching an average BBB score of 10.62 at PI 10w, enabling walking with weight on the sole (Fig. 2b, c and Supplementary Movie 1). In addition to the BBB locomotor test, we performed the ladder rung test by measuring the percentage of hindlimb steps without slipping as rats walked along a horizontal ladder^23^ (Fig. 2d, e). We observed significant motor recovery in SCI + KDS 2w, reaching approximately 65% of that in the sham-operated group, whereas the SCI + V showed no signs of recovery (Fig. 2d and Supplementary Movie 2). We then assessed the tissue recovery using EC staining on cross and longitudinal sections of the spinal cord. Compared to Sham+V and Sham+KDS, the SCI + V showed significantly less total spinal cord, myelinated, and gray matter, along with an enlarged cavity size (Supplementary Fig. 4a, b). These pathological changes were all significantly recovered in the SCI + KDS 2w group (Supplementary Fig. 4a, b, d). These results indicate that MAOB inhibition with KDS2010 facilitates functional/tissue recovery after SCI.

Next, we explored the dose-dependent effect of KDS2010 on recovery. The results revealed a dose-dependent improvement in the BBB locomotor scores with 10, 20, and 30 mpk at 10w PI (Fig. 2f), accompanied by significant tissue recovery (Fig. 2g, h). Furthermore, we evaluated the effects of KDS2010 at the subsaturating concentration of 10 mpk across different phases of SCI on recovery. Following MAOB inhibition from the acute phase (PI 1 d), the animals were divided into two groups: one with a significant recovery and the other with no recovery (Supplementary Fig. 7a, b). In the BBB locomotor test, we found that the SCI + KDS 1 d/Re achieved a stable gait with an average BBB score of 15.36 at PI 10w, whereas SCI+KDS1d/No did not show any behavioral recovery (Supplementary Fig. 7b), suggesting a potential association with the activation of alternative pathways, such as the DAO pathway, as previously reported.^24^ Indeed, the alternative diamine oxidase (DAO) pathway was activated in the SCI + KDS 1d/No (Supplementary Fig. 7l, m). At the tissue level, the SCI + V and SCI + KDS 1 d/No presented significantly reduced total spinal cord and myelinated areas, which were significantly recovered in the SCI + KDS 1 d/Re at PI 10w (Supplementary Fig. 7c, d). When we inhibited MAOB from the chronic phase (PI 6w), the SCI + KDS 6w also showed significant recovery in both the BBB locomotor test and the ladder rung test (Supplementary Fig. 8a–c), accompanied by a tendency toward larger total spinal cord and myelinated areas than the SCI + V (Supplementary Fig. 8d, e). However, the extent of recovery was less than that observed in the SCI + KDS 1 d/Re and the SCI + KDS 2w. Although the recovery group with acute-phase treatment (SCI + KDS 1 d/Re) showed better recovery than the group with subacute-phase treatment (SCI + KDS 2w), the acute-phase treatment appeared more variable because of the division into two groups with high recovery and no recovery. These results suggest that KDS2010 treatment from the subacute phase induces consistent and less variable functional and tissue recovery after SCI.

### KDS2010 induces neuronal preservation, structural stabilization, and remyelination in rat SCI model

Next, we examined neuronal preservation, structural stabilization, and the extent of astrocyte reactivity upon pharmacological inhibition of MAOB via KDS2010. Immunohistological analysis revealed that MAP2 intensity was significantly reduced in the SCI + V, whereas it was fully restored in the SCI + KDS 2w (Fig. 2i, j). The expression of astrocytic GFAP and MAOB was aberrantly increased in the SCI + V, whereas it returned to normal levels in the SCI + KDS 2w (Fig. 2i, j, and Supplementary Fig. 3). To further support these observations, we performed quantitative analysis of GFAP expression across all experimental groups (Supplementary Fig. 3). Consistent with the immunohistochemistry results, compared with SCI + V, KDS2010 treatment significantly reduced GFAP intensity, indicating effective attenuation of reactive astrogliosis. Moreover, immunostaining with antibodies against Tuj1 and GFAP in longitudinal sections produced consistent findings (Supplementary Fig. 5a). Similar results were observed with doses of 20 mpk and 30 mpk (Supplementary Fig. 6a, b) and with treatment from the acute (Supplementary Fig. 7e–i) or chronic phase (Supplementary Fig. 8f, g). These results again indicate a positive correlation of neuronal preservation, structural stabilization, and possible regeneration with functional/tissue recovery, and a negative correlation of astrocyte reactivity with functional/tissue recovery.

It has been previously reported that functional behavioral recovery is associated not only with neuroregeneration but also with remyelination.^25,26^ To assess the impact of MAOB inhibition on the extent of myelination after SCI, we performed transmission electron microscopy (TEM) at PI 2, 4, 6, and 10w. The TEM images revealed that the severe loss of myelination, which was observed throughout the PI 10w period in the SCI + V, gradually increased and fully recovered in SCI + KDS 2w at PI 10w (Fig. 2k and Supplementary Fig. 4e, f). The microstructures in the TEM images also povided evidence for remyelination (Supplementary Fig. 4c). Moreover, we observed that the g-ratio, a functional and structural index of axonal myelination or demyelination,^27^ in the SCI + KDS 2w (0.792 ± 0.010) was fully restored to the optimal range of 0.790 ± 0.005^27^ (Fig. 2l, m and Supplementary Fig. 4f). In addition, we confirmed a gradual increase in the number of myelinated axons and a gradual decrease in the density of cavities in the SCI + KDS 2w group via toluidine blue (TB) staining (Supplementary Fig. 4g, h). Finally, immunohistochemistry with antibodies against proliferation marker Ki67 and myelin basic protein (MBP; an oligodendrocyte marker) at PI 10w revealed a substantial population of oligodendrocytes positive for both Ki67 and MBP in the SCI + KDS 2w group (Supplementary Fig. 5d, e). Although neuronal nuclei (NeuN) and MBP are typically associated with postmitotic cells, the observed Ki67+NeuN+ and Ki67+MBP+ populations likely represent newly generated progenitors transiently expressing lineage markers during early differentiation after SCI.^28–36^ This interpretation is supported by Ki67-driven lineage tracing showing neuronal-like morphologies, which is consistent with injury-induced progenitor activation. These findings indicate that MAOB inhibition by KDS2010 facilitates oligodendrocyte proliferation and remyelination after SCI.

### KDS2010 facilitates neuronal proliferation and axon regeneration in rat SCI model

The increase in MAP2 and β-III tubulin (Tuj1) expression upon MAOB inhibition may be solely a result of a protective effect or invading axons from the peripheral nervous system rather than regenerating axons from proliferated neurons. Thus, we assessed the presence of proliferating neurons at the injury site via immunohistochemistry with antibodies against Ki67 and NeuN at PI 10w, as previous studies reported transient co-expression of Ki67 and NeuN during neurogenesis.^31,32^ As a result, we detected a significant increase in the number of neurons positive for both Ki67 and NeuN compared with those in the SCI + V group (Supplementary Fig. 5b, c). We then tested whether axon fibers from proliferating neurons can traverse the injury center upon MAOB inhibition. We employed a dual-viral strategy using a newly developed Ki67 promoter, which consists of a proliferation-dependent virus expressing Cre recombinase tagged with EGFP and a Cre-dependent virus expressing the fluorescent protein mCherry (mCh) (Fig. 2n). This strategy enables permanent labeling of both proliferating and proliferated cells with mCh from the time of virus injection (PI 2w) (Fig. 2o, p). This approach was validated in the dentate gyrus of rats, where the percentage of proliferating and proliferated cells was 5.14 ± 0.53% (*n* = 3) for 2 weeks, which aligns with the findings of a previous study^37^ (Supplementary Fig. 5f, g). In the SCI + KDS 2w group, we observed a small number of EGFP-positive signals and many mCh-positive signals, whereas the SCI + V presented almost no fluorescence signals (Fig. 2q, r). Notably, the mCh-positive signals in SCI + KDS 2w traversed the core of the injury site and can be predicted to be axon fibers via 3D rendering (Fig. 2r). These results indicate that pharmacological inhibition of MAOB using KDS2010 allows axon fibers from proliferating and proliferated neurons to traverse through the injury site. No such mCherry-positive signals were detected in sham-operated animals injected with the same viral constructs, confirming that axonal labeling is specific to injury-induced proliferative activity (Supplementary Fig. 6h–k).

### KDS2010 reduces astrocytic GABA levels and enhances proBDNF and TrkB expression in rat SCI model

We provide novel mechanistic insight by identifying astrocytic GABA as an upstream regulator of BDNF signaling (Fig. 1o–u). Additionally, numerous reports have demonstrated that BDNF‒TrkB signaling is critically involved in neuroregeneration and functional recovery after SCI.^38,39^ On the basis of this evidence, we further investigated whether MAOB inhibition with KDS2010 reduces GABA levels and enhances BDNF‒TrkB signaling, thereby promoting neuroregeneration and functional/tissue recovery in a severe rat SCI model. We first assessed the cellular content of GABA in GFAP-positive astrocytes via immunohistochemistry, following treatment with 10 mpk of KDS2010 from the subacute phase. The intensity of GABA, along with that of GFAP, significantly greater in the SCI + V compared to the Sham+V or the Sham+KDS, whereas it was significantly attenuated by MAOB inhibition in the SCI + KDS 2w (Supplementary Fig. 9a–c), even at different doses and phases of KDS2010 treatment (Supplementary Figs. 6c–f, 7l, m, 8h–k).

**Fig. 3:**
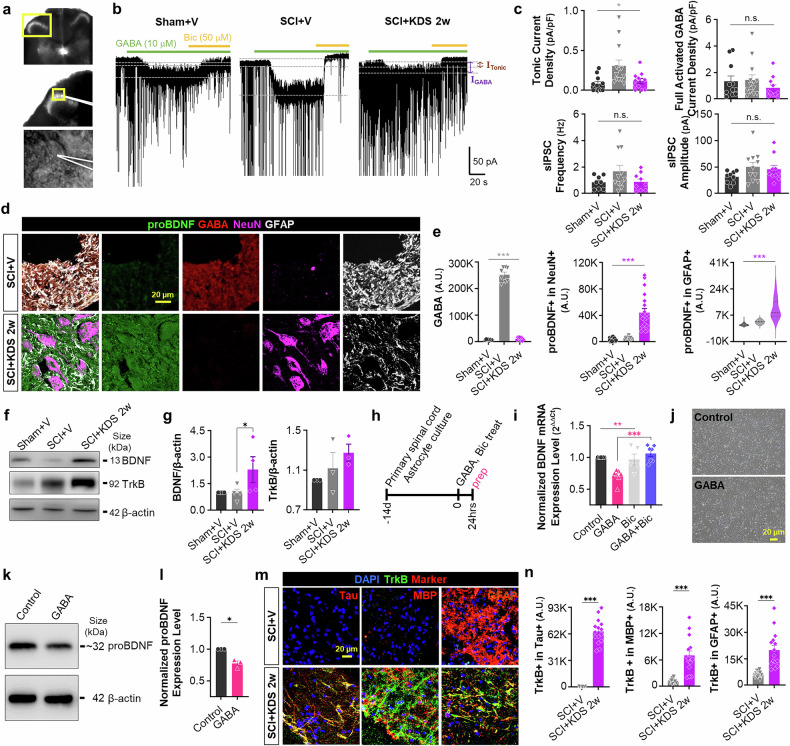
MAOB inhibition with KDS2010 reduces astrocytic GABA levels and enhances proBDNF and TrkB expression after SCI. **a** Differential interference contrast (DIC) images of the dorsal horn of the spinal cord (top and middle) and a magnified view of a whole-cell patch-clamped lamina II neuron (bottom). The yellow boxes indicate the magnified region of interest. **b** Representative traces of GABA_A_ receptor-mediated tonic GABA current in each group (Sham+V, SCI + V, and SCI + KDS 2w). The dashed lines (gray) and double-headed arrows (purple and brown) indicate baseline shifts (I_GABA_ and I_Tonic_) with bath application of GABA (10 μM, green bar) and bicuculline (Bic, 50 μM, orange bar). **c** Tonic GABA current density (top, left), GABA-induced full activation current density (top, right), frequency (bottom, left) and amplitude (bottom, right) of spontaneous inhibitory postsynaptic currents (sIPSCs) in each group. **d** Confocal images of the injured areas stained with anti-proBDNF (green), anti-GABA (red), anti-NeuN (magenta), and anti-GFAP (white) antibodies at PI 10w in SCI + V and SCI + KDS 2w. **e** Mean intensity of GABA (left), NeuN-positive proBDNF (middle), GFAP-positive proBDNF (right). **f** Western blotting of BDNF and TrkB in Sham+V, SCI + V, and SCI + KDS 2w at PI 10w. **g** Quantification of BDNF (left) and TrkB (right) expression levels in Western blotting. β-actin was used as a control for protein amount. **h** Experimental timeline for quantitative real-time PCR with GABA (100 μM) or Bic (50 μM) treatment of primary cultured spinal cord astrocytes at 14 days in vitro (DIV). **i** Relative (comparative Ct) BDNF expression level of each drug treatment condition (GABA, GABA+Bic, and Bic). **j** Representative images of control and GABA-treated spinal cord astrocytes. **k** Western blotting of proBDNF in control and GABA-treated condition. **l** Quantification of proBDNF in Western blotting. β-actin was used as a control for protein amount. **m** Confocal images of the injured areas stained with anti-TrkB (green), DAPI (blue), and each Tau, MBP, or GFAP (red) antibodies at PI 10w. KDS2010 treatment was initiated at PI 2w and continued daily until PI 10w. Group labels indicate the treatment start point. **n** Mean intensity of TrkB in the Tau-positive neurons (left), MBP-positive oligodendrocytes (middle), and GFAP-positive astrocytes (right) at PI 10w. All the data are expressed as the means ± S.E.M.s *P < 0.05; **P < 0.01; ***P < 0.001; n.s., not significant

To determine whether astrocytic GABA can be released and contribute to tonic GABA inhibition in neighboring neurons, we measured GABA_A_ receptor-mediated currents from lamina II dorsal horn neurons at PI 10w (Fig. 3a). We observed that tonic GABA current density was significantly greater in the SCI + V compared to Sham+V, whereas this aberrant tonic GABA current was significantly recovered in the SCI + KDS 2w group (Fig. 3b, c). We found no alterations in extrasynaptic GABA_A_ receptor expression or in the frequency and amplitude of spontaneous inhibitory postsynaptic currents (sIPSCs) (Fig. 3c). Furthermore, in the ELISA for GABA, we observed a significant reduction in GABA levels in the SCI + KDS 1 d/Re, whereas the SCI + V showed aberrant GABA levels at PI 10w (Supplementary Fig. 7j, k). These results indicate that the augmented GABA originates from the increased tonic release of GABA from reactive astrocytes in SCI.

We then investigated whether the proBDNF expression level could be altered by SCI or MAOB inhibition via immunohistochemistry. Remarkably, we found that the intensity of proBDNF in both astrocytes and neurons significantly increased in the SCI + KDS 2w group along with a significant decrease in GABA expression (Fig. 3d, e), even at different doses and phases of KDS2010 treatment (Supplementary Figs. 6e, f, 7l, m, 8h, i). This finding was confirmed by Western blotting (Fig. 3f, g). The appearance of proBDNF coincided with the disappearance of astrocytic GABA, reinforcing the idea of the reciprocal relationship between GABA and proBDNF. Next, we treated cultured primary spinal cord astrocytes with GABA for 24 h and found that GABA significantly downregulated the mRNA expression level of BDNF, which was fully restored by co-treatment with the GABA_A_ antagonist bicuculine (Fig. 3h, i). This GABA-induced reduction in proBDNF expression was also observed via Western blotting (Fig. 3j–l). These results support a novel mechanistic insight that astrocytic GABA acts as an upstream regulator of BDNF signaling.

We then assessed TrkB expression levels in Tau-positive neurons, MBP-positive oligodendrocytes, and GFAP-positive astrocytes. We observed that the intensity of TrkB significantly increased in neurons, oligodendrocytes, and astrocytes following MAOB inhibition from the subacute phase (Fig. 3m, n). Similar results were observed for TrkB in oligodendrocytes at different doses and phases of KDS2010 treatment (Supplementary Fig. 6g, h, Supplementary Fig. 8l, m). Given that proBDNF is proteolytically converted to mature brain-derived neurotrophic factor (mBDNF) by extracellular proteases such as tissue plasminogen activator,^40,41^ these results imply that MAOB inhibition causes the removal of astrocytic GABA and a coincidental induction of mBDNF-TrkB signaling pathway, which is indispensable for neuroregeneration and functional/tissue recovery after SCI.^38,39^

Additionally, we observed that KDS2010 treatment reduced the expression of CSPGs, such as neurocan and phosphacan, which are known to inhibit regeneration by creating a hostile extracellular environment (Supplementary Fig. 9d–h). These results further support the idea that MAOB inhibition promotes a more favorable environment for neuroregeneration.

### MAOB inhibition with KDS2010 facilitates tissue recovery in a monkey SCI model

**Fig. 4:**
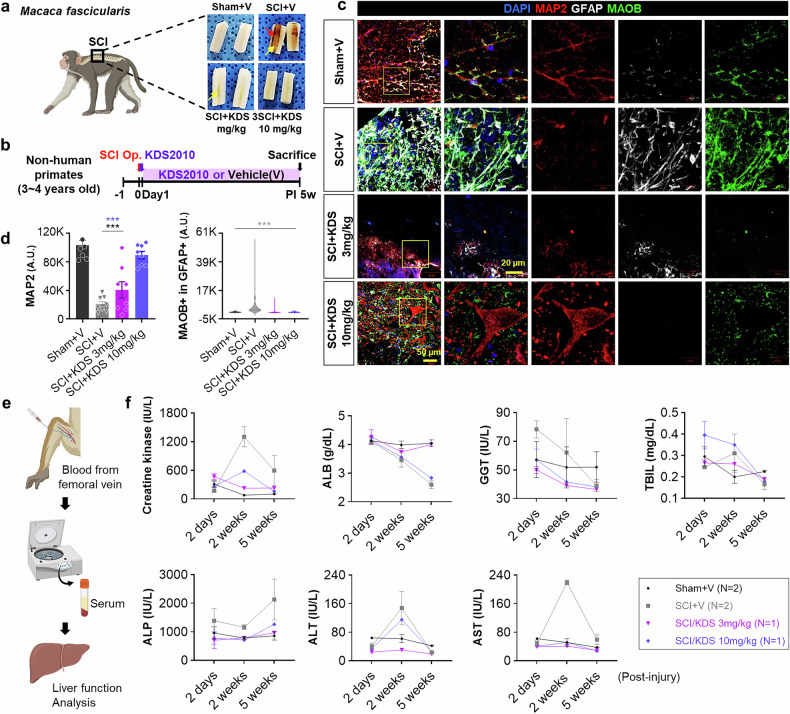
MAOB inhibition with KDS2010 facilitates tissue recovery after SCI in nonhuman primates. **a** Location of the SCI and representative longitudinal tissue sections at PI 5w in cynomolgus macaques (*Macaca fascicularis*). The red arrow indicates hematoma, and the yellow arrows indicate the cavities. Created with BioRender.com. **b** Experimental timelines using 3–4-year-old cynomolgus macaques subjected to the SCI operation and treatment with KDS2010 from 1 day after (acute) SCI. **c** Confocal images of the injured areas stained with anti-MAP2 (red), anti-GFAP (white), and anti-MAOB (green) antibodies, and DAPI (blue) at PI 5w in each group (Sham+V, SCI + V, SCI + KDS 3 mpk, and SCI + KDS 10 mpk). **d** Mean intensity of the MAP2 (left) and GFAP-positive MAOB (right) in each group. **e** Methodology illustration for blood chemistry analysis with nonhuman primate SCI model. Created with BioRender.com. **f** Blood chemistry and liver function data before and after KDS2010 treatment, showing the levels of serum creatine kinase (IU/L), albumin (ALB), gamma-glutamyl transferase (GGT, IU/L), total serum bilirubin (TBIL, mg/dL), alkaline phosphatase (ALP, IU/L), alanine transaminase (ALT, g/dL), and aspartate transaminase (AST, IU/L) at 2 days, 2 weeks, and 5 weeks post-injury. All the data are expressed as the means ± S.E.M.s *P < 0.05; **P < 0.01; ***P < 0.001; n.s., not significant

After confirming the efficacy of KDS2010 in promoting recovery from SCI in small animal models, we performed advanced tests in a nonhuman primate SCI model, followed by a phase 1 clinical trial. This phased approach enabled a thorough evaluation of both the safety and therapeutic potential of KDS2010. In the nonhuman primate study, a severe SCI model was generated in cynomolgus macaques (*Macaca fascicularis*), with KDS2010 administered at doses of 3 or 10 mpk from the acute phase of SCI (Fig. 4a, b). KDS2010 significantly reduced hematoma and cavity formation at the injury site, indicating a reduction in tissue damage (Fig. 4a). Immunohistochemical analysis revealed that MAP2 intensity was significantly greater in the SCI + KDS 10 mpk group than in the SCI + V group and tended to increase in intensity in the SCI + KDS 3 mpk group, compared to SCI + V (Fig. 4c, d). Moreover, astrocytic MAOB was significantly reduced in both KDS treatment groups, whereas increased astrocytic MAOB was observed in the SCI + V (Fig. 4c, d), confirming the ability of KDS2010 to promote neuronal preservation, structural stabilization and possible regeneration, as well as reduce the astrocyte reactivity.

However, in the blood chemistry and liver function analyses, the 10 mpk group presented mild signs of hepatic and muscle stress, evidenced by a moderate reduction in albumin (ALB) levels at PI 5w and transient increases in creatine kinase (CK) and alanine transaminase (ALT) levels at PI 2w, whereas the 3 mpk group showed no signs of stress (Fig. 4e, f). Although CK and ALT levels returned to normal at PI 5w in the 10 mpk group (Fig. 4e, f), these findings suggest that prolonged high-dose treatment may require closer monitoring for potential liver toxicity. Taken together, while the 10 mpk group showed better recovery than the 3 mpk group, 3 mpk is considered the No-Observed-Adverse-Effect Level (NOAEL) due to its safer profile.

### KDS2010 exhibits a tolerable safety profile and dose-proportional pharmacokinetics in humans

**Fig. 5:**
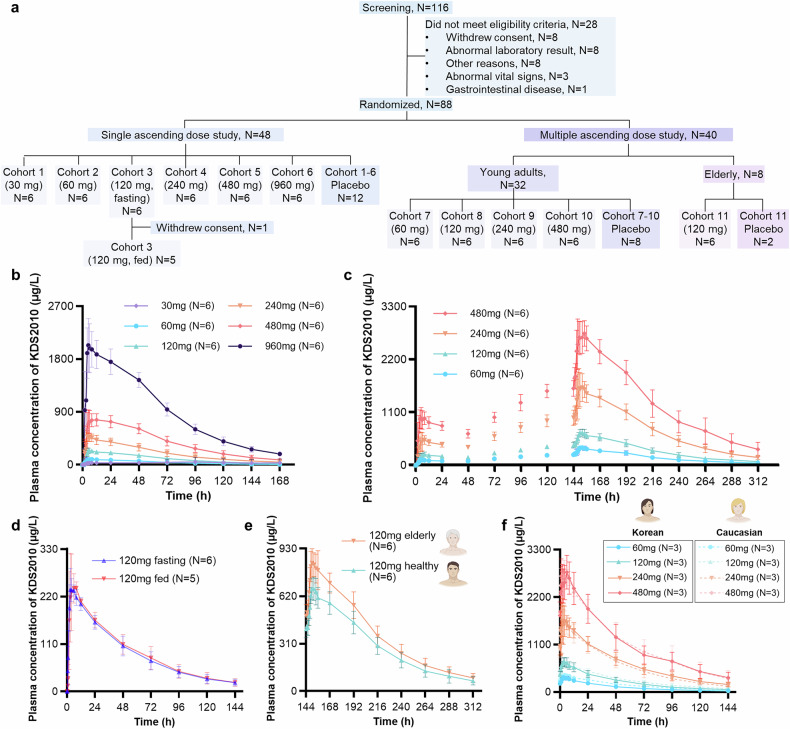
Pharmacokinetic profiles of KDS2010 in humans during the phase 1 clinical trial. **a** Subject disposition chart for the phase 1 clinical trial. **b**, **c** Plasma concentration-time profiles of KDS2010 in single ascending dose (**b**) and multiple ascending dose (**c**) studies. The dots and error bars indicate the mean concentration and standard deviation, respectively. **d** Plasma concentration-time profiles of KDS2010 following a single 120 mg administration under 10-h fasted conditions and after a high-fat meal. Dots and error bars indicate mean concentration and standard deviations, respectively. **e** Plasma concentration-time profiles of KDS2010 at steady state following 7-day administrations of 120 mg in elderly and young adults. Images are Created with BioRender.com. **f** Plasma concentration-time profiles of KDS2010 following administrations of 60, 120, 240, and 480 mg at steady state in Korean and Caucasian subjects. Images are Created with BioRender.com. All the data are expressed as the means ± S.E.M.s

A phase 1 clinical trial was conducted between September 2022 and May 2024 (Clinical Research Information Service registry no. KCT0008331). A total of 88 subjects were enrolled, with 87 completing the study. One subject withdrew due to a COVID-19 infection during the washout period of the food effect study (Fig. 5a). The 80 young healthy subjects had a mean age of 30.19 years; 77 were men and 3 were women; 41 were Korean and 39 were Caucasian. The 8 elderly male Korean subjects had a mean age of 74.00 years (Supplementary Table 3).

The safety profile of the KDS2010 was tolerable, with no serious adverse events observed. Treatment-emergent adverse events (TEAEs) were reported in 68 cases: 56 TEAEs in 25 subjects (37.9%) on the KDS2010 and 12 TEAEs in 6 subjects (27.3%) on placebo (Table 1). The majority of TEAEs are symptoms related to the autonomic nervous system, including somnolence, hypersomnia, dry eye, conjunctival hyperemia, and nasal congestion. The incidence of TEAEs tended to increase with dose, and a high-fat meal did not affect the frequency of TEAEs. Elderly subjects experienced gastrointestinal symptoms such as constipation, diarrhea, and abdominal discomfort. The incidence of TEAEs was similar between Korean and Caucasian subjects (data not shown).

**Table 1:** Incidence of treatment-emergent adverse events (TEAEs) in phase 1 clinical trial

	Single ascending dose study								Multiple ascending dose study						
	Young healthy adults								Young healthy adults					Healthy elderly	
	Cohort 1 (30 mg, *n* = 6)	Cohort 2 (60 mg, *n* = 6)	Cohort 3 (120 mg, fasting, *n* = 6)	Cohort 3 (120 mg, fed, *n* = 5)	Cohort 4 (240 mg, *n* = 6)	Cohort 5 (480 mg, *n* = 6)	Cohort 6 (960 mg, *n* = 6)	Placebo (*n* = 12)	Cohort 7 (60 mg, *n* = 6)	Cohort 8 (120 mg, *n* = 6)	Cohort 9 (240 mg, *n* = 6)	Cohort 10 (480 mg, *n* = 6)	Placebo (*n* = 8)	Cohort 11 (120 mg, *n* = 6)	Placebo (*n* = 2)
Any	2 (33.3)	1 (16.7)	3 (50.0)	3 (60.0)	2 (33.3)	2 (33.3)	4 (66.7)	1 (8.3)	2 (33.3)	0	1 (16.7)	2 (33.3)	3 (37.5)	5 (83.3)	2 (100.0)
Serious	0	0	0	0	0	0	0	0	0	0	0	0	0	0	0
**TEAEs occurring in more than one participant across the cohorts**															
Dizziness							1 (16.7)							1 (16.7)	
Conjunctival hyperemia							1 (16.7)					1 (16.7)			
Dry eye					1 (16.7)		1 (16.7)								
Rhinorrhoea						1 (16.7)	1 (16.7)		1 (16.7)						
Cough		1 (16.7)							1 (16.7)						
Nasal congestion						1 (16.7)	2 (33.3)								
Oropharyngeal pain		1 (16.7)										1 (16.7)			
Abdominal discomfort														2 (33.3)	1 (50.0)
Diarrhea														2 (33.3)	1 (50.0)
White blood cell count increased					1 (16.7)								1 (12.5)		
Neutrophil count increased			1 (16.7)	2 (40.0)	1 (16.7)								1 (12.5)		
Red blood cell count decreased														1 (16.7)	1 (50.0)
Hematocrit decreased														2 (33.3)	1 (50.0)
**TEAEs of special interest without occurrence across cohorts**															
Somnolence							2 (33.3)								
Hypersomnia											1 (16.7)				
Ocular discomfort					1 (16.7)										
Swelling of the eyelid					1 (16.7)										
Constipation														2 (33.3)	

All data are presented as the number of subjects (%). The number of randomized participants who received at least one dose of KDS2010 or placebo was indicated as *n*

Following a single oral administration, KDS2010 reached its maximum plasma concentration (C_max_) within a median of 3 h (Fig. 5b, Supplementary Table 4). The concentration declined biexponentially with an average terminal half-life of 44.2 h. In the 120 mg group, the area under the curve extrapolated to infinity (AUC_inf_) was 13850.7 ng*h/mL on average. Less than 1% of the unchanged drug was excreted in the urine. The metabolite showed, on average, 8.54-fold higher systemic exposure than KDS2010. A high-fat meal did not affect systemic exposure (C_max_ and AUC_inf_) after administration of KDS2010 120 mg (Fig. 5d). After 7 days of multiple oral administrations, the AUC during the dosing interval at steady state (AUC_τ,ss_) was, on average, 3.26-fold higher than that observed on day 1 (Fig. 5c). The systemic exposure (AUC_inf_ and AUC_τ,ss_) increased in a dose-proportional manner from 60 mg to 480 mg. Elderly subjects exhibited approximately 25% higher exposure: geometric mean ratio (GMR) of C_max_ and AUC_τ,ss_ were 1.2521 and 1.2525, respectively (Fig. 5e). However, the inactive metabolite was produced less in the elderly compared to healthy young subjects, with an average metabolic ratio of 4.69 and 7.69 at steady state, respectively. The pharmacokinetic profiles were similar between Korean and Caucasian subjects (Fig. 5f).

## Discussion

In this study, we discovered that GABA from scar-forming reactive astrocytes acts as a brake on the CNS repair system in severe SCI animal models. We could liminate this brake through genetic or pharmacological inhibition of MAOB, leading to significant functional and tissue recovery after SCI. On the basis of these findings, we propose the following mechanistic model. Astrocytic MAOB upregulation leads to excessive GABA production via polyamine degradation, resulting in enhanced tonic inhibition of neighboring neurons and suppression of proBDNF expression. The decrease in proBDNF levels impairs neuroregeneration and functional recovery after SCI. Conversely, the inhibition of MAOB restores proBDNF signaling and promotes neuronal survival and tissue repair. Our data further indicate that astrocytic BDNF acts as a downstream effector of the MAOB-GABA pathway, as astrocyte-specific BDNF deletion did not affect GABA production or astrocyte reactivity, but significantly impaired recovery. While combining astrocyte-specific MAOB and BDNF knockouts would provide definitive validation of this pathway, the generation of such double conditional knockout animals would require extensive breeding and validation over more than a year, which was beyond the feasible scope of this study. We recognize the importance of this approach and are considering it for future investigations to more precisely dissect the MAOB-GABA-BDNF signaling axis in SCI. Collectively, our findings strongly support a mechanistic link wherein astrocytic GABA acts as an upstream negative regulator of BDNF signaling following SCI. While our data converge across multiple experimental approaches, direct confirmation of causality through functional rescue experiments or combined genetic manipulations will be necessary in future studies.

While our data support a critical role of the astrocytic MAOB-GABA-proBDNF axis in SCI recovery, it is important to address the broader historical context regarding the role of MAOB in catecholamine metabolism. Importantly, although MAOB has historically been linked to catecholamine metabolism, recent studies have clarified that MAOA, rather than MAOB, predominantly degrades dopamine and other catecholamines. In contrast, MAOB mediates astrocytic GABA synthesis via the putrescine degradation pathway, particularly in the CNS.^42^ On the basis of this updated understanding, our study focused specifically on the astrocytic MAOB–GABA axis as the main pathological mechanism underlying impaired neuroregeneration following SCI. Therefore, we did not measure catecholamine levels. In addition, although systemic drug delivery could theoretically affect multiple regions, our analyses—including immunohistochemistry, electrophysiology, and molecular studies—demonstrated a spinal cord–specific modulation of GABA, proBDNF, and TrkB signaling following MAOB inhibition. These findings suggest that the observed effects predominantly reflect local spinal cord mechanisms rather than systemic monoamine alterations.

Consistent with this mechanistic model, the selective MAOB inhibitor, KDS2010, causes robust neuroregeneration with neuronal proliferation near the injury site, along with strong induction of astrocytic proBDNF and neuronal TrkB, which can contribute to the regenerative process. Importantly, the safety of this drug has been confirmed in the phase 1 clinical trial. These novel findings provide a comprehensive understanding of the intricate interplay between astrocytes and regenerative processes specific to SCI.

BDNF functions as the mature form, mBDNF, which is processed from proBDNF by tissue plasminogen activator (tPA) in the extracellular space or prohormone convertase 1/3 (PC1/3) in intracellular vesicles.^43,44^ Upon binding to TrkB, mBDNF activates Akt (protein kinase B; PKB) through PI3K signaling in postsynaptic cells,^45,46^ enhancing neuroregeneration by improving cell survival, differentiation, and synaptic plasticity while inhibiting apoptosis.^47–49^ Moreover, numerous reports emphasize its importance in SCI.^38,50,51^ Thus, it is not surprising that BDNF‒TrkB signaling is critically involved in neuroregeneration and functional recovery after SCI. However, the precise molecular mechanism by which proBDNF expression can be induced in astrocytes is still not clear. So far, we only know that GABA can suppress the BDNF mRNA and proBDNF protein expression in spinal cord astrocytes (Fig. 3h–l). Many blood-borne serine proteases have been reported to rush into injury sites and activate the protease-activated receptor 1 (PAR1) in astrocytes.^52,53^ These raise the possibility that PAR1 activation can induce BDNF mRNA and proBDNF protein expression. Indeed, PAR1 activation has been shown to induce BDNF release in blood platelets.^54^ Thus, future experiments are needed to identify BDNF-inducing factors and determine the precise mechanism of BDNF induction during SCI.

In most cases, MAOB inhibition with KDS2010 is sufficient to promote recovery after SCI and reduce reactive astrogliosis. However, when KDS2010 was administered from the acute phase, it failed to achieve this in some animals (Supplementary Fig. 7). These results raise intriguing questions concerning why the MAOB inhibitor works in most animals but not in some animals. We have previously reported that DAO, as an alternative pathway to MAOB, can produce GABA^24,55^ and that MAOB-dependent astrocytic H_2_O_2_ causes reactive astrogliosis in animal models of Alzheimer’s disease.^55^ Therefore, it is possible that MAOB might be dominantly expressed and active over DAO in most cases of SCI, whereas in other cases, DAO might be dominant over MAOB and continue to produce H_2_O_2_ to enhance reactive astrogliosis and GABA to inhibit proBDNF even in the presence of KDS2010. Future work is needed to explore these exciting possibilities using effective DAO inhibitors.

Numerous studies have reported that reactive astrocytes release cytokines and CSPGs, which are proposed to act as physical and chemical barriers to CNS axon extension.^8,9^ In this study, we assessed the expression of two key CSPG family members—neurocan and phosphacan—using immunohistochemistry and Western blotting. We found that CSPG expression was significantly increased following SCI, whereas KDS2010 treatment notably reduced CSPG expression in the lesion penumbra (Supplementary Fig. 9d–h). This reduction closely paralleled the suppression of GFAP-positive astrocyte reactivity. These findings support the interpretation that astrocytic MAOB inhibition reduces glial reactivity, leading to a more permissive extracellular environment by downregulating CSPG expression, thereby contributing to enhanced neuronal preservation and potential regeneration. Thus, it will be interesting to explore future work combining KDS2010 with chondroitinase ABC (ChABC), an agent currently under clinical investigation for SCI.

The translational relevance of our findings is particularly strong, as the effects of MAOB inhibition are consistently observed across species and various SCI recovery phases. The robust preclinical evidence supporting the efficacy of KDS2010 in both rodents and nonhuman primates suggests that its clinical application could yield substantial benefits for SCI patients. However, some limitations must be acknowledged. While our study establishes a strong correlation between neuroregeneration and functional recovery, future experiments using chemogenetic or optogenetic tools could help clarify the causal relationship between these processes, providing deeper insights into the precise mechanisms by which KDS2010 promotes CNS repair.

On the basis of the preclinical studies and phase 1 clinical trials, the effective dose of KDS2010 is anticipated to be 120 mg. The pharmacologically active dose (PAD) was 10 mpk for 8 weeks for SD rats and 3 mpk for 5 weeks for cynomolgus macaques. In each animal model, the systemic exposure (AUC_last_) was approximately 3000 and 13,000 ng*h/mL, respectively. The dose range for phase 1 clinical trial was determined using a pharmacokinetic model based on preclinical plasma concentration data. The model consisted of two compartments with a clearance proportional to the 0.75^th^ power of the body weight and the volume of distribution to the body weight. In healthy humans, the administration of 120 mg of KDS2010 achieved the target therapeutic exposure of preclinical PAD, with an AUC_inf_ of 13850.7 ng*h/mL on average.

At higher doses (240–960 mg), TEAEs related to the autonomic nervous system were observed. MAO oxidatively deaminates endogenous monoamines, including norepinephrine, dopamine, and serotonin.^56^ Consequently, neurotransmitter imbalance has been suggested as a potential cause of adverse events associated with MAO inhibitors.^57^ The sleep disturbances, dry mouth, and constipation observed in this study have also been reported with other traditional MAOB inhibitors.^58–60^ The autonomic nervous system-related adverse events were reported to be reversible,^61^ which is consistent with the findings of this study.

In a phase 2 clinical trial, the functional recovery effect of KDS2010 120 mg will be explored in SCI patients. When recruiting the SCI patients, stratification by injury severity and phase (acute, subacute, and chronic) could be helpful. Standardized diagnostic tools, such as the American Spinal Injury Association (ASIA) Impairment Scale, will help accurately assess the degree of injury and functional impairment. Primary endpoints should focus on functional recovery, including motor and sensory improvements, while secondary endpoints could assess pain, quality of life, and autonomic function. Integrating rehabilitation therapies in the study will be crucial to understanding how KDS2010 works with standard care, potentially maximizing recovery outcomes. By addressing these factors, the phase 2 trial will provide vital insights into the clinical efficacy and broader therapeutic potential of KDS2010 for SCI patients.

In summary, we explored the unexplored territory of scar-forming reactive astrocytes and identified MAOB-dependent GABA as the key molecular brake that masks the regenerative process of the mammalian spinal cord after injury. Notably, KDS2010 has shown promise in preclinical studies involving rodent and nonhuman primate models and was confirmed to be safe in a phase 1 clinical trial. We hope that the newly developed concepts and tools related to SCI will be therapeutically helpful in clinical settings where there is currently no available option.

## Materials and methods

### Animals

All animals were housed in facilities accredited by the Association for Assessment and Accreditation of Laboratory Animal Care (AAALAC) at each center. All experimental procedures with mice and rats were conducted following protocols approved by the Institutional Animal Care and Use Committee (IACUC) of Yonsei University (Protocol No. 2016-0027; Seoul, Republic of Korea) and the Institute for Basic Science (Protocol No. IBS-2023-004; Daejeon, Republic of Korea). *B6;129S-Maob*^*tm1Shih*^*/J* (MAOB KO; RRID:IMSR_JAX:014133, The Jackson Laboratory, Bar Harbor, ME, USA) mice, which are X-linked *MAOB* gene-deficient mice, were backcrossed with 129S4/SvJaeJ (The Jackson Laboratory) for more than 10 generations and were thought of as congenic to 129S4/SvJaeJ. They (8-week-old; 20 g ± 3 g) were used for SCI experiments. 129S4/SvJaeJ were used as controls. B6-*Maob*^*em1Cjl*^/Ibs (*Maob* floxed) was generated from Cyagen Biosciences (Guangzhou, China). Female heterozygous *Maob* floxed mice (B6-*Maob*^*em1Cjl*^/Ibs) were crossed with male transgenic hGFAP-CreER^T2^ (B6-Tg(GFAP-cre/ERT2)13Kdmc; MGI:3712447) mice to generate male *Maob* floxed::hGFAP-CreER^T2^ mice as previously described.^62^ Since *Maob* is located on the X chromosome, male mice carrying a single floxed allele undergo complete gene inactivation upon Cre-mediated recombination. Adult (7-week-old) *Maob* floxed::hGFAP-CreER^T2^ mice were treated with tamoxifen at 100 mg/kg once per day for 5 days by intraperitoneal (IP) injection to generate astrocyte-specific MAOB cKO mice. Tamoxifen was dissolved in sunflower oil containing 10% ethanol at a concentration of 20 mg/mL. For the control mice, the same amount of sunflower oil was injected into the same age of *Maob* floxed::hGFAP-CreER^T2^ mice. One week after injection, they (8-week-old; 20 g ± 3 g) were used for the SCI experiments. For astrocyte-specific transgene expression of human MAOB in C57BL/6-Tg(Gfap-rtTA,tetO-MAOB,-lacZ)1Jkan/J (GFAP-MAOB; The Jackson Laboratory, 8-week-old; 20 g ± 3 g) mice, the animals were fed with doxycycline at 3000 ppm provided in pre-mixed Purina chow (Research Diets) for three weeks.^63^ 129S4/SvJae-Bdnf^*tm3Jae*^/J (*Bdnf* floxed; The Jackson Laboratory) were backcrossed with C57BL/6J for more than 10 generations and were thought to be congenic to C57BL/6J. To generate astrocyte-specific BDNF cKO mice, homozygous B6-*Bdnf* floxed mice were crossed with hGFAP-CreER^T2^ or Aldh1l1-Cre/ER^T2^ mice.^64^ We used the C57BL/6J strain of Aldh1l1-Cre/ER^T2^ by backcrossing B6N.FVB-Tg(Aldh1l1-cre/ERT2)1Khakh/J (The Jackson Laboratory) with C57BL/6J for more than 10 generations. Tamoxifen or saline was injected via the same procedures as those used in the astrocyte-specific MAOB cKO experiment. Sprague-Dawley (SD) rats (Orient Bio, Republic of Korea; 8-week-old; 200 g ± 20 g) were used for the SCI experiments. Additionally, female cynomolgus monkeys (3 kg; aged 3–4 years) were used for the SCI experiments. Unless otherwise specified, all mouse and rat experiments were conducted using male animals, and all nonhuman primate experiments were conducted using female cynomolgus monkeys. All procedures involving monkeys were approved by the IACUC of the Korea Institute of Toxicology (KIT) (Approval No. B216074; Jeongeup-si, Republic of Korea).

For astrocyte-specific deletion of target genes, we utilized two inducible Cre driver lines: hGFAP-CreER^T2^ and Aldh1l1-CreER^T2^. hGFAP-CreER^T2^ mice were used for functional analyses, including behavioral assessments and histological evaluations, to selectively target reactive astrocytes where GFAP promoter activity is elevated under injury conditions. In complementary validation experiments, Aldh1l1-CreER^T2^ mice were employed to achieve broader recombination across both resting and reactive astrocytes, reflecting Aldh1l1’s pan-astrocytic expression profile.

### Primary mouse spinal cord astrocyte culture

Primary cultured astrocytes were prepared from the spinal cords of C57BL/6J mouse (Jackson Laboratory) pups (P0-P2). The entire spinal cord was dissected by removing all the dorsal root ganglia, and the meninges covering the outer surface of the spinal cord were removed. The dissected spinal cord was minced and dissociated into a single-cell suspension via trituration. Cells were plated onto cell culture dish coated with 0.1 mg/mL poly D-lysine (PDL, P6407, Sigma-Aldrich). The cells were cultured in Dulbecco’s modified Eagle’s medium (DMEM, 10-013, Corning) supplemented with 25 glucose, 4 L-glutamine, 1 sodium pyruvate (in mM), 10% heat-inactivated horse serum (26050-088, Gibco), 10% heat-inactivated fetal bovine serum (10082-147, Gibco) and 100 units/mL penicillin–streptomycin (15140-122, Gibco). Cultures were maintained at 37 °C in a humidified 5% CO_2_ incubator. After 3 days in vitro (DIV), cells were vigorously washed with repeated pipetting, and the media was replaced to get rid of debris and other floating cell types. While the culture was maintained before use, the media was replaced every 3–4 days.

### Establishment of the SCI crush model

For the establishment of the mouse and rat SCI models, we followed methods consistent with those of previous studies.^65–67^ In mice, a laminectomy was performed at thoracic level 10 (T10), and self-closing forceps (11485-11, Fine Science Tools; 0.10 × 0.06 mm tip, 11 cm) were applied to the exposed spinal cord for 3 s to induce injury. In rats, a laminectomy was performed at thoracic level 9 (T9), followed by the application of the same self-closing forceps (11485-11, Fine Science Tools) for 10 s. Each “Sham” group or “SCI” group indicates animals operated by only laminectomy or SCI, respectively.

For the nonhuman primate SCI model, female cynomolgus macaques were anesthetized with Zoletil 50 (8–10 mg/kg; Virbac Korea) and isotropy 100. Laminectomy was performed at thoracic level 8 under X-ray guidance, and the SCI model was established using a vessel clip (Micro Serrefine Clamp, 1805501, Fine Science Tools; 60 g pressure, 19 mm) applied for 10 min. After surgery, the monkeys were monitored until full recovery, with precautions taken to prevent hypothermia and dehydration. For pressure ulcers, antibacterial drugs were administered, and sugar therapy was applied as needed.

Owing to interspecies variation in spinal cord-to-vertebral column alignment, the spinal segment-to-vertebra ratio (0.83–0.85) was considered to ensure comparable targeting of the lumbosacral enlargement responsible for hindlimb motor control. Specifically, injuries were induced at T10 in mice (13 thoracic vertebrae × 0.85 ≈ T10), T9 in rats (13 thoracic vertebrae × 0.83 ≈ T9), and T8 in monkeys (12 thoracic vertebrae × 0.83 ≈ T8). These injury levels correspond to the thoracolumbar transition zone or mid-thoracic regions that include descending motor pathways, achieving functionally equivalent hindlimb impairment across species, which is consistent with established protocols.

### Drug delivery

(S)-2-(((4’-Trifluoromethylbiphenyl-4-yl)methyl)amino)propanamide methane-sulfonate (KDS2010) was dissolved in distilled water. For SD rats, KDS2010 was administered via the drinking water ad libitum. The rats received 10 mg/kg/day KDS2010 starting from the acute (PI 1 day), subacute (PI 2 weeks), or chronic phases (PI 6 weeks). To evaluate dose-dependent effects, additional groups received 20 mg/kg/day or 30 mg/kg/day starting at PI 2 weeks. To achieve consistent exposure across animals, we meticulously monitored the daily water intake and body weight of each rat throughout the study. As shown in the Extended Data (Supplementary Table 1), water consumption was calculated weekly via a formula based on body weight (e.g., d.w = weight × 0.09 at week 0; gradually tapering to weight × 0.075 by weeks 8–9). Importantly, all animals were confirmed to have consumed the entirety of the provided medicated water each day, ensuring full ingestion of the intended dose (10 mg/kg/day for the majority of groups, and 20/30 mg/kg/day for the dose-dependent experiments).

For cynomolgus macaques, KDS2010 was administered daily via oral gavage beginning 1 day after injury. To determine the appropriate dosing for macaques, interspecies dose translation was performed according to the U.S. FDA’s Guidance for Industry (2005), using body surface area (BSA)-based conversion with species-specific Km values. Using a Km of 6 for rats and 12 for cynomolgus monkeys, the human equivalent dose (HED) for monkeys was calculated as:

$${\mathrm{HED}}_{\mathrm{monkey}}=\mathrm{Rat\; dose\; x}\left(\frac{Kmrat}{Kmmonkey}\right)=\frac{10{mg}}{{kg}}\times \left(\frac{6}{12}\right)=5{mg}/{kg}$$

On the basis of this calculation, two doses were selected: 3 mg/kg/day (~60% of the HED) for evaluating the minimal effective dose, and 10 mg/kg/day (approximately 2× HED) for assessing upper tolerability and therapeutic margin. This dose selection also considered species-specific pharmacokinetics, as the apparent clearance (CL/F) in monkeys (0.228 L/h/kg) was markedly lower than that in rats (1.140 L/h/kg). At 10 mg/kg in monkeys, the AUC was 43,623 ng·h/mL, comparable to that observed in rats at 20 mg/kg. The administration of 30 mg/kg in monkeys led to a nonlinear increase in the AUC (~4.6-fold), suggesting saturation kinetics and a potential risk of drug accumulation (Supplementary Table 2).

### Viruses and stereotaxic surgery

Polymerase chain reaction (PCR) was employed to amplify the promoter region of the rat *Mki67* gene, encoding the proliferation marker protein Ki67, using rat genomic DNA (69238-3CN, Novagen) as a template. The amplified sequences comprised the 1.4 kb putative rKi67 promoter region, including sequences upstream of the transcription start site. The Ki67 (rKi67) promoter, EGFP, and Cre recombinase genes were incorporated into the AAV-rKi67-EGFP-Cre viral vector, which was then cloned into the AAV-MCS (multiple cloning site) expression vector (VPK-410, Cell Biolabs, Inc.) via the In-Fusion method (639649, Clontech). The AAV-EF1α-DIO-mCherry viral vector was purchased (47636, Addgene). These viral vectors were subsequently purified by iodixanol gradients at the KIST Virus Facility.

For validation of the Ki67 promoter, we injected 2 μL of AAV- DJ-rKi67-EGFP-Cre (4.58 × 10^13^ genome copies per milliliter (GC/mL)) and AAV-DJ-Ef1α-DIO-mCherry (5.56 × 10^13^ GC/mL) at a 1:1 ratio bilaterally into the dentate gyrus (AP = −3.8 mm, ML = 1.8 mm, DV = 3.5 mm) of SD rats (7 weeks old). Two weeks after virus injection, the rats were sacrificed for immunohistochemistry.

To label the proliferating and proliferated cells after SCI via the Ki67 promoter, we injected 3 μL of AAV-rKi67-EGFP-Cre (4.58 × 10^13^ GC/mL) and AAV-Ef1α-DIO-mCherry (5.56 × 10^13^ GC/mL) with at 1:1 ratio into the spinal cord at two locations (1 mm proximal and distal to the injured site) into the SCI model 2 weeks after SCI. At PI 10w (8 weeks after virus injection), rats were sacrificed for immunohistochemistry.

### Behavioral test

Behavioral test was performed every week for a total of 11 weeks (1 week before and 10 weeks after the surgical operation) to assess locomotor recovery. The BBB motor score^22^ or BMS score^17^ was used to evaluate the quality of hindlimb movement during open field locomotion in rats or mice, respectively. In the first recovery phase, the range of joint movement and the presence of the foot closure on the floor were checked. In the second phase, the recovery of weighted stepping was checked. In the third phase, gait coordination and tail movement were assessed. These scores are “ordinal and the magnitude of behavioral change between ranks may not be consistent,” as originally described.^17^ To perform the group allocation in a blinded manner during data collection, animal preparation and behavior tests were performed by at least two different investigators.

In experiments involving acute KDS2010 administration and transgenic mouse models, animals were grouped on the basis of similar average BBB or BMS scores one day post-injury. In the BBB locomotor test with drug administration beginning at PI 2w, the animals were initially subjected to injury, and then divided into two groups at PI 2w based on the basis of similar averaged BBB scores. Subsequently, these groups were treated with either the vehicle (SCI + V) or the drug (SCI+drug) from PI 2w. To exclude incompletely generated severe rat SCI animals, we established the following criteria: a score difference of more than 4 between PI 1w and 2w, and a score exceeding 8 at PI 2w. In the BMS locomotor test, we excluded animals that did not score 0 one day after SCI, as previously described.^68^

The ladder rung test was performed with an apparatus featuring a 100 cm horizontal ladder with a 1 cm spacing between rungs. Functional recovery was measured by the percentage of hindlimb steps without slipping as the rats walked along a horizontal ladder, as previously reported.^23,69^

### Immunohistochemistry

Immunohistochemistry was performed as previously described.^70^ The tissue sections were blocked and treated with primary antibodies. After overnight incubation at 4 °C, species-specific fluorescent secondary antibodies were applied for visualization. The tissue sections were then stained with 4’,6-diamidino-2-phenylindole (DAPI) during mounting. This protocol allows a detailed assessment of various cellular markers involved in SCI and recovery processes.

The primary antibodies used included chicken anti-MAP2 (1:2000, ab5392, Abcam), goat anti-GFAP (1:1000, ab53554, Abcam), rabbit anti-MAOB (1:200, ab175136, Abcam), mouse anti-GABA (1:1000, ab86186, Abcam), chicken anti-GFP (1:500, ab13970, Abcam), rabbit anti-mCherry (1:500, ab167453, Abcam), rabbit anti-Ki67 (1:500, ab16667, Abcam), rabbit anti-NeuN (1:800, ab177487, Abcam), chicken anti-proBDNF (1:200, AB9042, Millipore), rabbit anti-TrkB (1:100, ab18987, Abcam), chicken anti-Tau (1:200, ab75714, Abcam), chicken anti-Myelin basic protein (1:400, ab134018, Abcam), rabbit anti- Tuj1 (1:2000, ab18207, Abcam), and rabbit anti-DAO (1:500, ARP41908_P050, Aviva Systems Biology).

The secondary antibodies used included FITC-donkey anti-mouse IgG (H + L) (1:150, 715-095-151), Cy^TM^3-donkey anti-mouse IgG (H + L) (1:600, 715-165-151), FITC-donkey anti-rabbit IgG (H + L) (1:150, 711-095-152), Alexa Fluor 594-donkey anti-rabbit IgG (H + L) (1:500, 711-585-152), Alexa Fluor 647-donkey anti-rabbit IgG (H + L) (1:500, 711-605-152), Cy^TM^3-donkey anti-chicken IgG (H + L) (1:600, 703-165-155), DyLightTM405-donkey anti-chicken IgY + + (H + L) (1:600, 703-476-155), Alexa Fluor 488-donkey anti-chicken IgY (IgG) (H + L) (1:500, 703-545-155), Alexa Fluor 594-donkey anti-chicken IgY (IgG) (H + L) (1:500, 703-585-155), Alexa Fluor 488-donkey anti-goat IgG (H + L) (1:500, 705-545-003), and Alexa Fluor 647-donkey anti-goat IgG (H + L) (1:300, 705-606-147). All secondary antibodies were purchased from Jackson ImmunoResearch.

The fluorescence images were observed via confocal laser scanning microscopy (LSM700 or LSM710, Carl Zeiss), and analyzed via the ImageJ (NIH) software. For visualizing the mCh-positive signals of proliferating or proliferated cells, Z-stack images acquired in 1-μm steps were processed for 3D surface rendering of mCh-positive signals using IMARIS software (version 9.0.1, Oxford Instruments).

### EC staining

EC staining was performed as previously described.^71^ The tissue sections were placed on a microscope slide, allowed to dry for 2 h, and then immersed in acetone for 5 min. The slides were incubated for 10 min at 22 ± 2 °C and then treated with EC solution (MERCK, Kenilworth, NJ, USA) for 30 min at 22 ± 2 °C. After the sections were rinsed with running water, they were placed on 5% iron alum (Sigma-Aldrich, F3629) until the gray matter was observed. The sections were differentiated using borax-ferricyanide solution (Borax, Sigma-Aldrich, 71997; Potassium ferricyanide, Sigma-Aldrich, 702587) and dehydrated using graded ethanol solutions of 70%, 90%, and 100% which were changed twice. The sections were mounted with permanent mounting medium (Fisher Scientific, Hampton, NH, USA) and finally observed under a light microscope (DM 2500, Leica, Wetzlar, Germany).

### Luxol fast blue (LFB) staining

LFB staining was performed as previously described.^70^ The myelin sheath of axons in the tissue sections were stained with 1% luxol fast blue (Sigma-Aldrich, L0294) at 60 °C overnight. The sections were rinsed with lithium carbonate (Sigma-Aldrich, 255823) solution and differentiated by 70% alcohol and then observed under a light microscope (IX71, Olympus, Tokyo, Japan).

### TB staining and TEM

Spinal cord tissues were prefixed in Karnovsky’s fixative (2% glutaraldehyde, 2% paraformaldehyde in 0.1 M phosphate buffer) overnight at 4 °C. After postfixation in 1% osmium tetroxide (OsO_4_), the samples were dehydrated through a graded ethanol series and embedded in epoxy resin.

For TB staining, 250–300 nm semi-thin sections were obtained via an ultramicrotome (Leica Ultracut UCT, Leica Microsystems, Austria), stained with 0.5% toluidine blue, and imaged using a light microscope (Olympus IX71).

For TEM, 80–100 nm ultrathin sections were cut using a diamond knife (Diatome, Switzerland), mounted on 300-mesh Gilder grids, and stained with 6% uranyl acetate and lead citrate. Images were acquired using a transmission electron microscope (JEM 1011, JEOL, Tokyo, Japan) as previously described.

All TB and TEM analyses were performed on spinal cord tissues collected at the lesion epicenter, where myelination is most affected by injury.

### Tonic GABA current recording

Prior to recording, spinal cords were harvested from rats that were anesthetized with a mixture of Zoletil and Xylazine. The dura mater of the isolated spinal cords was eliminated in the ice-cold recovery solution containing N-methyl-D-glucamine (NMDG) (NMDG-recovery solution): 93 NMDG, 2.5 KCl, 1.2 NaH_2_PO_4_, 30 NaHCO_3_, 20 HEPES, 25 D-(+)-glucose, 5 sodium ascorbate, 2 thiourea, 3 sodium pyruvate, 10 MgSO_4_, and 0.5 CaCl_2_ (in mM), pH 7.4. The slices (400 μm in thick) were first incubated at 32 °C for 15 min in NMDG-recovery solution, and then incubated at 22 ± 2 °C for at least 1 h in normal artificial cerebrospinal fluid (aCSF) solution: 130 NaCl, 24 NaHCO_3_, 1.25 NaH_2_PO_4_, 3.5 KCl, 1.5 CaCl_2_, 1.5 MgCl_2_, and 10 D-(+)-glucose (in mM), pH 7.4. For the recordings, the slices were transferred to a recording chamber that was continuously perfused with aCSF solution. Whole-cell patch-clamp recordings were performed on the neurons of lamina II in the dorsal horn of the spinal cord within 2 cm of the injury. The holding voltage was −60 mV. The pipette resistance was typically 6–8 MΩ, and the pipette was filled with an internal solution: 135 CsCl, 4 NaCl, 0.5 CaCl_2_, 10 HEPES, 5 EGTA, 4 Mg-ATP, 0.3 Na_2_-GTP, and 10 QX-314 (mM), with the pH adjusted to 7.2 with CsOH (278–285 mOsmol/kg). Electrical signals were digitized and sampled at 50-ms intervals with a Digidata 1440 A and Multiclamp 700B amplifier (Molecular Devices) using pCLAMP 10.4 software (Molecular Devices). The data were filtered at 2 kHz. D-AP5 (50 μM; 0106, Tocris), CNQX (20 μM; 0190, Tocris), GABA (10 μM; A2129, Sigma-Aldrich), and (-)-bicuculline methobromide (50 μM; BIC; 0109, Tocris) were treated for measuring tonic GABA current and fully-activated GABA current as previously described.^11^ The measurement was performed using the Clampfit 10.4 program (Molecular Devices). Every current was divided by the capacitance of each cell to calculate the current density. The frequency and amplitude of sIPSCs before GABA application were measured by Mini Analysis Program (Synaptosoft). For the analysis, data points were excluded when the membrane capacitance of the cell was under 5 pF. Finally, Grubb’s test was performed to identify statistical outliers.

### Quantitative real-time PCR

Primary mouse spinal cord astrocytes were treated with GABA (100 μM, A2129, Sigma-Aldrich) and/or bicuculline (50 μM; 0109, Tocris). Approximately 24 h after drug treatment, total RNA was extracted by using RNA isolation kit (314-150, GeneAll), and cDNA was synthesized by using reverse transcriptase (18080-044, Invitrogen). SYBR-green (4309155, Applied Biosystems) was used to perform quantitative real-time PCR. The following primer sequences^72^ were used for quantitative real-time PCR. Mouse BDNF forward: 5’-AAAGTCCCGGTATCCAAAGGCCAA-3’; Mouse BDNF reverse: 5’- TAGTTCGGCATTGCGAGTTCCAGT-3’. Mouse GAPDH forward: 5’- TGATGACATCAAGAAGGTGGTGAAG-3’; Mouse GAPDH reverse: 5’- TCCTTGGAGGCCATGTAGGCCAT-3’.

### Western blot and ELISA

Approximately 24 h after drug treatment, primary mouse spinal cord astrocytes or spinal cord tissues were lysed in RIPA buffer (MB030-0050, ROCKLAND) containing protease and phosphatase inhibitors (1861280, Thermo Fisher Scientific). The protein concentration was measured using the Pierce™ BCA Protein Assay Kit (Thermo Fisher Scientific). Each sample was then used separately for Western blot or GABA ELISA analysis.

Western blot analysis was performed following established protocols,^73,74^ with 20 μg of spinal cord tissue or 10 μg of astrocyte protein samples were loaded onto SDS-PAGE gels, transferred to PVDF membranes, and blocked with 5% non-fat milk. The membranes were incubated overnight at 4 °C with primary antibodies, including rabbit anti-MAOB (1:1000, Abcam), rabbit anti-BDNF (1:1000, Abcam), mouse anti-TrkB (1:1000, R&D Systems), and rabbit anti-β-actin (1:1000, Cell Signaling Technology). Detection was achieved using species-specific secondary antibodies conjugated with horseradish peroxidase (HRP), including anti-rabbit IgG, HRP-linked (1:2500 or 1:5000, 7074, Cell Signaling Technology), anti-mouse IgG, HRP-linked (1:2500, 7076, Cell Signaling Technology), and rabbit anti-goat IgG, HRP-linked (1:2500, ab6741, Abcam). The bands were visualized using enhanced chemiluminescence, and band intensity was quantified using ImageJ software.

For enzyme-linked immunosorbent assay (ELISA), GABA quantification was performed using an ELISA kit (BA-E-2500, ImmuSmol) according to the manufacturer’s instructions, utilizing 20 μg of protein extracted from spinal cord tissues.

### Evaluation of liver function test parameters in nonhuman primates

Blood samples were collected from the animals via the radial or femoral vein at three time points: 2 days, 2 weeks, and 5 weeks post-injury. All the animals were fasted for approximately 16 h prior to each collection, with water provided ad libitum. Approximately 2.0 mL of blood was drawn and placed into tubes without anticoagulant and left at room temperature for at least 90 min. The samples were then centrifuged at 3000 rpm for 10 min at room temperature to separate the serum. Finally, the serum samples were analyzed for creatinine (CREA), alkaline phosphatase (ALP), gamma-glutamyl transpeptidase (GGT), total bilirubin (TBIL), ALB, ALT, and aspartate aminotransferase (AST) levels. These biochemical parameters were measured using a TBA 120FR chemistry analyzer (Toshiba Co.).

### Phase 1 clinical trial study design and subjects

A randomized, double-blind, placebo-controlled study of oral KDS2010 was conducted in healthy Korean and Caucasian subjects (Clinical Research Information Service registry no. KCT0008331). All subjects provided consent for this phase 1 clinical trial study. The study comprised 11 cohorts: single ascending dose (SAD) and multiple ascending dose (MAD) studies. SAD cohorts (1–9) received 30–960 mg of KDS2010. Cohort 3 employed a two-period crossover design with a 14-day washout. Participants received a 120 mg dose of KDS2010 after a 10-h fast in the first period, and following a high-fat meal (over 900 kcal, 35% fat) in the second. This allowed for a direct comparison of KDS2010 pharmacokinetics in fasting versus fed states. MAD cohorts (7–10) received 60–480 mg of KDS2010 daily for 7 days. Cohort 11 comprised elderly subjects who received 120 mg of KDS2010 daily for 7 days.

Subjects were randomized 3:1 to receive KDS2010 or placebo. Eligibility criteria included healthy adults (aged 19–45) for the SAD and MAD cohorts, and healthy elderly subjects (aged 65–85) for cohort 11. The participants were excluded if they met the following criteria: serum AST or ALT greater than 60 IU/L, total bilirubin greater than 1.8 mg/dL, creatinine phosphokinase (CPK) levels greater than 405 IU/L, creatinine clearance calculated by the Chronic Kidney Disease Epidemiology Collaboration (CKD-EPI) equation less than 60 mL/min/1.73 m². At the time of randomization, all subjects were required to be free from any clinically significant medical conditions. The study was approved by the Seoul National University Hospital institutional review board and conducted in accordance with the Declaration of Helsinki and Good Clinical Practice guidelines.

Subjects who met the eligibility criteria were admitted to the Seoul National University Hospital Clinical Trial Center one day prior to the first dose administration. On the scheduled dosing day, participants received either KDS2010 or a placebo, randomly assigned, with 150 mL of water. After administration of the last dose, subjects were discharged 72 h later and visited the outpatient clinic for follow-up on three consecutive days. Pharmacokinetics and safety were monitored until the end of the study, lasting up to 18 days after the last dose.

The primary endpoint was the incidence of TEAEs by dose group. Any abnormalities in vital signs, electrocardiograms, clinical laboratory tests, or neurological examinations that occurred after KDS2010 administration were recorded as TEAEs. Those deemed to have a causal relationship with the drug were classified as ADRs. Dose escalation proceeded under blinded conditions, contingent on the absence of dose-limiting toxicity, as reviewed by a safety review committee.

Secondary endpoints included pharmacokinetic parameters such as maximum observed concentration (C_max_), time to Cmax (T_max_), area under the concentration-time curve from 0 to infinity (AUC_inf_), AUC during dosing intervals at steady state (AUC_τ,ss_), terminal half-life (t_1/2_), apparent clearance (CL/F), fraction excreted of the unchanged drug (fe), and renal clearance (CL_R_). The parameters related to plasma concentrations were calculated via non-compartmental analysis using the linear-up log-down method in Phoenix WinNonlin software version 8.3 (Certara, US). The parameters related to urine concentrations were calculated using the linear trapezoidal linear interpolation method.

Serial blood and urine samples were collected at the scheduled times, and the concentration of KDS2010 and its metabolite was measured. In the SAD study, plasma samples were collected using sodium K2-EDTA tubes at pre-dose, 0.33, 0.75, 1, 2, 3, 4, 6, 8, 12, 24, 48, 72, 96, 120, and 144 h after dosing. Urine samples were collected up to 72 h post-dose. In the MAD study, plasma samples were collected at pre-dose, 0.33, 0.75, 1, 2, 3, 4, 6, 8, 12 and 24 h after the administration of the first dose, and at pre-dose of the third to seventh doses. After the administration of the seventh dose, plasma samples were collected at the same time points as after the first dose, with additional sampling points at 48, 72, 96, 120, and 144 h post-dose. Urine samples were collected for 24 h after the administration of the first dose and for 72 h after the seventh dose.

The concentrations of KDS2010 in plasma and urine were determined using validated liquid chromatography–tandem mass spectrometry (SCIEX API 4000 system). Standard curves for KDS2010 showed linearity within a range of 5–5000 μg/L in plasma samples and 5–2500 μg/L in urine samples. The lower limit of quantification (LLOQ) for KDS2010 was 5 μg/L in both plasma and urine. For the metabolite, standard curves demonstrated linearity within a range of 1–1000 μg/L in both plasma and urine samples, with an LLOQ of 1 μg/L for both.

### Statistical analysis

For all preclinical experiments, the data are presented as the mean ± standard error of the mean (SEM). For all experiments, data normality was analyzed using a D’Agostino–Pearson omnibus normality test. For data not normally distributed, a Mann–Whitney test (two-tailed) or Kruskal–Wallis test with Dunn’s multiple comparison test was performed. Differences between groups were evaluated by one-way analysis of variance (ANOVA) with Tukey’s multiple comparisons tests. For the behavioral test, two-way RM ANOVA with Tukey’s multiple comparisons test and linear mixed model were performed for statistical analysis. Grubb’s test was performed to identify statistical outliers in tonic GABA recording experiment. GraphPad Prism 10.3.1 for Windows (GraphPad Software) and SAS 9.4 (SAS Institute Inc., USA) were used for these analyses.

For the clinical trial, dose proportionality of the PK parameters (C_max_, C_max,ss_, AUC_last_, and AUC_τ_) was assessed using power model using the following equation: log (PK parameter) = β0 + β1 log (dose). The regression slope (β1) and the intercept (β0) were estimated with 95% confidence interval (CI) using PROC REG in SAS version 9.4. The PK parameters were considered dose-proportional if the 95% CI of the β1 included 1. The effect of a high-fat meal on the PK parameters (C_max_, C_max,ss_, AUC_last_, and AUC_τ_) was evaluated by calculating the geometric mean ratio (GMR) and its 90% CI. Using a linear mixed effect model, the logarithmized PK parameters were analyzed with the high-gat meal as a fixed effect and inter-individual differences as a random effect. The GMR and its 90% CI of the PK parameters of the high-fat meal were calculated relative to those in the fasting state. The effect of a high-fat meal was considered negligible if the 90% CI fell within the range of 0.8 to 1.25. In elderly subjects, the same analyses were performed and compared to those of healthy younger adults.

## Supplementary information

supplementary data

Supplementary Movie 1

Supplementary Movie 2

uncropped western botting film

Clinical study protocol

## Data Availability

All data supporting the findings of this study are provided within the main text and Supplementary Information files.
