# Supplementary material for: Astrocytic monoamine oxidase B (MAOB)–gamma-aminobutyric acid (GABA) axis as a molecular brake on repair following spinal cord injury
Source: Signal Transduct Target Ther. 2025 Sep 11;10:295. doi: 10.1038/s41392-025-02398-2 (PMC12423301; doi:10.1038/s41392-025-02398-2)
Supplement: Supplementary file 1 — supplementary data [file 41392_2025_2398_MOESM1_ESM.docx]

**Supplementary information for**

Astrocytic monoamine oxidase B (MAOB)–gamma-aminobutyric acid (GABA) axis as a molecular brake on repair following spinal cord injury

Hye Yeong Lee^1,11^, Jung Moo Lee^2,11^_,_ Hye-Lan Lee^1,11^, Jiyeon Park^3,11^, Heeyoung An^2^, Eun Kyung Park^4^, Sae Yeon Hwang^1^_,_ Sol lip Yoon^1^, Gwang Yong Hwang^1^, Keung Nyun Kim^1^, Min-Ho Nam^5, 8^, Seung Eun Lee^6^, Hyunji Kang^2^, Joungha Won^2^, Bo Ko Jang^7,8^, Elijah Hwejin Lee^7,8^, SunYeong Choi^2,9^, Mingu Gordon Park^2^, Sang Wook Kim^4^, Ki Duk Park^7,8^, SeungHwan Lee^3^, C. Justin Lee^2,12,*^, Yoon Ha^1,10,*^

*Correspondence

Yoon Ha, MD, Ph.D.

Department of Neurosurgery, College of Medicine, Yonsei University,

Yonsei-ro 50, Seoul, Republic of Korea

Tel: +82-2-2228-2165, Fax: +82-2-2672-3638, E-mail: hayoon@yuhs.ac

C. Justin Lee, Ph.D.

Director of Center for Cognition and Sociality, Institute for Basic Science (IBS),

Expo-ro 55, Daejeon, Republic of Korea

Tel: +82-42-878-9150, Fax: +82-42-878-9151, E-mail: cjl@ibs.re.kr

**This PDF file includes:**

**Figures. S1 to S11**

**Tables S1 to 4**

**Captions for Movies S1 to S2**

**Other Supplementary Materials for this manuscript include the following:**

**Movies S1 to S2**

**Figures. S1.**

**
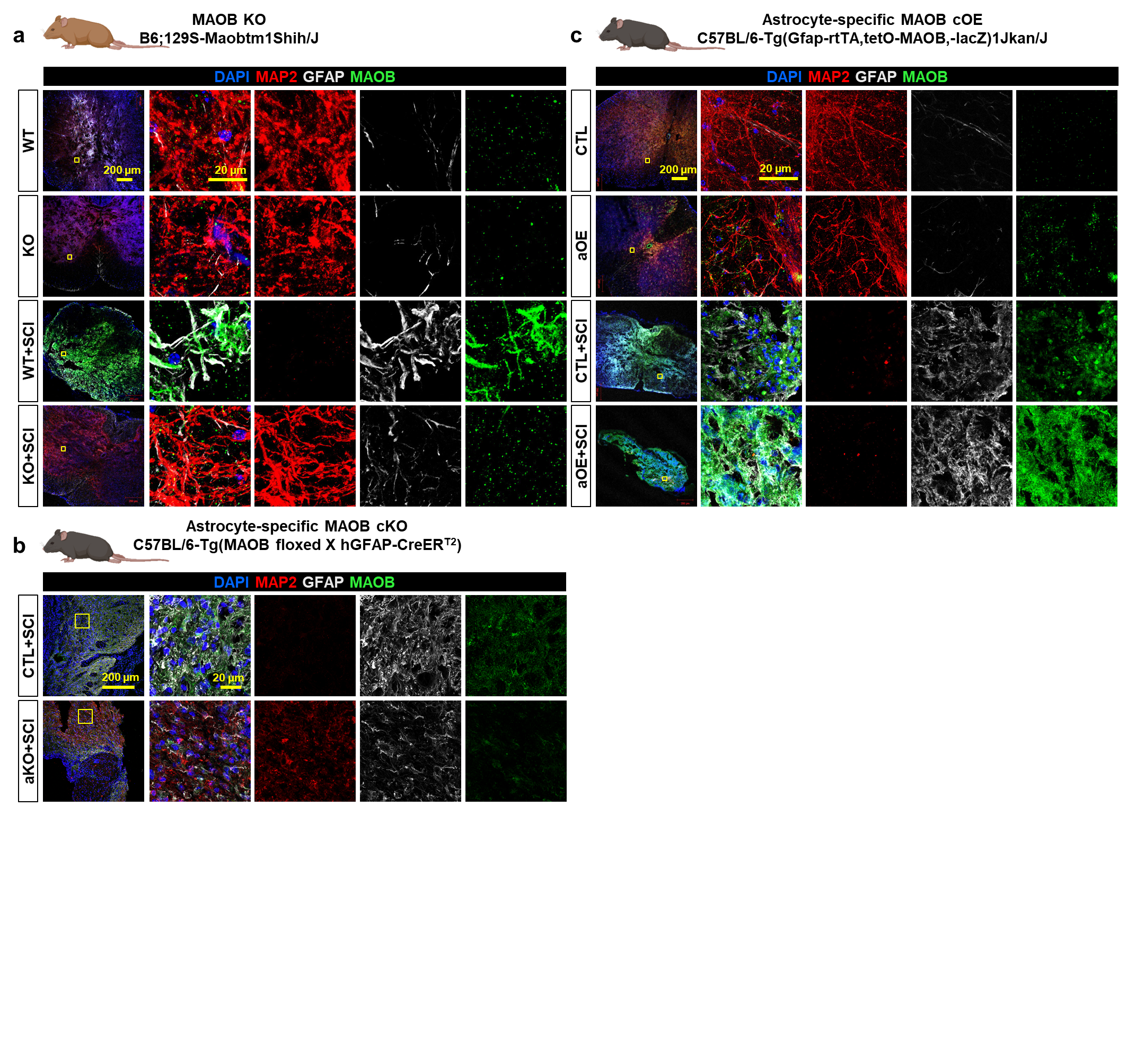
 Immunohistological analysis of astrocyte reactivity and neuronal quantity in MAOB KO, aKO, and aKO mice.**

**a-c** Confocal images of injured area showing individual channels for MAP2 (red), GFAP (white), and MAOB (green) at PI 10w in each group for MAOB KO (**a**), aOE (**b**), and aKO (**c**). Each yellow box in merged images indicates the magnified region of interest.

**Figures. S2.**

**
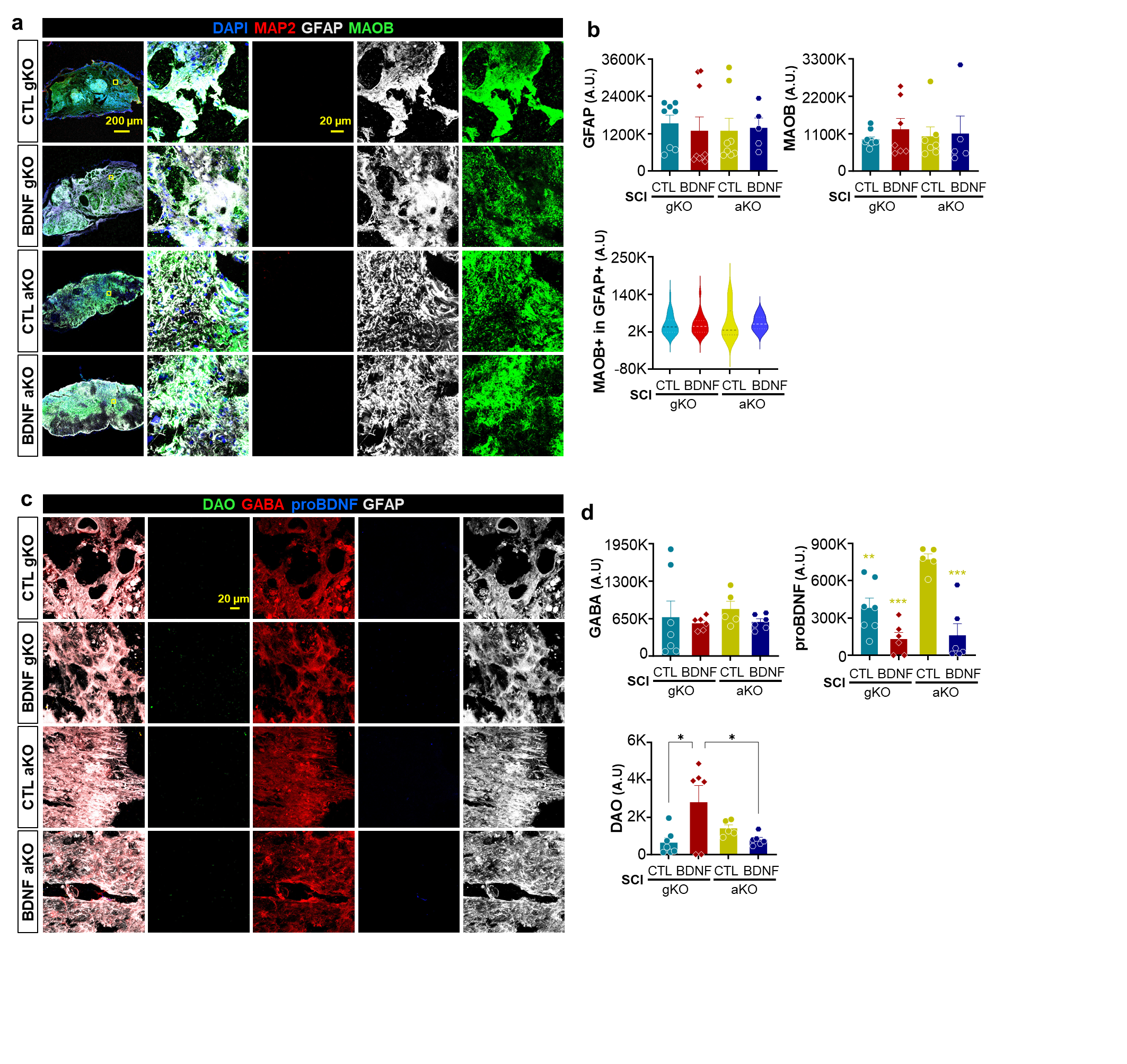
**

**BDNF signaling is downstream of astrocyte reactivity and astrocytic GABA expression.**

**a** Confocal images of the injured area in each group stained with anti-MAP2 (red), GFAP (white), MAOB (green) antibodies, and DAPI (blue) at PI 8w. Yellow box indicates the magnified region of interest. **b** The intensity of GFAP (left), MAOB (middle), and GFAP-positive MAOB (right) had no significant change among the groups. **c** Confocal images of the injured area in each group stained with anti-DAO (green), GABA (red), proBDNF (blue), and GFAP (white) antibodies at PI 8w. **d** (Left) GABA intensity showed no significant change among the groups. (Middle) Compared their respective CTL, both BDNF gKO and aKO showed a significant decrease in proBDNF intensity. (Bottom) BDNF gKO showed a significant increase in DAO intensity compared to CTL. All data are expressed as mean ± S.E.M. **P* < 0.05.

**Figures. S3.**

**
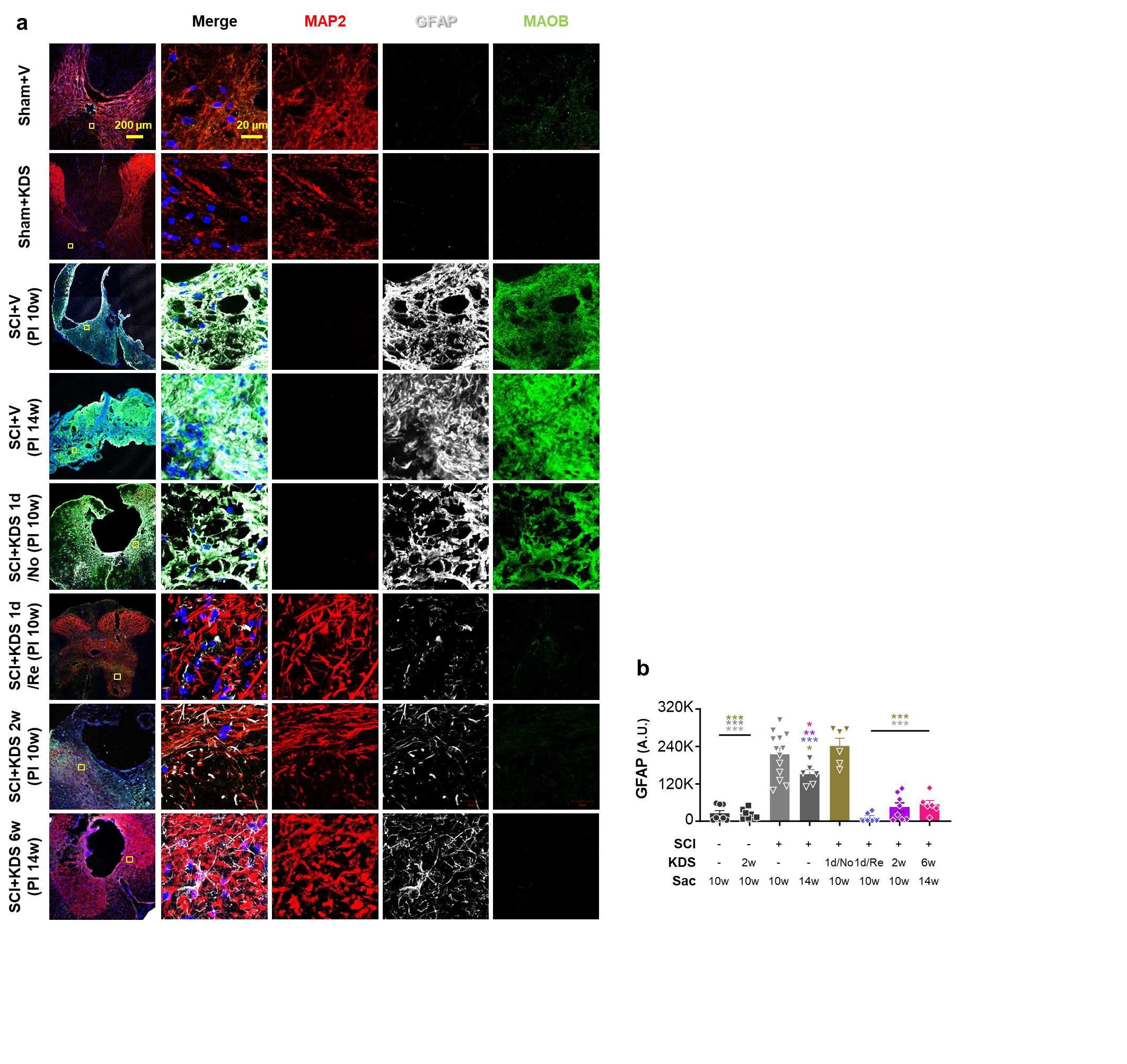
**

**Quantification of GFAP expression following KDS2010 treatment after SCI.**

**a** Representative confocal images showing GFAP immunoreactivity (white) in spinal cord sections from each group: Sham+V, SCI+V, SCI+KDS 1d/No, SCI+KDS 1d/Re, SCI+KDS 2w, and SCI+KDS 6w. Yellow squares indicate high-magnification inset areas. **b** Quantitative analysis of GFAP signal intensity within the lesion area. GFAP expression was markedly elevated in the SCI+V group compared to Sham+V, indicating robust reactive astrogliosis. KDS2010 treatment significantly reduced GFAP intensity in the SCI+KDS 1d/Re, SCI+KDS 2w, and SCI+KDS 6w groups, indicating attenuation of astrocyte reactivity. In contrast, SCI+KDS 1d/No exhibited persistently high GFAP levels, comparable to SCI+V, consistent with the lack of functional recovery in this group. All data are expressed as mean ± S.E.M. ****P* < 0.001.

**Figures. S4.**

**
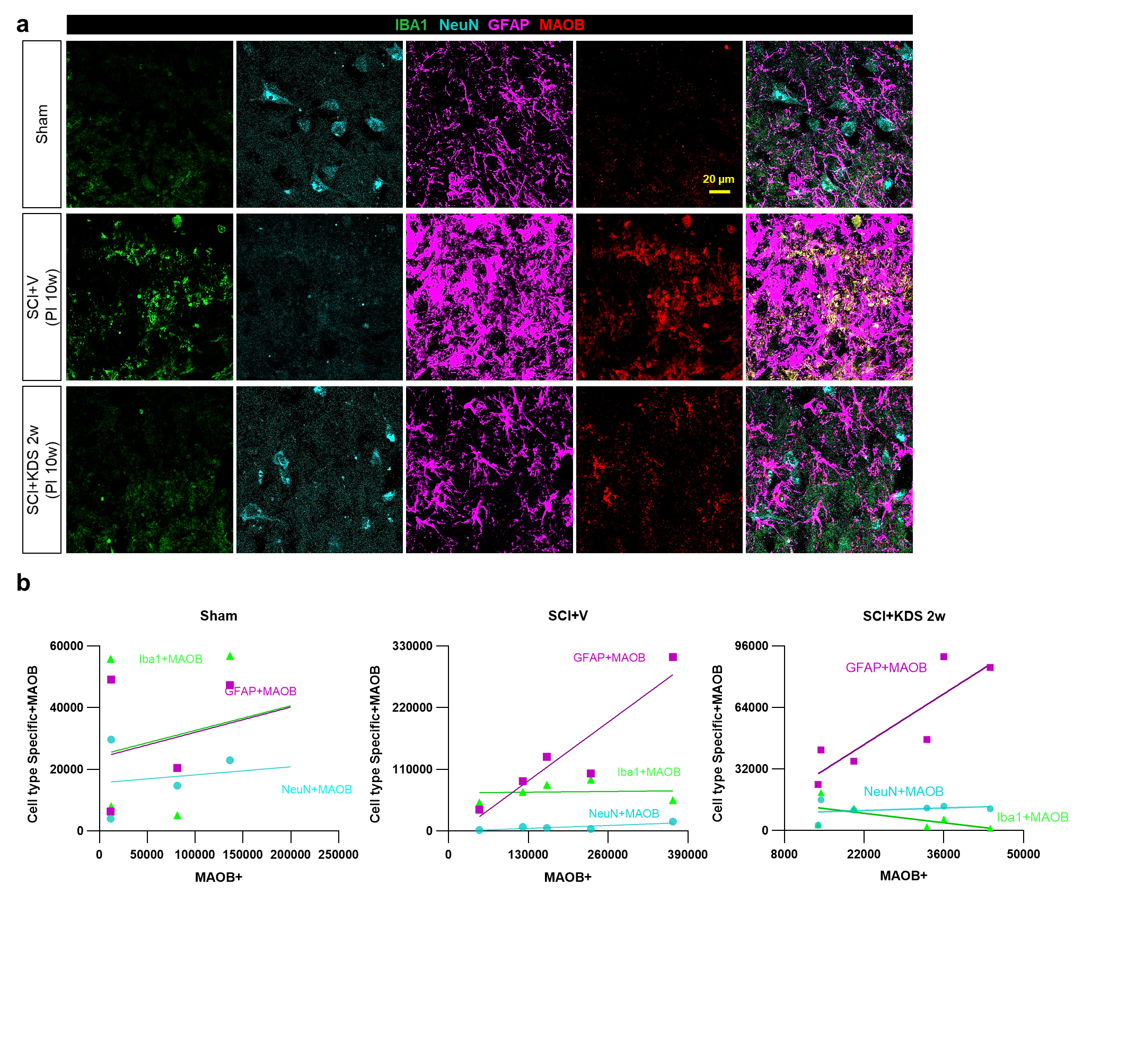
**

**Pearson’s correlation analysis between MAOB and cell-type specific markers.**

**a** Representative confocal images showing co-immunostaining of IBA1 (green), NeuN (cyan), GFAP (magenta), and MAOB (red) in spinal cord sections from Sham, SCI, and SCI+KDS 2w groups at PI 10w. Scale bar, 20 μm.
**b** Scatter plots illustrating Pearson’s correlation between total MAOB intensity (x-axis) and marker-specific co-localized MAOB intensity (y-axis) for GFAP (magenta squares), NeuN (cyan circles), and IBA1 (green triangles) in each group. GFAP–MAOB correlation was strongest across all conditions, with r = 0.355 in Sham, r = 0.925 in SCI, and r = 0.870 in SCI+KDS2010. By contrast, correlations for NeuN–MAOB and IBA1–MAOB were weak or inconsistent. These results suggest that MAOB expression after SCI is primarily associated with GFAP-positive astrocytes.

**Figures. S5.**

**
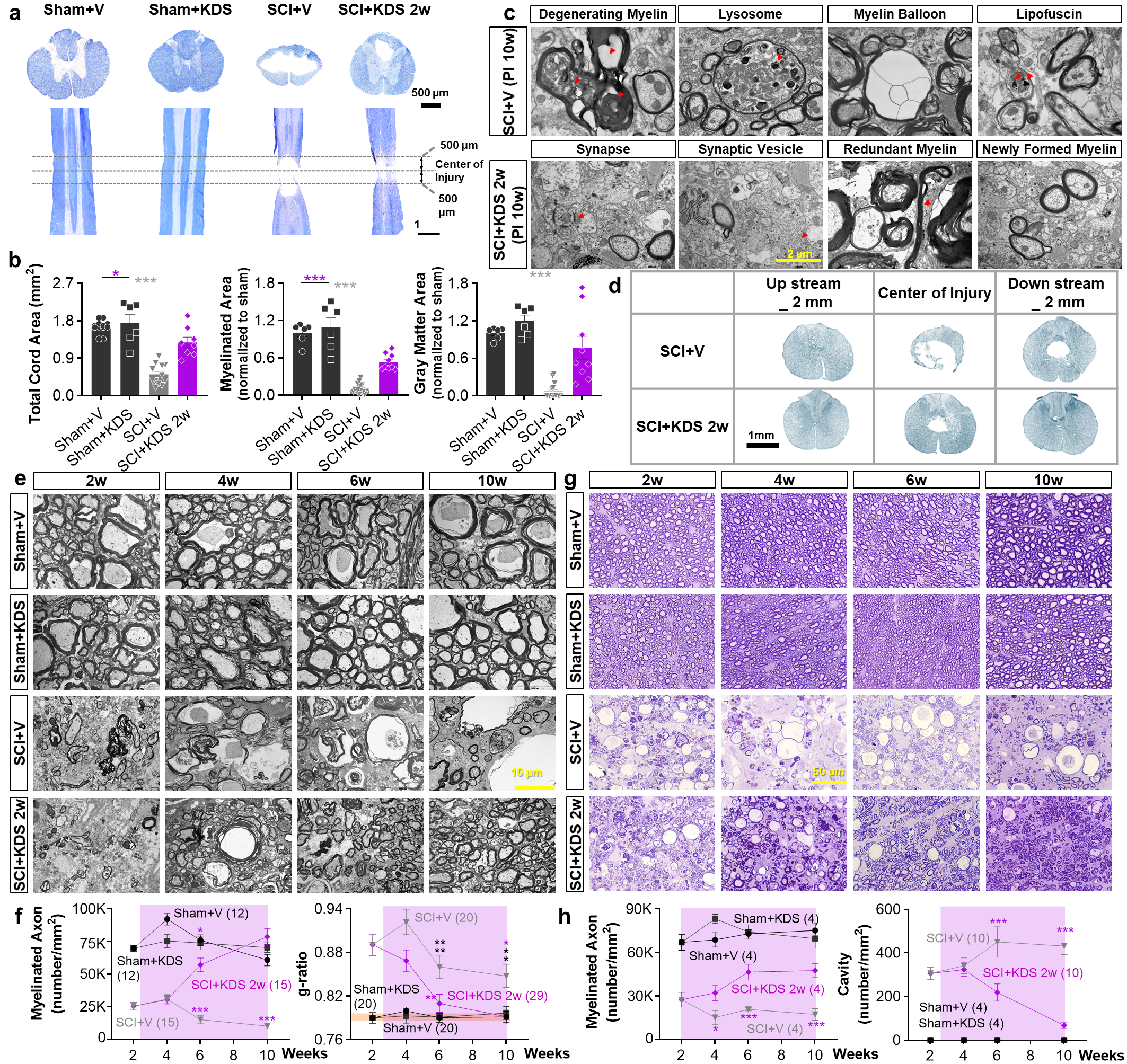
**

**Signs of remyelination through MAOB inhibition.**

**a** EC staining of cross (top) and longitudinal (bottom) sections of spinal cord tissues in each group (Sham+V, Sham+KDS, SCI+V, and SCI+KDS 2w) at PI 10w. **b** Compared to Sham+V and Sham+KDS, SCI+V showed significantly reduced total spinal cord, myelinated, and grey matter, along with an enlarged cavity size. All were significantly recovered in SCI+KDS 2w. **c** (Top) Representative TEM images showed evidence of myelin degeneration after SCI (SCI+V), such as degenerating myelin, lysosome, myelin balloon, and lipofuscin, at PI 10w. (Bottom) Representative TEM images showed evidence of neuroregeneration, such as synapse, synaptic vesicle, redundant myelin, and newly formed myelin, at PI 10w, when MAOB was inhibited after SCI (SCI+KDS 2w). **d** Luxol Fast Blue (LFB) staining showed recovery of gray-white matter boundaries and reduced cavity formation in the SCI+KDS 2w compared to SCI+V. **e** TEM images of spinal cord tissues in each group at PI 2, 4, 6, and 10w. **f** Compared to Sham+V and Sham+KDS, SCI+V showed severe loss of myelination (top) and increase of g-ratio (bottom). In contrast, SCI+KDS 2w showed a gradual increase in the number of small-sized myelinated axons (top) and a restoration of g-ratio to the optimal rage of 0.790 ± 0.005 (orange shade) at PI 10w. **g** TB staining of spinal cord tissues in each group at PI 2, 4, 6, and 10 week. **h** Compared to Sham+V and Sham+KDS, SCI+V showed severe loss of myelination (top) and the emergence of large cavities (bottom). In contrast, SCI+KDS 2w showed a gradual increase in the number of small-sized myelinated axons (top) and gradual decrease in the density of cavities (bottom). Purple shades in **f** and **h** indicate the duration of KDS2010 administration. All data are expressed as mean ± S.E.M. **P* < 0.05; ***P* < 0.01; ****P* < 0.001.

**Figures. S6.**

**
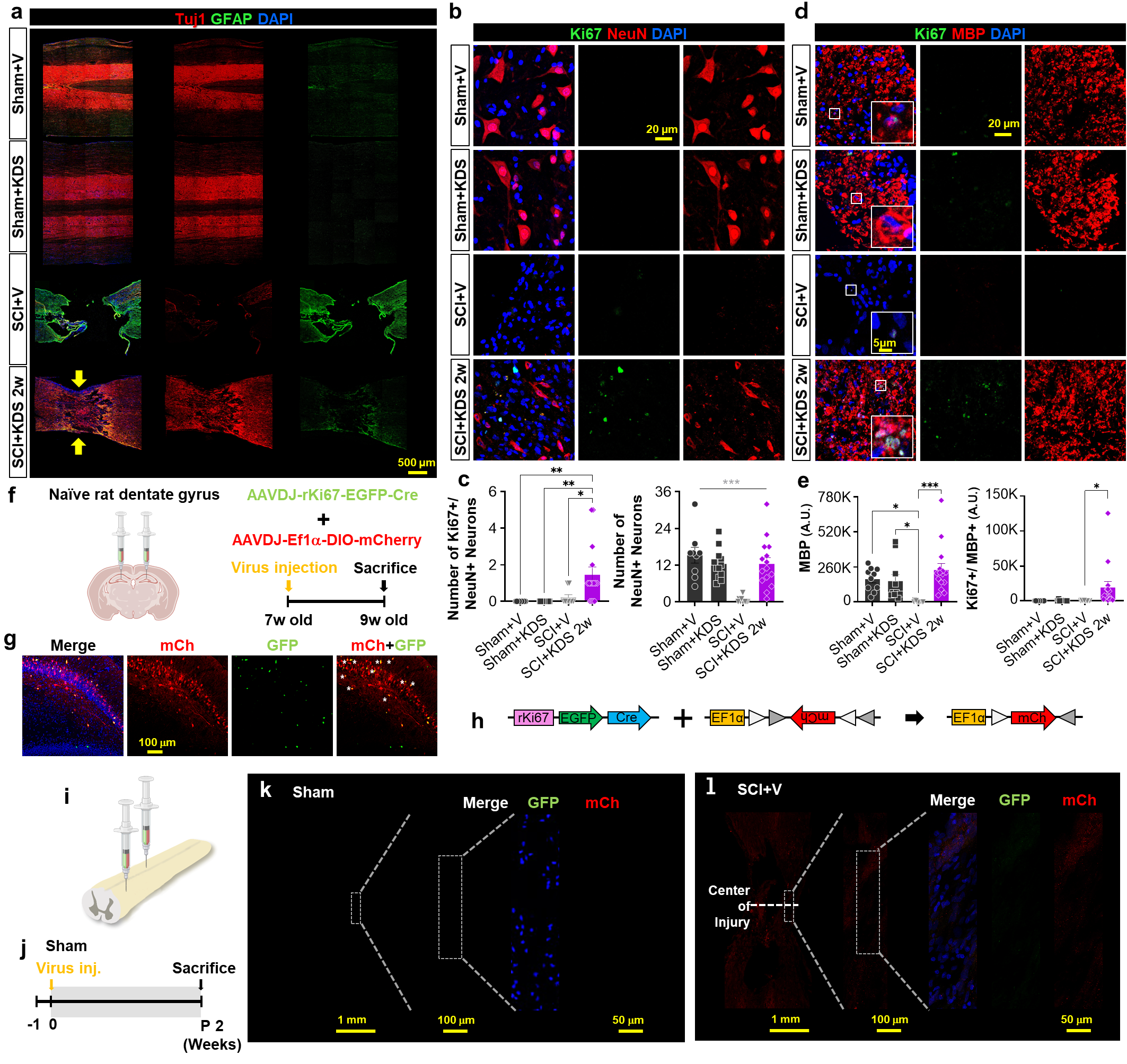
**

**Increased neuronal and oligodendrocyte proliferation and validation of Ki67 promoter-driven recombination in brain and spinal cord.**

**a** Confocal images of longitudinal sections of spinal cord tissues stained with anti-Tuj1 (red) and GFAP (green) antibodies at PI 10w in each group with sub-acute phase KDS2010 treatment (PI 2w). Yellow arrow indicates a cavity and atrophy in the center of the injured area. **b** Confocal images of the injured area in each group stained with anti-Ki67 (green), NeuN (red) antibodies, and DAPI (blue) at PI 10w. **c** (Top) Ki67-positive proliferating neurons were significantly increased in SCI+KDS 2w compared to Sham-operated groups and SCI+V. (Bottom) The number of NeuN-positive neurons in SCI+V was significantly reduced in SCI+V, while SCI+KDS 2w showed a significant recovery. **d** Confocal images of the injured area in each group stained with anti-Ki67 (green), MBP (red) antibodies, and DAPI (blue) at PI 10w. **e** (Top) The MBP intensity in SCI+V was significantly reduced compared to Sham-operated groups, while SCI+KDS 2w showed a significant recovery. (Bottom) Ki67-positive proliferating oligodendrocytes were significantly increased in SCI+KDS 2w compared to Sham-operated groups and SCI+V. **f** (Left) Schematics of virus injection strategy targeting the dentate gyrus of rat. (Right) Experimental timeline using 7-week-old rats involving the injection of a mixture of two viruses. **g** Confocal images of the dentate gyrus showing mCh and/or GFP-positive cells stained with anti-GFP (green) antibody and DAPI (blue) at 2 weeks after virus injection. The percentage of proliferating and proliferated cells was approximately 5.14±0.53% (n=3) for 2 weeks. Asterisks indicate both GFP- and mCh-positive cells. **h**. Schematic illustration of the DNA constructs used in each virus and the expected outcome of Cre-mediated recombination. **i**. Schematic diagram showing virus injection sites at 1 mm proximal and distal to the T9 spinal cord region in the Sham group. **j**. Experimental timeline of virus injection and observation in the Sham group. **k, l** Confocal images of the T9 spinal cord region in the Sham and SCI+V group, stained with anti-GFP (green), mCh (red), and DAPI (blue) 2 weeks after virus injection. No detectable GFP or mCherry signal was observed, indicating the absence of proliferative or recombined cells under non-injured conditions. Scale bar: 1 mm. All data are expressed as mean ± S.E.M. **P* < 0.05; ***P* < 0.01; ****P* < 0.001.

**Figures. S7.**

**
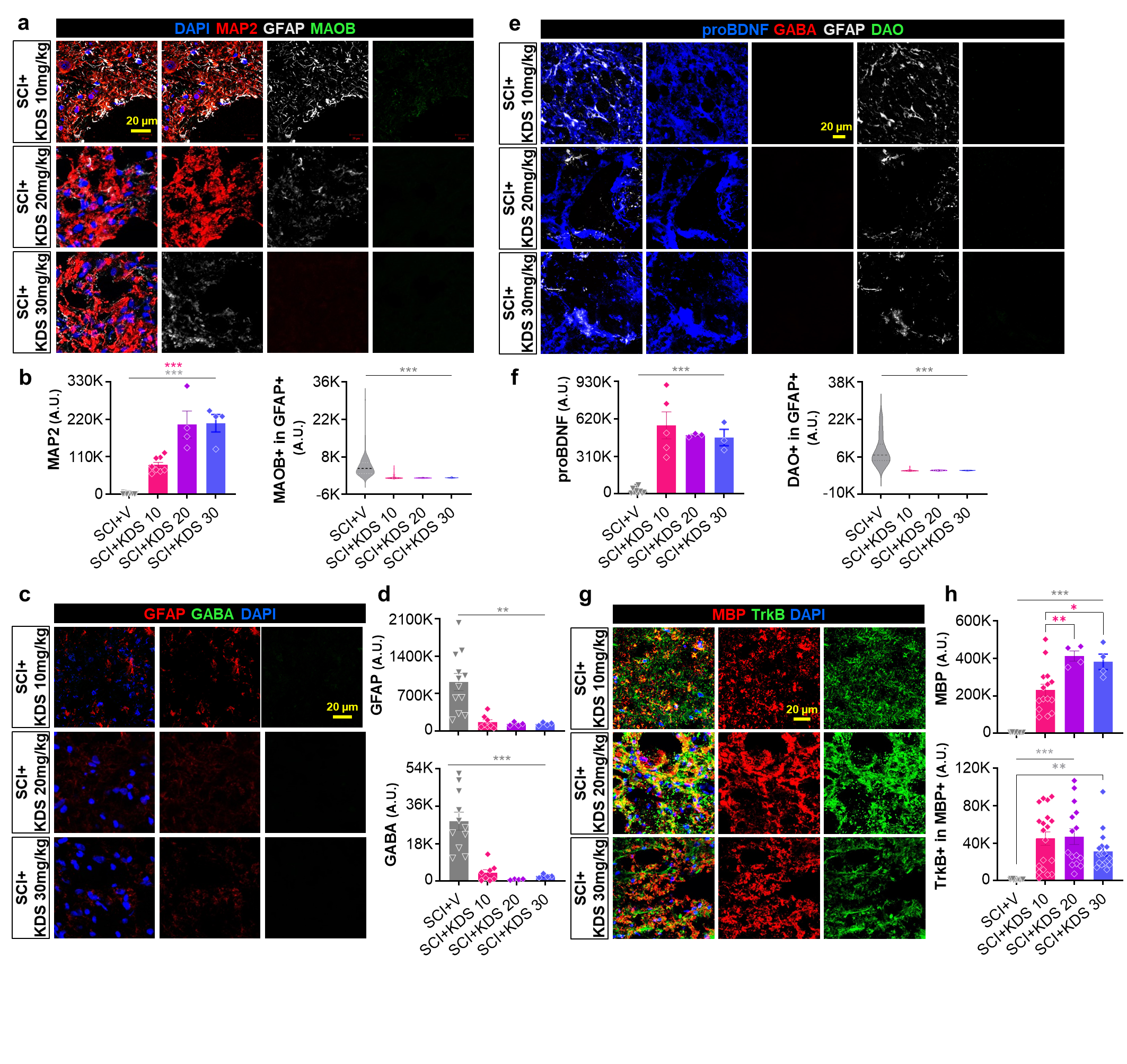
**

**KDS2010, at several concentrations, reduces astrocyte reactivity and GABA expression while enhancing proBDNF and TrkB expression.**

**a** Confocal images of the injured area in SCI+KDS 10mpk, 20mpk, and 30mpk stained with anti-MAP2 (red), GFAP (white), MAOB (green) antibodies, and DAPI (blue) at PI 10w. **b** (Left) The intensity of MAP2 showed a significant increase in SCI+KDS 10mpk, 20mpk, and 30mpk compared to SCI+V. All doses of the KDS induced a significant reduction in astrocytic MAOB expression (right) compared to SCI+V. **c** Confocal images of the injured area in SCI+KDS 10mpk, 20mpk, and 30mpk stained with anti-GFAP (red) and GABA (green) antibodies, and DAPI at PI 10w. **d** The intensities of GFAP (left) and GABA (right) in SCI+KDS 10mpk, 20mpk, and 30mpk were significantly reduced compared to SCI+V. **e** Confocal images of the injured area in SCI+KDS 10mpk, 20mpk, and 30mpk stained with anti-proBDNF (blue), GABA (red), GFAP (white), and DAO (green) antibodies at PI 10w. **f** The intensity of proBDNF significantly increased in SCI+KDS 10mpk, 20mpk, and 30mpk compared to SCI+V, while the intensity of astrocytic DAO significantly decreased. **g** Confocal images of the injured area in SCI+KDS 10mpk, 20mpk, and 30mpk stained with anti-MBP (red) and TrkB (green) antibodies, and DAPI (blue) at PI 10w. **h** (Left) The intensity of MBP showed a significant recovery in SCI+KDS 10mpk, 20mpk, and 30mpk compared to SCI+V. (Bottom) The intensity of MBP-positive TrkB showed a significant recovery in SCI+KDS 10mpk, 20mpk, and 30mpk compared to SCI+V. All data are expressed as mean ± S.E.M. **P* < 0.05; ***P* < 0.01; ****P* < 0.001.

**Figures. S8.**

**
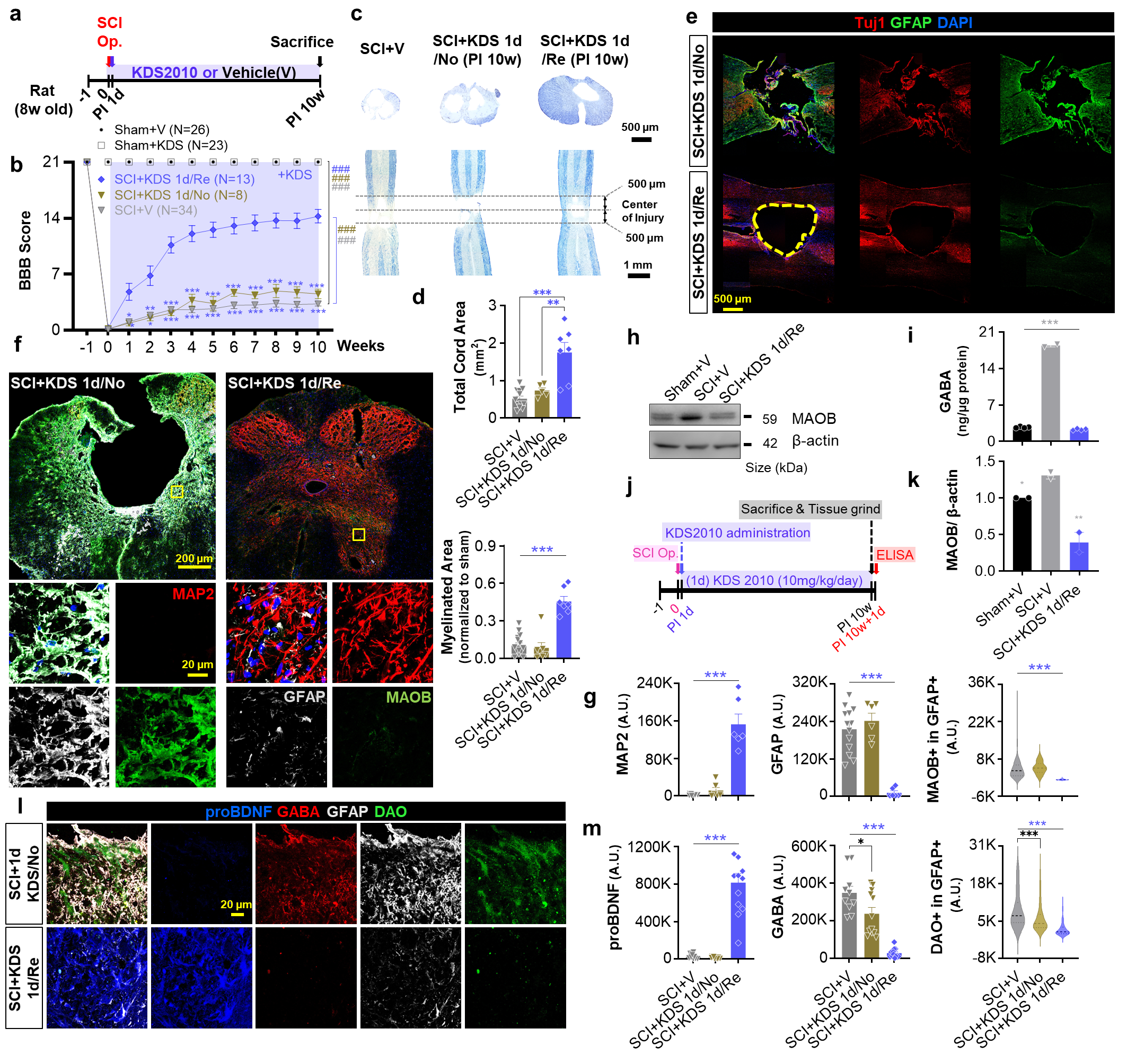
**

**MAOB inhibition from the acute phase induces recovery in some cases, while in others, recovery fails due to the compensatory action of DAO.**

**a** Experimental timelines using 8-week-old rats with the SCI operation and treatment with KDS2010, from 1 day after (acute) SCI. **b** SCI+KDS 1d/Re exhibited a stable gait at PI 10w, whereas SCI+KDS1d/No did not show any behavioral recovery in BBB locomotor test. Purple shade indicates the duration of KDS2010 administration. **c** EC staining of cross (top) and longitudinal (bottom) sections of spinal cord tissues in each group (SCI+V, SCI+KDS 1d/No, and SCI+KDS 1d/Re) at PI 10w. Dashed lines indicate region of analysis. **d** Reduced total spinal cord and myelinated areas in SCI+V and SCI+KDS 1d/No were all significantly recovered in SCI+KDS 1d/Re at PI 10w. **e** Confocal images of longitudinal sections of spinal cord tissues stained with anti-Tuj1 (red), GFAP (green) antibodies, and DAPI (blue) at PI 10w in each group. **f** Confocal images of cross sections of spinal cord tissues in SCI+KDS 1d/No and SCI+KDS 1d/Re stained with anti-MAP2 (red), GFAP (white), MAOB (green) antibodies, and DAPI (blue) at PI 10w. **g** (Top) The intensity of MAP2 showed a significant increase in SCI+KDS 1d/Re compared to SCI+V and SCI+KDS 1d/No. In contrast, astrocytic GFAP (middle) and MAOB expression (bottom) significantly decreased in SCI+KDS 1d/Re compared to SCI+V and SCI+KDS 1d/No. **h** Western blotting of MAOB in Sham+V, SCI+V, and SCI+KDS 1d/Re at PI 10w. β-actin was used as a control for protein amount. **i** The MAOB protein level was significantly reduced in SCI+KDS 1d/Re compared to SCI+V. **j** Experimental timeline for ELISA using rats with SCI operation and acute phase KDS2010 treatment. **k** The concentration of GABA in Sham+V, SCI+V, and SCI+KDS 1d/Re at PI 10w. **l** Confocal images of the injured area in SCI+KDS 1d/No and SCI+KDS 1d/Re stained with anti- proBDNF (blue), GABA (red), GFAP (white), and DAO (green) antibodies. **m** (Left) The intensity of proBDNF was significantly increased in SCI+KDS 1d/Re compared to SCI+V and SCI+KDS 1d/No. The intensity of GABA (middle) and astrocytic DAO (right) were significantly reduced in SCI+KDS 1d/Re, while a significant level remained in SCI+V and SCI+KDS 1d/No. All data are expressed as mean ± S.E.M. **P* < 0.05; ***P* < 0.01; ****P* < 0.001.

**Figures. S9.**

**
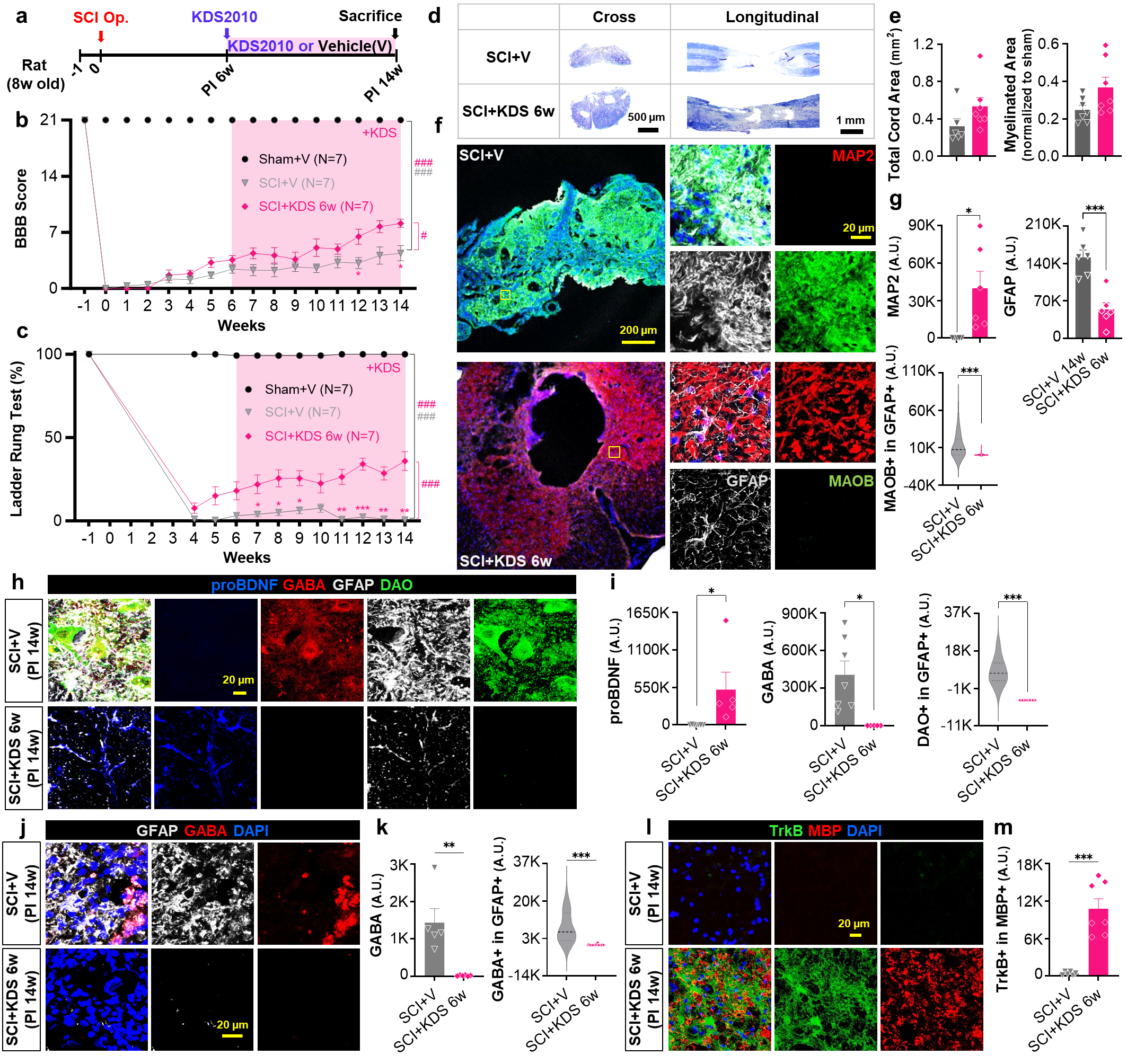
**

**MAOB inhibition from the chronic phase promotes recovery after SCI.**

**a** Experimental timelines using 8-week-old rats with the SCI operation and treatment with KDS2010, from 6 weeks after (chronic) SCI. **b, c** SCI+KDS 6w showed a significant motor recovery in BBB locomotor test (**b**) and ladder rung test (**c**). Pink shades indicate the duration of KDS2010 administration. **d** EC staining of cross (top) and longitudinal (bottom) sections of spinal cord tissues in SCI+V and SCI+KDS 6w at PI 14w. **e** The total spinal cord and myelinated areas in SCI+KDS 6w showed an increasing trend compared to SCI+V. **f** Confocal images of cross sections of spinal cord tissues in SCI+V and SCI+KDS 6w stained with anti-MAP2 (red), GFAP (white), MAOB (green) antibodies, and DAPI (blue) at PI 14w. **g** (Top) The intensity of MAP2 showed a significant increase in SCI+KDS 6w compared to SCI+V. In contrast, astrocytic GFAP (middle) and MAOB expression (bottom) significantly decreased in SCI+KDS 6w compared to SCI+V. **h** Confocal images of the injured area in SCI+V and SCI+KDS 6w stained with anti- proBDNF (blue), GABA (red), GFAP (white), and DAO (green) antibodies. **i** (Left) The intensity of proBDNF was significantly increased in SCI+KDS 6w compared to SCI+V. The intensity of GABA (middle) and astrocytic DAO (right) were significantly reduced in SCI+KDS 6w, while a significant level remained in SCI+V and SCI+KDS 1d/No. **j** Confocal images of the injured area in SCI+V and SCI+KDS 6w stained with anti-GFAP (white), GABA (red) antibodies, and DAPI (blue) at PI 14w. **k** The elevated levels of astrocytic GFAP and GABA in SCI+V were significantly reduced in SCI+KDS 6w. **l** Confocal images of the injured area in SCI+V and SCI+KDS 6w stained with anti-TrkB (green) and MBP (red) antibodies, and DAPI (blue) at PI 14w. **m** The intensity of MBP-positive TrkB (bottom) was significantly increased in SCI+KDS 6w compared to SCI+V. All data are expressed as mean ± S.E.M. **P* < 0.05; ***P* < 0.01; ****P* < 0.001.

**Figures. S10.**

**
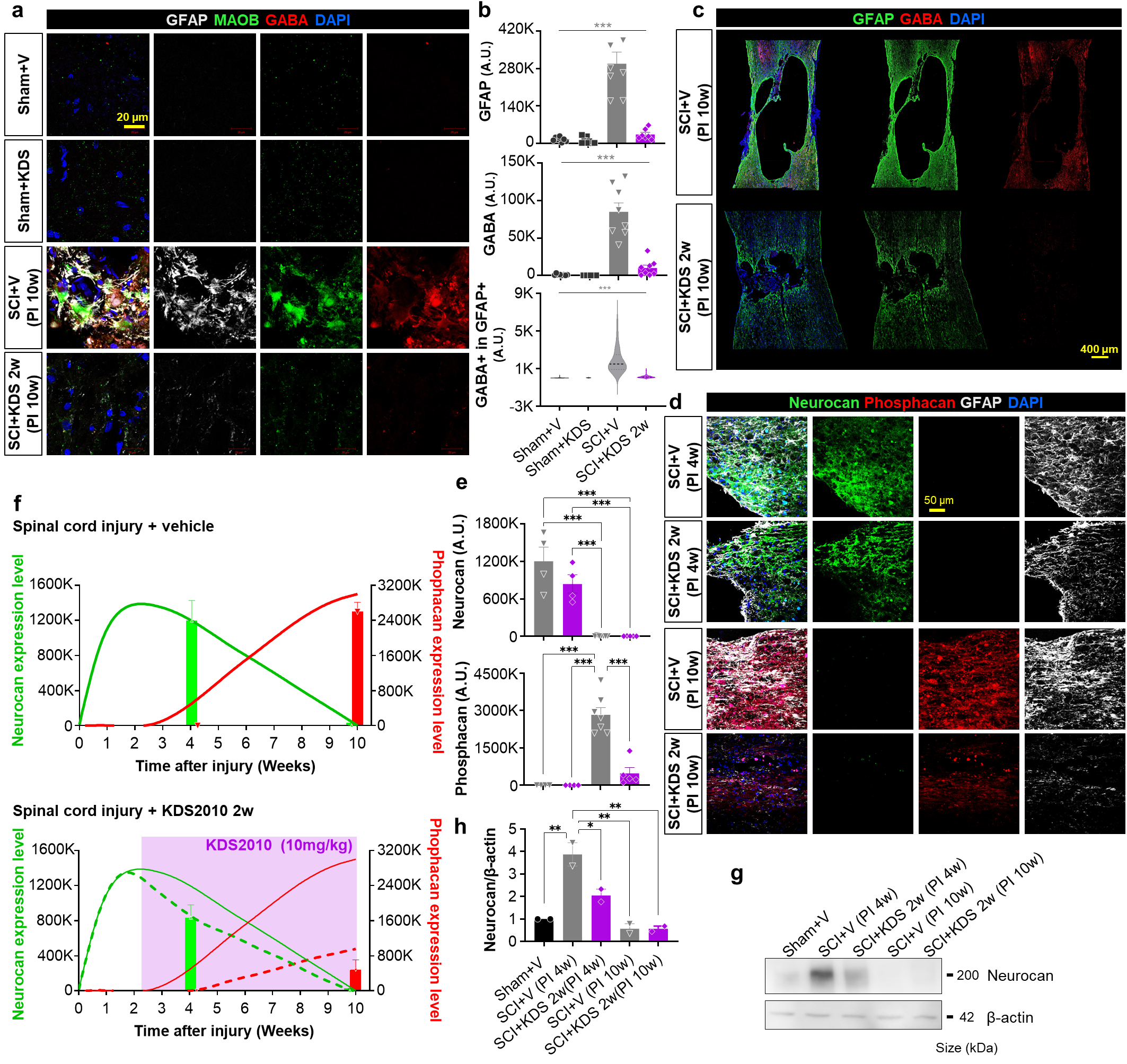
**

**MAOB inhibition reduces astrocytic GABA expression and CSPGs.**

**a** Confocal images of the injured areas in each group stained with anti-GFAP (white), MAOB (green), GABA (red) antibodies, and DAPI (blue) at PI 10w. **b** The intensity of GABA and astrocytic GABA, along with GFAP, significantly increased in SCI+V compared to Sham+V or Sham+KDS, while they significantly recovered in SCI+KDS 2w. **c** Confocal images of longitudinal sections of spinal cord tissues in SCI+V and SCI+KDS 2w stained with anti-GFAP (green), GABA (red) antibodies, and DAPI (blue) at PI 10w. **d** Confocal images of injured areas stained with anti-Neurocan (green), anti-Phosphacan (red), anti-GFAP (white), and DAPI at PI 4 and 10 weeks. **e** Quantification of Neurocan and Phosphacan intensities in each group. **f** Bar graph showing Neurocan and Phosphacan intensities over time, with dashed lines indicating expression trends in SCI+KDS 2w. **g** Western blot of Neurocan expression in Sham+V, SCI+V (PI 4w, 10w), and SCI+KDS 2w (PI 4w, 10w), normalized to β-actin. **h** Normalized Neurocan intensity relative to β-actin in each group. All data are expressed as mean ± S.E.M. **P* < 0.05; ***P* < 0.01; ****P* < 0.001.

**Figures. S11.**

**
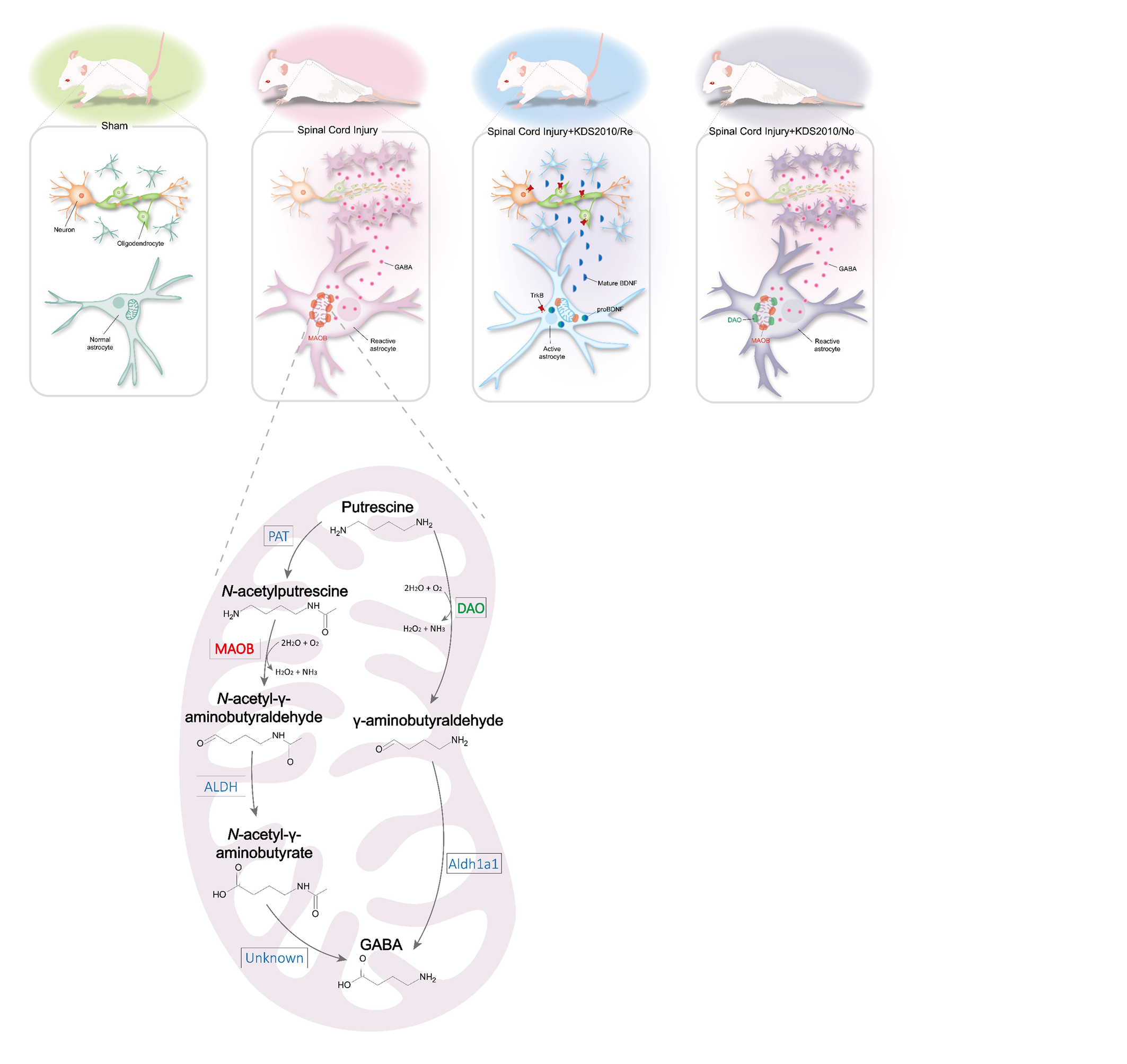
**

**Schematic model for molecular mechanism of neuroregeneration, remyelination, and functional recovery after SCI through MAOB inhibition.**

First, sham group possesses functional neurons, oligodendrocytes, and normal astrocytes. Second, SCI group shows reactive astrocytes with aberrant MAOB-dependent GABA production and release which impede neuroregeneration, remyelination, and functional recovery after SCI. Third, MAOB inhibition by KDS2010 leads to emergence of active astrocytes with increase of proBDNF and TrkB, and reduction of GABA. Finally, mBDNF, which is generated from cleavage of proBDNF, could cause neuroregeneration, remyelination, and functional recovery by acting on neuronal or oligodendrocytic TrkB which is also increased by MAOB inhibition in SCI+KDS2010/Re. Fourth, even if KDS2010 treated in acute phase, alternative pathway of DAO activated to degrade putrescine to GABA in reactive astrocytes, which consequently exerts a brake on proBDNF, neuroregeneration and functional recovery after SCI in SCI+KDS2010/No.

**Table S1. Weekly Changes in Body Weight (g) and Period of Medicated Drinking Water Administration Across Experimental Groups.**

|  | 0w | 1w | 2w | 3w | 4s | 5s | 6s | 7w | 8w | 9w | 10w | 11w | 12w | 13w |
| --- | --- | --- | --- | --- | --- | --- | --- | --- | --- | --- | --- | --- | --- | --- |
| d.w | weight*0.09 | weight*0.15 | weight*0.11 | weight*0.10 | weight*0.10 | weight*0.09 | weight*0.09 | weight*0.075 | weight*0.075 | weight*0.08 | weight*0.08 | weight*0.08 | weight*0.08 | weight*0.08 |
| Sham | 0.00 | 43.13 | 45.00 | 42.50 | 28.75 | 28.75 | 20.63 | 15.63 | 17.50 | 10.63 |  |  |  |  |
| SCI | 0.00 | 32.34 | 36.09 | 28.59 | 25.16 | 18.91 | 15.31 | 18.71 | 13.87 | 10.00 | 13.82 | 12.35 | 5.59 | 8.53 |
| Sham/KDS 1d | 0.00 | 26.67 | 3.33 | 30.00 | 50.00 | 25.00 | 21.67 | 18.33 | 8.33 | 23.33 |  |  |  |  |
| Sham/KDS 2w | 0.00 | 33.00 | 17.00 | 32.00 | 31.00 | 21.00 | 8.00 | 25.00 | 10.00 | 10.00 |  |  |  |  |
| SCI/KDS 1d | 0.00 | -44.55 | 21.82 | 23.03 | 50.91 | 28.03 | 16.82 | 19.85 | 14.70 | 7.88 |  |  |  |  |
| SCI/KDS 2w | 0.00 | 47.67 | 16.00 | 33.67 | 31.00 | 31.33 | 16.67 | 18.33 | 11.67 | 7.17 | 14.50 | 8.00 | 1.50 | 7.50 |
| SCI/KDS 6w | 0.00 | 24.00 | 27.50 | 36.00 | 31.50 | 14.50 | 13.00 | 17.50 | 7.00 | 13.00 | 11.00 | 8.00 | 7.00 | 8.00 |
| SCI/KDS 2w 20mpk | 0.00 | 29.33 | 13.61 | 54.00 | 16.28 | 31.61 | 12.56 | 24.17 | 13.00 | 10.89 |  |  |  |  |
| SCI/KDS 2w 30mpk | 0.00 | 34.43 | 10.86 | 39.90 | 17.36 | 22.40 | 10.84 | 19.91 | 17.56 | 3.43 |  |  |  |  |

*All numerical data represent weekly changes in body weight (g).

*The orange shading in the table indicates the period during which the drug was administered.

**Table S2. Pharmacokinetic parameters of KDS2010 in pre-clinical study.**

|  | **AUC_inf_ (ng·h/mL)** | **CL/F (L/h/kg)** | **Vd/F(L/kg)** | **t_1/2_ (h)** |
| --- | --- | --- | --- | --- |
| **Mouse** | | | | |
| APP/PS1 mouse, 1 mg/kg single oral administration | 1132.3 ± 173.1 | 0.897 ± 0.135 | 6.127 ± 0.767 | 4.76 ± 0.43 |
| ICR mouse, 5 mg/kg single intravenous administration | 3592.8 ± 71.7 | 1.708 ± 0.082 | 10.439 ± 0.682 | 4.20 ± 0.20 |
| ICR mouse, 5 mg/kg single oral administration | 4175.7 ± 100.4 | 1.081 ± 0.035 | 6.601 ± 0.380 | 4.20 ± 0.10 |
| APP/PS1 mouse, 10 mg/kg single oral administration | 12898.7 ± 740.5 | 0.858 ± 0.003 | 7.014 ± 0.934 | 5.70 ± 0.70 |
| ICR mouse, 10 mg/kg single oral administration | 7918.4 ± 457.2 | 1.318 ± 0.148 | 8.409 ± 0.843 | 4.40 ± 0.20 |
| ICR mouse, 30 mg/kg single oral administration | 27111.3 ± 1089.2 | 0.872 ± 0.014 | 5.450 ± 0.512 | 4.30 ± 0.40 |
| **Rat** | | | | |
| SD Rat, 1 mg/kg single intravenous administration | 164.2 ± 13.1 | 6.122 ± 0.522 | 18.377 ± 2.743 | 2.10 ± 0.30 |
| SD Rat, 9 mg/kg single oral administration | 9123.6 ± 4131.2 | 1.140 ± 0.456 | 19.735 ± 4.340 | 14.60 ± 8.70 |
| **Monkey** | | | | |
| Cynomolgus monkey, 5 mg/kg single intravenous administration | 17868.6 ± 2455.5 | 0.299 ± 0.046 | 3.714 ± 0.609 | 8.65 ± 0.97 |
| Cynomolgus monkey, 10 mg/kg single oral administration | 43623.4 ± 1286.9 | 0.228 ± 0.039 | 4.488 ± 0.610 | 13.71 ± 0.48 |
| Cynomolgus monkey, 30 mg/kg single oral administration | 201786.9 ± 76769.5 | 0.155 ± 0.042 | 6.274 ± 1.666 | - 1. 16.95 |

*All data are presented as the mean ± standard deviation.

**Table S3. Demographic data in phase 1 clinical trial**

*All data are presented as the mean ± standard deviation, unless otherwise specified.

**Table S4. Pharmacokinetic parameters of KDS2010 in phase 1 clinical trial.**

*All data are presented as the mean ± standard deviation, except T_max_ (T_max,ss_) which is presented as median (minimum – maximum). C_max_, maximum concentration; T_max_, time to reach the C_max_; AUC_inf_, area under the concentration-time curve from 0 to infinity; AUC_τ,ss_, area under the concentration-time curve during the dosing intervals at steady state; t_1/2_, terminal half-life; CL/F, the apparent clearance; CL_R_, renal clearance. *Accumulation ratio was calculated as the ratio of AUC_τ,ss_ after the administration of the seventh dose in comparison with AUC_τ_ after the administration of the first dose. **Metabolic ratio was calculated as the ratio of AUC_last,parent_ in comparison with AUC_last,metabolite_.

**Movie S1. BBB score assessment of SCI+V and SCI+KDS 2w.**

(Above) SCI+V showed impaired motor function at PI 10w. (Below) SCI+KDS 2w showed a significant recovery of motor function at PI 10w.

**Movie S2. Ladder rung test of SCI+V and SCI+KDS 2w.**

(Above) SCI+V exhibited severe motor dysfunction with hindlimb slipping during traversing the ladder at PI 10w. (Below) SCI+KDS 2w exhibited a significant recovery at PI 10w.
