# Supplementary figures and images for: Astrocytic monoamine oxidase B (MAOB)–gamma-aminobutyric acid (GABA) axis as a molecular brake on repair following spinal cord injury
Source: Signal Transduct Target Ther. 2025 Sep 11;10:295. doi: 10.1038/s41392-025-02398-2 (PMC12423301; doi:10.1038/s41392-025-02398-2)

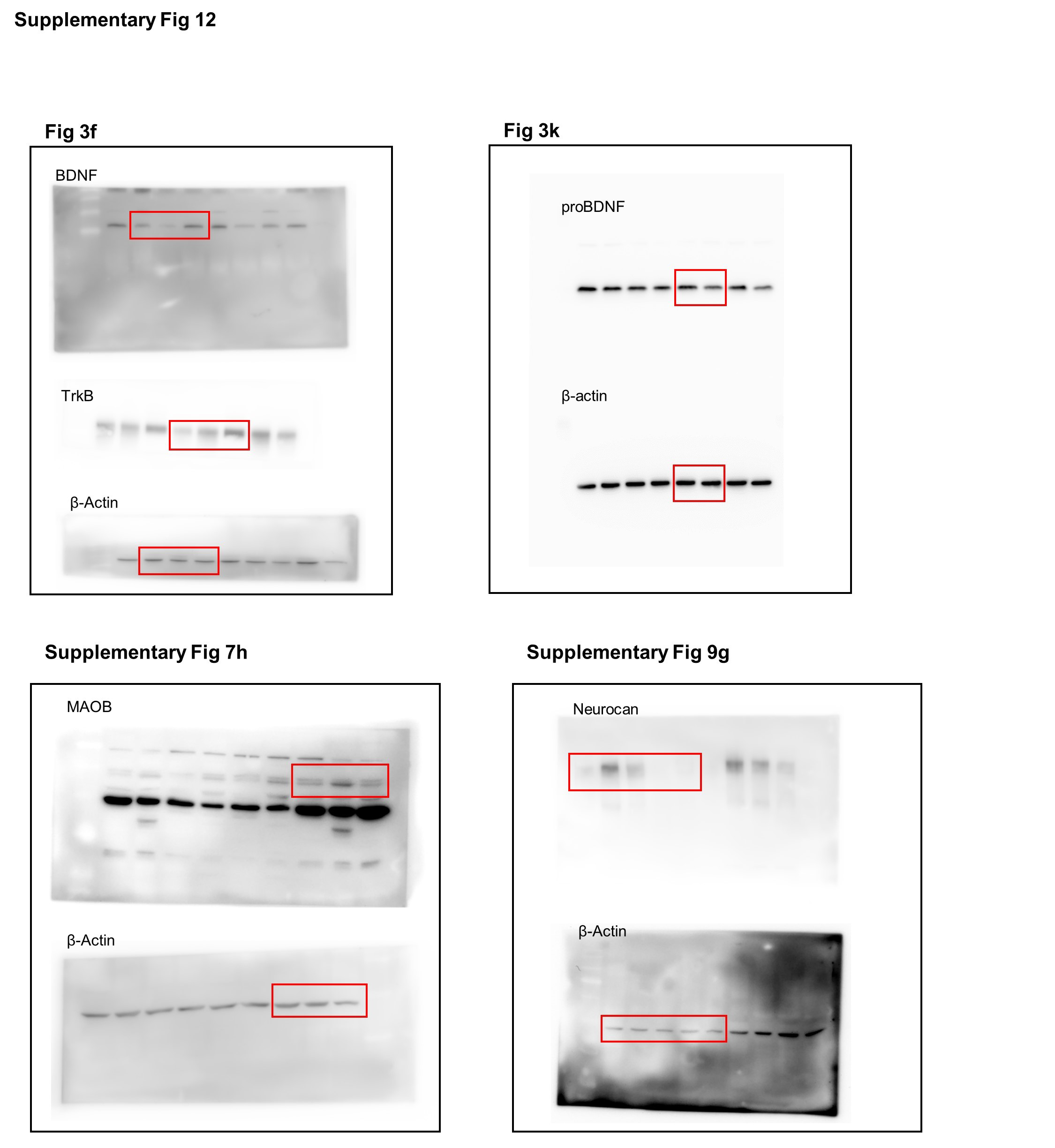

Supplement: Supplementary file 4 — uncropped western botting film [file 41392_2025_2398_MOESM4_ESM.jpg]
