# Supplementary material for: Astrocytic monoamine oxidase B (MAOB)–gamma-aminobutyric acid (GABA) axis as a molecular brake on repair following spinal cord injury
Source: Signal Transduct Target Ther. 2025 Sep 11;10:295. doi: 10.1038/s41392-025-02398-2 (PMC12423301; doi:10.1038/s41392-025-02398-2)
Supplement: Supplementary file 5 — Clinical study protocol [file 41392_2025_2398_MOESM5_ESM.pdf]

Clinical Study Protocol

A dose blocked-randomized, double-blind, placebo-controlled, single and multiple dosing, dose-escalation phase I clinical trial to evaluate the safety, tolerability, pharmacokinetics/pharmacodynamics of KDS2010 and food effect of KDS2010 on bioavailability after oral administration of KDS2010 in healthy young and elderly subjects

**Investigational Product:**	KDS2010
**Protocol No.:**	NB_KDS_ADP1_21
**Protocol Version:**	1.8
**Phase of Study:**	Phase I
**Principal Investigator:**	Department of Clinical Pharmacology and Therapeutics, Seoul National University College of Medicine and Hospital<br>SeungHwan Lee, MD, PhD
**Sponsor:**	NeuroBiogen Co., Ltd.
**Study Center:**	Clinical Trials Center, Seoul national University Hospital, 101 Daehak-ro, Jongno-gu, Seoul, 03080, South Korea
**Protocol Date:**	2023.02.06

CONFIDENTIAL

Any and all information contained in this document is the exclusive property of NeuroBiogen Co., Ltd. and is for the head of clinical trial institution, principal investigator, sub-investigator, clinical trial pharmacist, related administrative agencies, and Institutional review board (IRB) of the clinical trial institution. This protocol can only be used for the purpose of performing or evaluating a clinical trial, and if you want to use it for any other purpose, the written consent of NeuroBiogen is required, and the confidentiality of the document must be maintained.

SIGNATURE PAGE

A dose blocked-randomized, double-blind, placebo-controlled, single and multiple dosing, dose-escalation phase I clinical trial to evaluate the safety, tolerability, pharmacokinetics/pharmacodynamics of KDS2010 and food effect of KDS2010 on bioavailability after oral administration of KDS2010 in healthy young and elderly subjects

Protocol Writer: Ji Yun Park

Signature: ______________________ ______________________
Date

Principal Investigator: SeungHwan Lee MD, PhD

Signature: ______________________ ______________________
Date

❖ History of Clinical Study Protocol Revision

Version No.	Effective Date (YYYY/MM/DD)	Category	Summary of Change
1.0	2021/11/26	Enactment	NA
1.1	2022/02/11	1) 5.2.1. Selection of Dose<br>2) 9.2.2. Exclusion Criteria<br>3) 10.5. Dose Escalation to the Next Dose Level<br>4) 10.4.4. Prohibited Concomitant Medications and Foods<br>5) 10.7.2. Admission and Pharmacokinetic Evaluation Period<br>6) 12.2.1. Pharmacokinetic Endpoints	1) Addition of rationale for selection of dose<br>2) Elimination of concomitant medications in elderly subjects<br>3) Addition of SRC review before dose escalation<br>4) Addition of Contraindicated Drugs<br>5) Addition of the time point for measuring vital signs in case of multiple administration, addition of time window at the time of execution<br>6) Addition of specific metabolite analysis plan<br>7) Deletion of contents related to exploratory drug genomic analysis<br>8) Error correction
1.2	2022/02/17	3) 10.5. Dose Escalation to the Next Dose Level	Corrected details regarding the SRC review process when escalating the dose
1.3	2022/06/20	1) Name and Title of Principal Investigator and Co-investigator<br>2) Inclusion/Exclusion Criteria<br>3) Study Plan<br>4) Study Flow Chart<br>5) Name and Title of Principal Investigator and Co-investigator<br>6) Packing and Labeling of Investigational Drugs<br>7) Blinding<br>8) Randomization Number<br>9) Dose Escalation to the Next Dose Level<br>10) Blood Samplings/Urine collection, Sample Storage and Analysis Methods for PK Analysis<br>11) Admission and Pharmacokinetic Evaluation Period<br>12) Informed Consent Procedures<br>13) References<br>14) Appendices	Corrected details on elderly, women, Caucasian

1.4	2022/07/06	1) Inclusion/Exclusion Criteria<br>2) Observations & Assessments	Error correction
1.5	2022/07/11	1) Inclusion/Exclusion Criteria<br>2) Dose Escalation Criteria	Detailed description<br>Error correction
1.6	2022/07/27	1) Single dose Food effect<br>2) Observations & Assessments<br>3) Actions Taken in relation to Adverse Events	Error correction<br>Detailed description<br>Detailed description
1.7	2022/08/23	1) 5.2.2. Duration of Dosing and Timepoints of Blood samplings/Urine collections<br>2) 10.9.1.Blood Samplings/Urine collection, Sample Storage and Analysis Methods for PK Analysis	Error correction
1.8	2023/02/06	1) 4.3. PK/PD Sample Analysis Laboratory<br>2) 5.2.2. Duration of Dosing and Timepoints of Blood samplings/Urine collections	Addition of sample analysis laboratory (PD)<br><br>Detailed description

◈ Abbreviations and definition of terms

ADR	Adverse Drug Reaction,
AE	Adverse Event
ALTa	Alanine transaminase (aminotransferase)
aPTT	Activated Partial Thromboplastin Time
AST	Aspartate Transaminase (aminotransferase)
AUC_{inf}	Area under the concentration vs. time curve from time 0 extrapolated to infinite time after a single dose administration
AUC_{last}	Area under the concentration vs. time curve from the first observed to last measurable concentration
AUC_{tau}	Area under the Plasma Concentration-time Curve
BMI	Body Mass Index
BUN	Blood Urea Nitrogen
$C_{av,ss}$	Average Steady-state Plasma Drug Concentration
CL/F	Apparent Total Clearance
CL_{ss}/F	Apparent Total Clearance at Steady State
C_{max}	Maximum (Peak) Plasma Drug Concentration
$C_{max,ss}$	Maximum (Peak) Steady-state Plasma Drug Concentration
C_{min}	Minimum Plasma Drug Concentration
$C_{min,ss}$	Minimum Steady-state Plasma Drug Concentration
CKD-EPI	Chronic Kidney Disease Epidemiology Collaboration
CPK	Creatine Phosphokinase
CS	Clinically Significant
eCRF	Electronic Case Report Form
eGFR	Estimated Glomerular Filtration Rate
HBsAg	Hepatitis B Virus Antigen
HCV-Ab	Hepatitis C Virus Antibody
HED	Human Equivalent Doses
HIV-Ag/Ab	Human Immunodeficiency Virus Antigen/Antibody
IRB	Institutional Review Board
LDH	Lactate Dehydrogenase
KGCP	Korean Good Clinical Practice
MedDRA	Medical Dictionary for Regulatory Activities
MTD	Maximum Tolerance Dose
MRSD	Maximum Recommendation Starting Dose
NCS	Non Clinically Significant

NOAEL	No Observed Adverse Effect Level
PSV	Post Study Visit
PT	Preferred Term
PT	Prothrombin Time
PTF	Peak to Trough Fluctuation Ratio
RBC	Red Blood Cell
RPR	Rapid Plasma Reagin
SOC	System Organ Class
SOP	Standard Operating Procedure
T_{max}	Time to reach Maximum (peak) Plasma Concentration
$T_{max,ss}$	Time to reach Maximum (peak) Plasma Concentration at Steady State
$t_{1/2}$	Terminal Elimination Half-life
V_d/F	Apparent Volume of Distribution
$V_{d,ss}/F$	Apparent steady-state Volume of Distribution
V_z/F	Apparent Volume of Distribution Terminal Phase
WBC	White Blood Cell

◈ CLINICAL STUDY SYNOPSIS

**Title of Study**	A dose blocked-randomized, double-blind, placebo-controlled, single and multiple dosing, dose-escalation phase I clinical trial to evaluate the safety, tolerability, pharmacokinetics/pharmacodynamics of KDS2010 and food effect of KDS2010 on bioavailability after oral administration of KDS2010 in healthy young and elderly subjects
**Phase of Development**	Phase I
**Objectives**	• Primary objective: To evaluate the safety, tolerability and pharmacokinetics of a single and multiple oral dose of KDS2010 in healthy young and elderly subjects.<br>• Secondary objective: To evaluate the pharmacodynamics of a single and multiple oral dose of KDS2010 in healthy young and elderly subjects.
**Principal Investigators**	Department of Clinical Pharmacology and Therapeutics, Seoul National University Hospital<br>SeungHwan Lee, MD, PhD
**Sponsor**	NeuroBiogen Co., Ltd.<br>2nd Floor, 9, Myeongdal-ro, Seocho-gu, Seoul, Republic of Korea
**Institution**	Clinical Trials Center/ Clinical Research Institute, Seoul National University Hospital<br>101, Daehak-ro, Jongno-gu, Seoul, Republic of Korea
**Institution of Sample Analysis**	APACE<br>103, Daehak-ro, Jongno-gu, Seoul, Republic of Korea<br>BIO COMPLETE Co., Ltd.<br>603 and 604, Digital-ro, Guro-gu, Seoul, Republic of Korea
**Study Duration**	24 months from IRB approval date
**Target subject**	Healthy young and elderly
**Investigational Product**	• KDS2010 30 mg, 240 mg<br>• KDS2010 30 mg, 240 mg placebo
**Expected Target Disease**	Alzheimer's disease
**Study design**	A dose blocked-randomized, double-blind, placebo-controlled, single and multiple dosing, dose-escalation phase I clinical trial
**Number of Subjects**	• **Total number of subjects: 88**<br>This study is the exploratory phase 1 study to evaluate the safety, tolerability and pharmacokinetic characteristics of KDS2010 after single or multiple oral administrations. Therefore, it is desirable to proceed with the minimum number of subjects within the limit that satisfies the purpose of the study. So this study is planned with a target number of up to 88 patients, 8 in each cohort (including placebo).<br><br>• **Single ascending dose study (including food effect study): Total 48 subjects**

Cohort	Dosing groups	Dosage		Number of subjects
1	30 mg	Test group	KDS2010 30 mg × 1	6
		Control group	KDS2010 30 mg placebo × 1	2
2	60 mg	Test group	KDS2010 30 mg × 2	6
		Control group	KDS2010 30 mg placebo × 2	2
3†	120 mg (food effect)	Test group	KDS2010 30 mg × 4	6
		Control group	KDS2010 30 mg placebo × 4	2
4	240 mg	Test group	KDS2010 240 mg × 1	6
		Control group	KDS2010 240 mg placebo × 1	2
5	480 mg	Test group	KDS2010 240 mg × 2	6

		Control group	KDS2010 240 mg placebo × 2	2
6	960 mg	Test group	KDS2010 240 mg × 4	6
		Control group	KDS2010 240 mg placebo × 4	2

[†]For cohort 3, a fixed sequence, 2 period, cross over study will be conducted to evaluate the effect of food on the bioavailability of KDS2010. (If the dose escalation plan is changed, the dose for the evaluation of food effects may also be changed.)

- **Multiple ascending dose study[††]: Total 40 subjects**

Cohort	Dosing groups	Dosage		Number of subjects
1	60 mg	Test group	KDS2010 30 mg × 2	6
		Control group	KDS2010 30 mg placebo × 2	2
2	120 mg	Test group	KDS2010 30 mg × 4	6
		Control group	KDS2010 30 mg placebo × 4	2
3	240 mg	Test group	KDS2010 240 mg × 1	6
		Control group	KDS2010 240 mg placebo × 1	2
4	480 mg	Test group	KDS2010 240 mg × 2	6
		Control group	KDS2010 240 mg placebo × 2	2
5	120 mg (elderly)	Test group	KDS2010 30 mg × 4	6
		Control group	KDS2010 30 mg placebo × 4	2

[††]In the case of a multiple ascending dose study, subjects are administered once a day for 7 days.

Subjects

- **Inclusion Criteria**

1) Young subjects: Healthy Korean or Caucasian volunteers between 19 and 45 years of age at the screening visit
   Elderly subjects: Healthy Korean volunteers over 65 years and under 85 years of age at the screening visit
   ※ Caucasian who were born in Europe, have lived outside Europe for less than 10 years, and both parents and grandparents were of European origin
2) Women: Except for surgical infertility (bilateral fallopian tube occlusion, hysterectomy, bilateral salpingectomy, bilateral oophorectomy, etc.), those who tested negative in the urine pregnancy test (hCG) performed at screening
3) Subjects with body weight between 55.0 kg and 90.0 kg and Body Mass Index (BMI) between 18.0 kg/m^2 and 30.0 kg/m^2 at the screening visit
   ※ Body Mass Index = body weight(kg)/ $[height(m)]^2$
4) Subjects who completely understand the nature of study and make a voluntary decision to participate in this clinical study and provide the informed consent.
5) Subjects who are considered to be suitable to participate in this study based on physical examination, clinical laboratory test results and medical history

- **Exclusion Criteria**

1) Subjects with a clinical evidence or history of hepatic, renal, neurologic, immunologic, respiratory, endocrine, hematologic, oncologic, cardiovascular, psychiatric disease (However, in the case of an elderly person with a history of mild disease, participation is possible if the researcher determines that the drug can be discontinued at least 2 weeks before the expected first dose or 5 times or more before the half-life.)
2) Subjects with a history of gastrointestinal disease (e.g., gastrointestinal ulcers, gastritis, stomach cramp, gastroesophageal reflux disease, Crohn's disease, etc.) or surgery (except for simple appendectomy or herniotomy), which can influence the safety and pharmacokinetic evaluation of investigational drugs
3) For all women of childbearing age, except for surgical infertility, those who are pregnant, may be pregnant, or are lactating

4) Subjects with clinically significant abnormalities in the neurological examination performed at the screening visit
5) Subjects with any of the following vital signs at the screening visit
   - ✓ Systolic blood pressure (SBP): < 90 mmHg or > 140 mmHg
   - ✓ Diastolic blood pressure (DBP): < 50 mmHg or > 90 mmHg
   - ✓ Heart rate: < 50 bpm or > 90 bpm
6) Subjects with any of the following clinical laboratory test results at the screening visit
   - ✓ AST, ALT: > upper normal limit × 1.5
   - ✓ Total bilirubin: > upper normal limit × 1.5
   - ✓ CPK: > upper normal limit × 1.5
   - ✓ Creatinine clearance calculated by CKD-EPI equation: < 60 mL/min/1.73m^2
   - ✓ Subjects with a positive result in the serology test (syphilis test, Hepatitis B surface antigen, Hepatitis C antibody, Human immunodeficiency virus(HIV) antibody)
7) Subjects with any of the following ECG results at the screening visit
   - ✓ PR > 210 msec
   - ✓ QRS complex > 120 msec
   - ✓ QTcB > 450 msec
   - ✓ Other, clinically significant arrhythmias
8) Subjects with a history of hypersensitivities including drug allergies (caused by aspirin, antibiotics, etc.), or a history of clinically significant hypersensitivities
9) Subjects with a history of alcohol or drug abuse, or with positive reaction to any drug of concern for abuse/misuse at the urine drug screening test
10) Subjects who had taken or are expected to take any prescription drug or herbal medicine within 2 weeks or any over the counter (OTC) drug within 1 week prior to the first administration day (however, subjects fulfilling other conditions could participate in the study at the investigator's discretion)
11) Subjects who had taken an inducer or inhibitor of drug metabolism such as barbiturates etc. within 1 month prior to the first administration day
12) Subjects who had participated in another clinical study (including bioequivalence studies) within 6 months prior to the first administration day
13) Subjects who had donated whole blood or component blood, or received transfusion within 3 months prior to the first administration day
14) Subjects who drink continuously (more than 21 units/week, 1 unit = 10 g of pure alcohol) or are unable to stop drinking during the period from 3 days prior to the first administration day to the end of the study
15) Smokers (except for whom quitted smoking prior to the first administration day for at least 3months) or who cannot stop smoking during the period from 3 months prior to the first administration day to the end of the study
16) Subjects who cannot stop taking grapefruit or food containing grapefruit during the period from 3 days prior to the first administration day to the end of the study
17) Subjects who have taken excessive amounts of caffeine (> 5 units/day) on a continuous basis or who cannot stop taking caffeine or food containing caffeine (coffee, tea(black tea, green tea, etc.), carbonated drink, coffee-flavored milk, nutritional tonics, etc.) during the period from 3 days prior to the first administration day to the end of the study
18) During the entire study period and for at least 3months after the last administration of the investigational drug, subjects who are unable to use an appropriate, medically accepted double contraception method or medically acceptable contraceptive method for themselves or their spouse or partner and who do not agree not to donate sperm by that period.
   ※ Medically acceptable contraceptive methods
   - ✓ Use of an intrauterine device with a proven rate of pregnancy failure in the spouse (or partner)
   - ✓ Simultaneous use of a barrier contraception (for male or female) and spermicide
   - ✓ Surgery of the person himself or his spouse(or partner) (vasectomy, salpingectomy/ligation,

<table>
<tr><td></td><td>hysterectomy)
19) Otherwise, subjects judged by the investigator to be inappropriate for inclusion in the study</td></tr>
<tr><td>Study Design</td><td>

- **Single ascending dose study (30 mg, 60 mg, 240 mg, 480mg, 960 mg)**

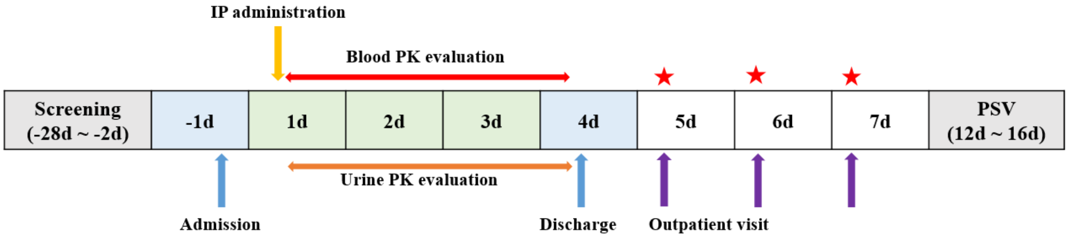

Volunteers for this study are selected as subjects if they are judged eligible for this study by undergoing screening procedures such as history taking, physical examinations, and clnical laboratory tests within 4 weeks (-28 ~ -2d) before the first administration.

Subjects are randomized after admission to the Clinical Trials Center, Seoul National University Hospital in the afternoon of the day of admission (-1d), and have dinner around 6 PM. On the dosing day (1d), the investigational drug is orally administered with 150mL of water at around 9 AM (reference time, 1d 0h), and food including drinking water is prohibited from 1 hour before administration to 4 hours after administration. Thereafter, the subjects conduct the study according to the predefined schedule and discharge at 4d. After discharge, subjects visit the Clinical Trials Center on the morning of 5d, 6d, and 7d to perform the scheduled procedures.

The subjects who complete whole study conduct the Post Study Visit(PSV) between 12d and 16d at their convenience.

- **Single dose study (120 mg, Food effect study)**

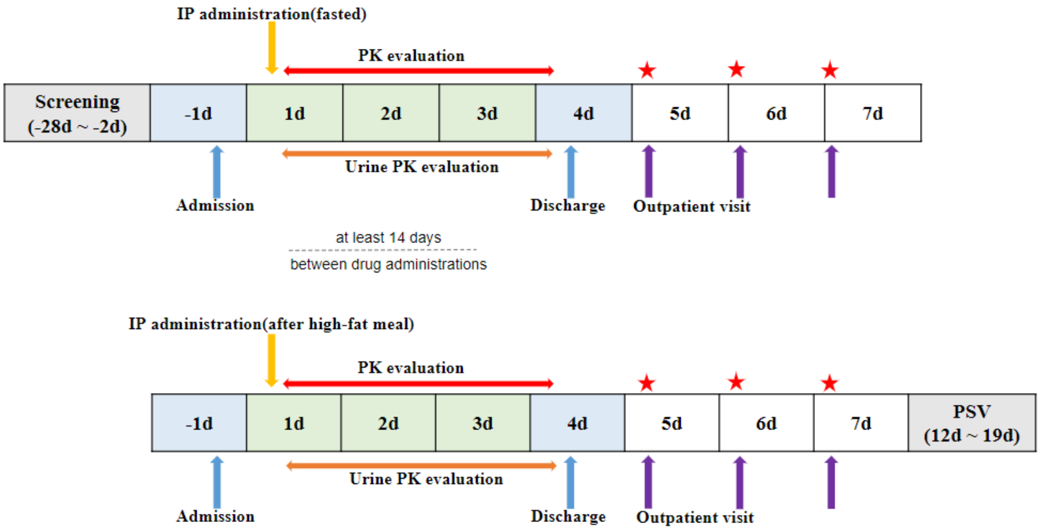

In the single dose study 120 mg dose group, subjects are hospitalized over 2 periods with an interval of at least 14 days between the dosing to evaluate the effect of food on the investigational drug. Both the administration in the fasted state (phase 1) and the administration after a high-fat meal(more than 900Kcal, more than 35% fat) (phase 2) will proceed. However, if the dose escalation plan is changed, the dose for the evaluation of food effects may also be changed.

- **Multiple ascending dose study (60mg, 120 mg, 240 mg, 480 mg, 120 mg(elderly))**

</td></tr>
</table>

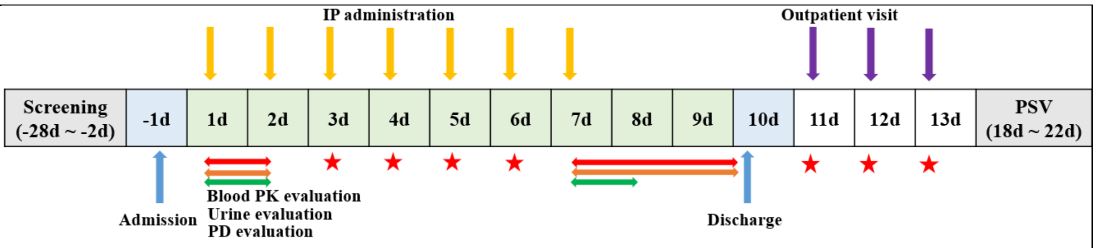

Volunteers for this study are selected as subjects if they are judged eligible for this study by undergoing screening procedures such as history taking, physical examinations, and clinical laboratory tests within 4 weeks (-28 ~ -2d) before the first administration.

Subjects are randomized after admission to the Clinical Trials Center, Seoul National University Hospital in the afternoon of the day of admission (-1d), and have dinner around 6 PM. On the dosing day (1d), the investigational drug is orally administered with 150mL of water at around 9 AM (reference time, 1d 0h), and food including drinking water is prohibited from 1 hour before administration to 4 hours after administration. (For the elderly, drinking water is prohibited for up to 2 hours and food intake is prohibited for up to 4 hours after administration.) Thereafter, the subjects conduct the study according to the predefined schedule and the administration of the investigational drug is continued in the hospitalized state for the repeated administration period (7 days). Subjects should maintain a fasting state for at least 10 hours before administration, and food including drinking water is prohibited from 1hour before administration. (For the elderly, drinking water is prohibited for up to 2 hours and food intake is prohibited for up to 4 hours after administration)

On 1d and 7d, drinking water and meals are prohibited until 4 hours after the end of administration, and on the other administration days except for 1d and 7d, food including drinking water is prohibited until 2 hours after the end of administration. On 2d, 3d, 4d, 5d, and 6d, pharmacokinetic blood sampling is performed before administration to confirm that steady state is reached. On the morning of 7d, after oral administration of the investigational drug once, examination and evaluation are performed according to the planned schedule and then discharged on 10d. After discharge, subjects visit the Clinical Trials Center on the morning of 11d, 12d, and 13d to perform the scheduled procedures.

The subjects who complete whole study conduct the Post Study Visit(PSV) between 18d and 22d at their convenience.

- **Dose Escalation Criteria**

When safety data within the last pharmacokinetic sampling day of all subjects in each dose group are available, the next dose level can proceed if it is determined that safety and tolerability have been adequately demonstrated through the review of the internal safety review committee (SRC) composed of the Investigators and the Sponsor. The elderly dose group(120 mg) of the multiple dose study proceeds after the maximum tolerated dose (MTD) group or the 480 mg dose group is completed. When proceeding from a single dose study to a multiple dose study, and when determining the administration dose in the elderly dose group within the multiple dose study, the appropriate administration dose is determined after review by the Safety Review Committee (SRC) including independent experts.

When determining whether safety and tolerability have been adequately demonstrated, the Principal Investigator remains blind to the treatment group (test group or placebo group). However, when the treatment information is critical to the subject safety and/or dose escalation decision, the Principal Investigator could be unblinded.

- **Dose Escalation Stopping Criteria**

After evaluating the severity of adverse events(safety test results(including clinical laboratory tests, vital signs, etc.) and adverse reactions of all subjects who administered the investigational drug) based on the Common Terminology Criteria for Adverse Events (CTCAE) version 5.0 or the latest version, the Principal Investigator can decide whether or not to discontinue the entire study. If two or more

	subjects in a dose group have a dose limiting toxicity defined as a Grade 3 or more than one subject in a dose group have a dose-limiting toxicity defined as a Grade 4 AE related to the investigational drug, the dose escalation may be stopped through discussion.
**Study Evaluations**	• **Safety and Tolerability Evaluation**<br>➢ Adverse events (AEs) including subjective/objective symptoms and signs etc.,<br>➢ Vital signs, physical examinations, clinical laboratory tests, 12-lead ECGs<br>➢ Neurologic examination (Full or brief)<br><br>• **Pharmacokinetic Evaluation**<br>Single ascending dose study(30 mg, 60 mg, 240 mg, 480 mg, 960 mg)<br>➢ Blood sampling timepoints: 1d 0h (pre-dose), 0.33, 0.75, 1, 2, 3, 4, 6, 8, 12, 24, 48, 72, 96, 120, 144 h post-dose<br>➢ Urine collection intervals: 1d 0h (pre-dose baseline spot-urine), 0 ~ 12h, 12 ~ 24h (2d 0h), 24 ~ 48h (3d 0h), 48 ~ 72h (4d 0h) post-dose<br>➢ Primary endpoints: AUC_{last}, AUC_{inf}, C_{max}, T_{max}, $t_{1/2}$, CL/F, V_z/F, fe, CL_R for KDS2010<br>➢ Secondary endpoints: AUC_{last}, AUC_{inf}, C_{max}, T_{max}, $t_{1/2}$, metabolic ratio for KDS8170(main metabolite of KDS2010)<br><br>Single dose study(120 mg, Food effect study)<br>➢ Blood sampling timepoints: 1d 0h (pre-dose), 0.33, 0.75, 1, 2, 3, 4, 6, 8, 12, 24, 48, 72, 96, 120, 144 h post-dose in phase 1 and 2<br>➢ Urine collection intervals: 1d 0h (pre-dose baseline spot-urine), 0 ~ 12h, 12 ~ 24h (2d0h), 24 ~ 48h (3d0h), 48 ~ 72h (4d0h) post-dose in phase 1 and 2<br>➢ Primary endpoints: AUC_{last}, AUC_{inf}, C_{max}, T_{max}, $t_{1/2}$, CL/F, V_z/F, fe, CL_R for KDS2010<br>➢ Secondary endpoints: AUC_{last}, AUC_{inf}, C_{max}, T_{max}, $t_{1/2}$, metabolic ratio for KDS8170(main metabolite of KDS2010)<br><br>Multiple ascending dose study(60mg, 120 mg, 240 mg, 480 mg, 120 mg(elderly)<br>➢ Blood sampling timepoints: 1d 0h (pre-dose), 0.33, 0.75, 1, 2, 3, 4, 6, 8, 12h, 2d 0h(pre-dose), 3d 0h(pre-dose), 4d 0h(pre-dose), 5d 0h(pre-dose), 6d 0h(pre-dose), 7d 0h(pre-dose), 0.33, 0.75, 1, 2, 3, 4, 6, 8, 12, 24 h (8d 0h), 9d 0h, 10d 0h, 11d 0h, 12d 0h, 13d 0h post-dose<br>➢ Urine collection intervals: 1d 0h (pre-dose baseline spot-urine), 0 ~ 12h, 12 ~ 24h (2d 0h), 7d 0h (pre-dose baseline spot-urine), 0 ~ 12h, 12 ~ 24h (8d 0h), 24 ~ 48h (9d 0h), 48 ~ 72h (10d 0h) post-dose<br>➢ Primary endpoints: AUC_{tau}, $C_{max,ss}$, $C_{min,ss}$, $C_{av,ss}$, $T_{max,ss}$, $t_{1/2}$, PTF (peak to trough fluctuation ratio), CL_{ss} /F, Vd_{ss}/F, fe, CL_R, accumulation ratio (Ra) for KDS2010<br>➢ Secondary endpoints: AUC_{tau}, $C_{max,ss}$, $C_{min,ss}$, $C_{av,ss}$, $T_{max,ss}$, $t_{1/2}$, PTF (peak to trough fluctuation ratio), accumulation ratio (Ra), metabolic ratio for KDS8170(main metabolite of KDS2010)<br><br>• **Exploratory Pharmacodynamic Evaluation**<br>Multiple ascending dose study (60mg, 120 mg, 240 mg, 480mg, 120 mg(elderly)<br>➢ Blood sampling timepoints: 1d/7d 0h (pre-dose), 2, 24h post-dose<br>➢ Endpoints: MAO-B activity
**Statistical Analysis**	• **Safety and Tolerability Analysis**<br>For safety/tolerability analysis, the abnormal findings observed in safety evaluation lists such as the occurrence pattern of adverse events, vital signs and clinical laboratory results are described, and if necessary, each list is compared according to appropriate classification such as dosing group. Medical history and AEs are coded using the MedDRA (ver. 24.0 or later).<br><br>• **Pharmacokinetic Analysis**<br>After calculating pharmacokinetic parameters for each subject by noncompartmental methods using an appropriate and validated pharmacokinetic software (Phoenix WinNonlin® version 8.0 or newer,

	Certara, CA, USA), descriptive statistics are summarized by appropriate classification such as dosing group.<br>To evaluate the pharmacokinetic linearity according to the dose increase, the relationship between C_{max}, AUC(AUC_{inf}, AUC_{last}) and the administered dose is evaluated through regression analysis, and C_{max}, AUC corrected for the administered dose are compared between dosing groups through a parametric or non-parametric statistical test. In addition, parameters such as T_{max}, $t_{1/2}$, CL/F can be compared between dosing groups through an appropriate method.<br>Food effect evaluation is performed by calculating the geometric mean ratio (GMR) of the pharmacokinetic parameters (C_{max}, AUC) after a high-fat meal to the fasting and its 90% confidence interval (CI).<br><br>• **Exploratory Pharmacodynamic Analysis**<br>After calculating exploratory pharmacodynamic parameters for each subject, descriptive statistics are summarized by appropriate classification such as dosing group.

◈ Study Flow Chart

Single ascending dose study(30 mg, 60 mg, 240 mg, 480 mg, 960 mg)

일정 / 항목	Screening	Treatment Period						Post Study Visit
	-28d ~ -2d	-1d	1d	2d	3d	4d	5d / 6d / 7d	12d ~ 16d
Informed consent	●							
Demographic data	●							
Medical history	●	●	●					
Inclusion/exclusion criteria check	●							
Admission		●						
Randomization[1]		●						
Discharge						●		
Out-patient Visit[2]							●	●
IP Administration[3]			●					
Vital signs[4]	●		●	●	●	●	●	●
Physical examination[5]	●		●	●	●	●	◐	●
12-lead ECG[6]	●		●	●	●	●	◐	●
Pregnancy test[7]	●	●						●
Clinical laboratory test[8]	●		●	●	●	●	◐	●
Alcohol breathing test	●							
Cotinine & Urine drug screening	●							
Neurological examination[9]	●		●			●	◐	●

1 Randomization: On -1d, randomization is performed and a randomization number is assigned.

2 Out-patient Visit: Subjects make outpatient visit to the Clinical Trials Center of Seoul National University Hospital on 5d, 6d, 7d, and post-study visit to perform the scheduled procedures.

3 IP Administration: On 1d around 9am, the investigational drug is orally administered with 150mL of water on an empty stomach. Set the 1d administration time to 1d 0h and apply it to the subsequent study schedule.

4 Vital signs: Blood pressure, heart rate, and body temperature are measured on screening, 1d pre-dose(0h), 1, 2, 4, 8, 12h post-dose, 2d 0h, 3d 0h, 4d 0h, 5d 0h, 6d 0h, 7d 0h and post-study visit.

5 Physical examination: Physical examination is performed on Screening, 1d pre-dose(0h), 2d 0h, 3d 0h, 4d 0h, 5d 0h, 7d 0h and post-study visit.

6 12-lead ECG: 12-lead ECG is measured on screening, 1d pre-dose(0h), 1, 2, 4, 8, 12h post-dose, 2d 0h, 3d 0h, 4d 0h, 5d 0h, 7d 0h and post-study visit.

7 Pregnancy test: For all women of childbearing potential except surgical infertility, urine hCG test is performed, and it is measured before screening, -1d before randomization, and at PSV.

8 Clinical laboratory test: Hematology, blood chemistry, coagulation and urinalysis are performed on screening, 1d pre-dose(0h), 2d 0h, 3d 0h, 4d 0h, 5d 0h, 7d 0h and post-study visit. Serology tests are performed only on screening.

9 Neurological examination: Neurological examination is performed on screening, 1d pre-dose(0h), 4h, 12h post-dose, 4d 0h, 7d 0h and post-study visit. A full neurological examination is performed on screening, post-study visit, and a brief neurological examination is performed during other periods.

일정 / 항목	Screening	Treatment Period						Post Study Visit
	-28d ~ -2d	-1d	1d	2d	3d	4d	5d / 6d / 7d	12d ~ 16d
Blood samplings for PK analysis[10]			●	●	●	●	●	
Urine collections for PK analysis[11]			●	●	●	●		
Adverse event monitoring			●	●	●	●	●	●
Concomitant medication monitoring	●	●	●	●	●	●	●	●

[10] Blood samplings for PK analysis: 1d 0h (pre-dose), 0.33, 0.75, 1, 2, 3, 4, 6, 8, 12, 24, 48, 72, 96, 120, 144h post-dose

[11] Urine collections for PK analysis: 1d 0h (pre-dose), 0 ~ 12h, 12 ~ 24h (2d 0h), 24 ~ 48h (3d 0h), 48 ~ 72h (4d 0h) post-dose

Single dose study(120 mg, Food effect study)

일정 / 항목	Screening	Treatment Period						Post Study Visit
	-28d ~ -2d	Phase1 -1d<br>Phase2 -1d	Phase1 1d<br>Phase2 1d	Phase1 2d<br>Phase2 2d	Phase1 3d<br>Phase2 3d	Phase1 4d<br>Phase2 4d	Phase1 5d / 6d / 7d<br>Phase2 5d / 6d / 7d	12d ~ 19d[1]
Informed consent	●							
Demographic data	●							
Medical history	●	●	●					
Inclusion/exclusion criteria check	●							
Admission[2]		●						
Randomization[3]		◐						
Discharge						●		
Out-patient Visit[4]							●	●
IP Administration[5]			●					
Vital signs[6]	●		●	●	●	●	●	●
Physical examination[7]	●		●	●	●	●	◐	●
12-lead ECG[8]	●		●	●	●	●	◐	●
Pregnancy test[9]	●	●						●
Clinical laboratory test[10]	●		●	●	●	●	◐	●
Alcohol breathing test	●							
Cotinine & Urine drug screening	●							
Neurological examination[11]	●		●			●	◐	●

1 Post Study Visit: On 12~19d from the phase 2 drug administration, subjects make post study visit.

2 Admission: The admission date of each phase is separated by at least 14 days.

3 Randomization: On phase1 -1d, randomization is performed and a randomization number is assigned.

4 Out-patient Visit: Subjects make outpatient visit to the Clinical Trials Center of Seoul National University Hospital on phase 1/2 5d, 6d, 7d and post-study visit to perform the scheduled procedures.

5 IP Administration: The assigned investigational drug is orally administered with 150mL of water after fasting (phase1 1d) or high-fat meal (phase2 1d) around 9 am. Set the phase1 1d administration time to 1d 0h and apply it to the subsequent study schedule.

6 Vital signs: Blood pressure, heart rate, and body temperature are measured on screening, phase 1/2 1d pre-dose(0h), 1, 2, 4, 8, 12h post-dose, 2d 0h, 3d 0h, 4d 0h, 5d 0h, 6d 0h, 7d 0h and post-study visit.

7 Physical examination: Physical examination is performed on screening, phase 1/2 1d pre-dose(0h), 2d 0h, 3d 0h, 4d 0h, 5d 0h, 7d 0h and post-study visit.

8 12-lead ECG: 12-lead ECG is measured on screening, phase 1/2 1d pre-dose(0h), 1, 2, 4, 8, 12h post-dose, 2d 0h, 3d 0h, 4d 0h, 5d 0h, 7d 0h and post-study visit.

9 Pregnancy test: For all women of childbearing potential except surgical infertility, urine hCG test is performed, and it is measured before screening, phase1 -1d before randomization, phase2 -1d and at PSV.

10 Clinical laboratory test: Hematology, blood chemistry, coagulation and urinalysis are performed on screening, phase 1/2 1d pre-dose(0h), 2d 0h, 3d 0h, 4d 0h, 5d 0h, 7d 0h and post-study visit. Serology tests are performed only on screening.

11 Neurological examination: Neurological examination is performed on screening, phase 1/2 1d pre-dose(0h), 4h, 12h post-dose, 4d 0h, 7d 0h and post-study visit. A full neurological examination is performed on screening, post-study visit, and a brief neurological examination is performed during other phases.

일정 / 항목	Screening	Treatment Period						Post Study Visit
	-28d ~ -2d	Phase1 -1d<br>Phase2 -1d	Phase1 1d<br>Phase2 1d	Phase1 2d<br>Phase2 2d	Phase1 3d<br>Phase2 3d	Phase1 4d<br>Phase2 4d	Phase1 5d / 6d / 7d<br>Phase2 5d / 6d / 7d	12d ~ 19d[1]
Blood samplings for PK analysis[12]			●	●	●	●	●	
Urine collections for PK analysis[13]			●	●	●	●		
Adverse event			●	●	●	●	●	●
Concomitant medication monitoring	●	●	●	●	●	●	●	●

[12] Blood samplings for PK analysis: phase 1/2 1d 0h (pre-dose), 0.33, 0.75, 1, 2, 3, 4, 6, 8, 12, 24, 48, 72, 96, 120, 144h post-dose

[13] Urine collections for PK analysis: phase 1/2 1d 0h (pre-dose), 0 ~ 12h, 12 ~ 24h (2d 0h), 24 ~ 48h (3d 0h), 48 ~ 72h (4d 0h) post-dose

Multiple ascending dose study (60mg, 120 mg, 240 mg, 480 mg, 120 mg(elderly))

일정 / 항목	Screening	Treatment Period											Post Study Visit
	-28d ~ -2d	-1d	1d	2d	3d	4d	5d	6d	7d	8d	9d	10d	11d / 12d / 13d	18d ~ 22d
Informed consent	●													
Demographic data	●													
Medical history	●	●	●											
Inclusion/exclusion criteria check	●													
Admission		●												
Randomization[1]		●												
Discharge												●		
Out-patient Visit[2]													●	●
IP Administration[3]			●	●	●	●	●	●	●					
Vital signs[4]	●		●	●	●	●	●	●	●	●	●	●	●	●
Physical examination[5]	●		●	●	●		●		●	●	●	●	◐	●
12-lead ECG[6]	●		●	●	●		●		●	●	●	●	◐	●
Pregnancy test[7]	●	●												●
Clinical laboratory test[8]	●		●	●	●		●		●	●	●	●	◐	●
Alcohol breathing test	●													
Cotinine & Urine drug screening	●													
Neurological examination[9]	●		●		●		●		●			●	◐	●

[1] Randomization: On -1d, randomization is performed and a randomization number is assigned.

[2] Out-patient Visit: Subjects make outpatient visit to the Clinical Trials Center of Seoul National University Hospital between 8 and 10 am 11d, 12d, 13d and post-study visit to perform the scheduled procedures.

[3] IP Administration: On 1d~7d around 9am, the investigational drug is orally administered with 150mL of water on an empty stomach. Set the 1d administration time to 1d 0h and apply it to the subsequent study schedule.

[4] Vital signs: Blood pressure, heart rate, and body temperature are measured on screening, 1d pre-dose(0h), 1, 2, 4, 8, 12h post-dose, 2d 0, 2h, 3d 0, 2h, 4d 0, 2h, 5d 0, 2h, 6d 0, 2h, 7d pre-dose(0h), 1, 2, 4, 8, 12h post-dose, 8d 0h, 9d 0h, 10d 0h, 11d 0h, 12d 0h, 13d 0h and post-study visit.

[5] Physical examination: Physical examination is performed on screening, 1d pre-dose(0h), 2d 0h, 3d 0h, 5d 0h, 7d pre-dose(0h), 8d 0h, 9d 0h, 10d 0h, 11d 0h, 13d 0h and post-study visit.

[6] 12-lead ECG: 12-lead ECG is measured on screening, 1d pre-dose(0h), 1, 2, 4, 8, 12h post-dose, 2d 0h, 3d 0h, 5d 0h, 7d pre-dose(0h), 1, 2, 4, 8, 12h post-dose, 8d 0h, 9d 0h, 10d 0h, 11d 0h, 13d 0h and post-study visit.

[7] Pregnancy test: For all women of childbearing potential except surgical infertility, urine hCG test is performed, and it is measured before screening, -1d before randomization, and at PSV.

[8] Clinical laboratory test: Hematology, blood chemistry, coagulation and urinalysis are performed on screening, 1d pre-dose(0h), 2d 0h, 3d 0h, 5d 0h, 7d pre-dose(0h), 8d 0h, 9d 0h, 10d 0h, 11d 0h, 13d 0h and post-study visit. Serology tests are performed only on screening.

[9] Neurological examination: Neurological examination is performed on screening, 1d pre-dose(0h), 4h, 12h post-dose, 3d 0h, 5d 0h, 7d pre-dose(0h), 4h, 12h post-dose, 10d 0h, 13d 0h and post-study visit. A full neurological examination is performed on screening, post-study visit, and a brief neurological examination is performed during other periods.

Blood samplings for PK analysis[10]			●	●	●	●	●	●	●	●	●	●	●	
Urine collections for PK analysis[11]			●	●					●	●	●	●		
Blood samplings for exploratory PD analysis[12]			●	●					●	●				
Adverse event			●	●	●	●	●	●	●	●	●	●	●	●
Concomitant medication monitoring	●	●	●	●	●	●	●	●	●	●	●	●	●	●

[10] Blood samplings for PK analysis: 1d 0h (pre-dose), 0.33, 0.75, 1, 2, 3, 4, 6, 8, 12h post-dose, 2d 0h, 3d 0h, 4d 0h, 5d 0h, 6d 0h, 7d 0h (pre-dose), 0.33, 0.75, 1, 2, 3, 4, 6, 8, 12h post-dose, 8d 0h, 9d 0h, 10d 0h, 11d 0h, 12d 0h, 13d 0h

[11] Urine collections for PK analysis: 1d 0h (pre-dose), 0 ~ 12h, 12 ~ 24h (2d 0h), 7d 0h (pre-dose), 0 ~ 12h, 12 ~ 24h (8d 0h), 24 ~ 48h (9d 0h), 48 ~ 72 h (10d 0h) post-dose

[12] Blood samplings for exploratory PD analysis: 1d/7d 0h (pre-dose) 2, 24h post-dose

◈ TABLE OF CONTENTS

SIGNATURE PAGE 2

◈ History of Clinical Study Protocol Revision 3

◈ Abbreviations and definition of terms 5

◈ CLINICAL STUDY SYNOPSIS 7

◈ Study Flow Chart 14

Single ascending dose study(30 mg, 60 mg, 240 mg, 480 mg, 960 mg) 14

Single dose study(120 mg, Food effect study) 16

Multiple ascending dose study (60mg, 120 mg, 240 mg, 480 mg, 120 mg(elderly)) 18

◈ TABLE OF CONTENTS 20

1. Study Title 25

2. Clinical Research Organization 25

3. Name and Title of Principal Investigator and Co-investigator 25

3.1. Principal Investigator 25

3.2. Co-investigator 25

3.3. Study Staff 25

4. Sponsor, Contract Research Organization and Sample Analysis Laboratory 26

4.1. Sponsor 26

4.2. Contract Research Organization (CRO) 26

4.3. PK/PD Sample Analysis Laboratory 26

5. Introduction 27

5.1. Background of Clinical Study 27

5.1.1. Background of the Disease 27

5.1.2. KDS2010 28

5.1.3. Preclinical Study Results 28

5.2. Rationale for Selection of Dose and Duration of Dosing 29

5.2.1. Selection of Dose ... 29
5.2.2. Duration of Dosing and Timepoints of Blood samplings/Urine collections ... 30
6. Study Objectives ... 31
7. Study Duration ... 31
8. Investigational Drugs ... 31
8.1. Test Drug ... 31
8.2. Control Drug ... 31
8.3. Packing and Labeling of Investigational Drugs ... 31
8.4. Management and Recording of Investigational Drugs ... 32
9. Study Subjects ... 32
9.1. Planned Subject Number and Rationale for sample size determination ... 32
9.2. Inclusion Criteria/Exclusion Criteria ... 33
9.2.1. Inclusion Criteria ... 33
9.2.2. Exclusion Criteria ... 34
9.3. Withdrawal Criteria ... 35
9.4. Replacement of Study Subjects ... 36
10. Study Plan ... 37
10.1. Overall Design and Plan of the Study ... 37
10.2. Blinding and Unblinding Procedures ... 39
10.2.1. Blinding ... 39
10.2.2. Unblinding Procedures ... 39
10.3. Assignment of Subject Identification Number ... 39
10.3.1. Screening Number ... 39
10.3.2. Randomization Number ... 39
10.4. Administration of Investigational Drugs ... 40
10.4.1. Administration methods ... 40

10.4.2. Administration compliance 40

10.4.3. Concomitant Medications 40

10.4.4. Prohibited Concomitant Medications and Foods 41

10.5. Dose Escalation to the Next Dose Level 41

10.6. Dose Escalation Stopping Criteria 42

10.7. Observations & Assessments 42

10.7.1. Screening test 42

10.7.2. Admission and Pharmacokinetic Evaluation Period 43

10.7.3. Post-Study Visit 48

10.8. Subject Cautions/Restrictions 49

10.9. Blood Samplings/Urine collection, Sample Storage and Analysis Methods for PK, PD Analysis 50

10.9.1. Blood Samplings/Urine collection, Sample Storage and Analysis Methods for PK Analysis 50

10.9.2. Blood Samplings, Sample Storage and Analysis Methods for Exploratory PD Analysis ... 51

11. Criteria for Suspension and Early Termination of Clinical Study 51

11.1. Suspension of Clinical Study 51

11.2. Early Termination 51

11.3. Protocol Deviations 52

12. Safety/Tolerability and PK, PD Assessments 52

12.1. Safety/Tolerability Assessments 52

12.1.1. Safety/Tolerability Endpoint 52

12.1.2. Safety/Tolerability Analysis 52

12.2. Pharmacokinetic Aseessments 52

12.2.1. Pharmacokinetic Endpoints 52

12.2.2. Pharmacokinetic Analysis 53

12.3. Pharmacodynamic Assessments 53

12.3.1. Pharmacodynamic Endpoints 53
12.3.2. Pharmacodynamic Analysis 53
13. Reporting of Adverse Events 53
13.1. Definition of Safety Terms 53
13.1.1. Adverse Event (AE) 53
13.1.2. Adverse Drug Reaction (ADR) 53
13.1.3. Serious Adverse Event/Adverse Drug Reaction (AE/ADR) 53
13.1.4. Suspected Unexpected Serious Adverse Reaction (SUSAR) 54
13.2. Recording and Reporting of Adverse Events 54
13.3. Reporting of Serious Adverse Events 55
13.4. Adverse Events and Causal Relationship Evaluation with Investigational Drugs 55
13.4.1. Severity 55
13.4.2. Actions Taken in relation to Adverse Events 55
13.4.3. Outcome of Adverse Events 56
13.4.4. Progress of Adverse Events 56
13.4.5. Causal Relationship with Investigational Drugs 56
13.5. Follow up of Adverse Events 57
14. Analytical and Statistical Considerations 58
14.1. Analysis Populations 58
14.2. Statistical Analysis Methods 58
14.3. Interim Analysis 58
15. Ethics of Research 59
16. Data Processing and Quality Assurance 59
16.1. Source Documents 59
16.2. Case Report Forms 59
16.3. Data Entry Process 59

16.4. Storage of Study Documents 59
16.5. Data Security 59
16.6. Confidentiality and Publication of Study Results 59
17. Other Considerations Required for Conduct of Safe and Scientific Clinical Study 60
17.1. Institutional Review Board (IRB) 60
17.2. Clinical Study Institution 60
17.3. Informed Consent Procedures 60
17.4. Actions to Secure Subject Safety 60
17.4.1. Subjects Confidentiality 61
17.4.2. Storage and Disposal of Human Derived Materials 61
17.5. Data Quality Control and Quality Assurance 61
17.6. Protocol Amendments 61
18. References 63
19. Appendices 63
20. Annex 63

1. Study Title

A dose blocked-randomized, double-blind, placebo-controlled, single and multiple dosing, dose-escalation phase I clinical trial to evaluate the safety, tolerability, pharmacokinetics/pharmacodynamics of KDS2010 and food effect of KDS2010 on bioavailability after oral administration of KDS2010 in healthy young and elderly subjects

2. Clinical Research Organization

Clinical Trials Center/ Clinical Research Institute, Seoul National University Hospital
101, Daehak-ro, Jongno-gu, Seoul, Republic of Korea (zip code: 03080)

3. Name and Title of Principal Investigator and Co-investigator

3.1. Principal Investigator

Department of Clinical Pharmacology and Therapeutics, Seoul National University Hospital
SeungHwan Lee, MD, PhD

3.2. Co-investigator

Department of Clinical Pharmacology and Therapeutics, Seoul National University Hospital
Professor In-Jin Jang MD, PhD

Department of Clinical Pharmacology and Therapeutics, Seoul National University Hospital
Professor Jaeseong Oh MD, PhD

3.3. Study Staff

Annex 3. List of Investigator and Pharmacist

4. Sponsor, Contract Research Organization and Sample Analysis Laboratory

4.1. Sponsor

NeuroBiogen Co., Ltd.
2nd Floor , 9, Myeongdal-ro, Seocho-gu, Seoul, Republic of Korea

4.2. Contract Research Organization (CRO)

C&R Research
412, Yeoksam-ro, Gangnam-gu, Seoul, Republic of Korea

4.3. PK/PD Sample Analysis Laboratory

APACE
103, Daehak-ro, Jongno-gu, Seoul, Republic of Korea

BIO COMPLETE Co., Ltd.

603 and 604, Digital-ro, Guro-gu, Seoul, Republic of Korea

5. Introduction

5.1. Background of Clinical Study

5.1.1. Background of the Disease

Dementia is a syndrome in which severe impairments in memory, concentration, language and cognition are progressively progressed over a long period of time due to damage and loss of nerve cells, ultimately leading to loss of mental and social ability. It refers to a disease in which cognitive impairment and personality changes occur acquired while maintaining a normal intellectual level during the growth period. Dementia is caused by the destruction of cranial nerves due to various causes and is accompanied by general disorders of mental functions such as memory impairment, speech impairment, judgment impairment, acquisition impairment, fecal/urinary incontinence, paranoid thinking, and aphasia. And during the course of disease, psychiatric symptoms such as depression, personality disorder and aggression may also be accompanied. Although the medical professionals are paying attention to the causality caused by aging and heredity, the exact cause of disease and treatment have not yet been identified. Diseases classified as dementia include Alzheimer's disease, vascular dementia, senile dementia, and dementia caused by head injury.

The typical cause of disease is Alzheimer's disease. Alzheimer's disease is divided into sporadic type and familial type depending on the cause. More than 80-90% of Alzheimer's disease are sporadic, the cause of which is not yet known, and it occurs mainly in the elderly population over 65 years of age. In the rest, mutations in genes such as APP, PS type 1 and type 2 are the main cause, and symptoms appear with a 100% chances in the population under the age of 65. According to the data from the Central Dementia Center of the Ministry of Health and Welfare, according to data from the International Alzheimer's Association (ADI), the number of Alzheimer's disease patients worldwide is known to be 50 million as of 2018, and expected to be about 82 million in 2030 and about 131.5 million in 2050. The number of patients with Alzheimer's disease aged 65 or older in Korea was about 780,000 in 2019 and is estimated to be about 3.32 million in 2060.

Currently, there is no fundamental curative agent for Alzheimer's disease, and existing drugs are AChEI that blocks the decomposition of deficient acetylcholine or disease modifying agents, which alleviate the rate of disease progression and temporarily improve cognitive functions, but the accompanying side effects remain a problem that reduces adaptability. Alzheimer's disease drugs used in clinical practice include AChEIs [acetylcholine esterase inhibitors: Aricept®(donepezil), Exelon®(rivastigmine), Razadyne®(galantmine)] and NMDA antagonists [Namenda®(memantine)]. Among them, Aricept(donepezil) is used in a wide range regardless of the severity of the symptoms of Alzheimer's disease, but all drugs have only a passive action to the extent of alleviating the symptoms.

Representative targets for the development of therapeutic agents for Alzheimer's disease include amyloid plaques and neurofibrillary tangles (tau), which are known as pathological changes and causes of Alzheimer's disease. Candidate drugs that inhibit the production of β-amyloid, a substance that forms amyloid plaques, have begun to be developed, and a representative target was BACE1(beta-secretase 1). However, most of the BACE1 therapeutic drugs failed due to various clinical side effects including ocular toxicity. Tau is a microtubule-associated protein that forms a tangle in nerve cells, and this tangle is known to be found in various degenerative brain diseases including AD. Tau exists in multiple intracellular and extracellular forms, sometimes moves in the brain, and has various characteristics such as hyperphosphorylation before tangles are formed. Antibody therapeutics that use vaccines to remove tau tangles through the body's immune system are under clinical trials or development.

In addition, monoamine oxidase–B (MAO-B) inhibitors are under development, based on the phenomenological mechanism of neuronal protection by MAO-B inhibition. It has been reported that MAO-B expression is increased in the cerebral cortex and hippocampus of Alzheimer's patients, and in particular, the activated MAO-B level in reactive astrocytes around amyloid plaques is increased more than 3-fold. This increase in MAO-B activity causes memory and cognitive impairments by over-production and secretion of GABA, an inhibitory signal transmitter, and MAO-B inhibitors are thought to be able to improve it. As a representative irreversible MAO-B inhibitor, rasagiline is currently the most prescribed drug in 7 major markets as a treatment for Parkinson's disease. Safinamide, a reversible MAO-B inhibitor, inhibits voltage gated sodium channel (VGSC) and excessive glutamate secretion, and has been commercially available as an adjuvant

treatment drug to maintain efficacy and relieve dyskinesia in the treatment of Parkinson's disease, such as Levodopa/Carbidopa. Although selegiline is used as a short-term treatment for Alzheimer's disease patients with cognitive deficits, it has a limitation that its efficacy is lost in long-term treatment, so it is not currently used as an indication for Alzheimer's disease.

5.1.2. KDS2010

As a reversible, selective MAO-B inhibitor, KDS2010 was developed as a long-term administrable drug by compensating for the shortcomings of the loss of therapeutic efficacy during long-term treatment in Alzheimer's disease patients. The main mechanism of action is as follows.

It reversibly inhibits MAO-B in reactive glial cells in the Alzheimer's dementia environment to restore neuronal firing by inhibiting over-generated GABA, and during long-term administration, it prevents alternative mechanisms for GABA production by DAO (diamine oxidase), thereby showing continuous efficacy. In addition, it suppresses H_2O_2 production and nitrosative stress by MAO-B of astrocytes in the inflammatory environment of the brain, thereby preventing brain nerve cell damage and protecting brain cells.

5.1.3. Preclinical Study Results

The results of preclinical study so far are as follows.

KDS2010 showed high MAO-B inhibitory activity in vitro (IC_{50} = 7.6 nM), and was confirmed 12500-fold higher MAO-B selectivity and reversibility compared to the irreversible inhibitor selegiline.

A passive avoidance test was performed after repeated administration of KDS2010 at doses of 1 mg/kg and 10 mg/kg for 2 weeks in APP/PS1 transgenic mice, an animal model of Alzheimer's disease. As a result, a statistically significant cognitive function improvement effect was confirmed at both 1 mg/kg and 10 mg/kg compared to the APP/PS1 control group. Also, as a result of measuring GABA receptor-mediated current in granule cells of the dentate gyrus adjacent to the hippocampus, the dose-dependent reduction of tonic GABA current was confirmed at 1 mg/kg and 10 mg/kg compared to the induced control group. All of these effects were confirmed to be dose-dependent. After repeated administration at a dose of 10 mg/kg for 27 to 37 days, the Morris water maze test was performed, and as a result, statistically significant improvement in spatial learning and memory impairment was confirmed compared to the control group. In the 4-week repeated dosing test, it was confirmed that KDS2010 administration showed a statistically significant cognitive function effect compared to the control group, and neuronal firing was maintained at a level similar to that of the normal group at 2 weeks and 4 weeks. On the other hand, selegiline showed cognitive function decline at the level of the induced control group at the 4th week of administration. And neuronal firing started to decrease at the 2nd week, and it was completely lost to the level of the induced control group at the 4th week. Also, it was confirmed that, unlike selegiline, a long-term administration of MAO inhibitors did not induce the DAO (diamine oxidase) compensatory mechanism, which is the cause of the loss of efficacy.

As a result of observing the effect of KDS2010 on cranial nerve cells in an inflammation model of brain lesions using diphtheria toxin receptor-inducible transgenic mice, when administered with KDS2010, it was confirmed that it can protect brain nerve cells in an inflammatory environment of brain lesions by increasing the decreased neuronal factors NeuN and MAP2(specifically expressed in the dendrites of nerve cells and an important factor for dendrite growth), and reducing the neurofilament (NF). And this effect was thought to appear through the inhibition of H_2O_2 production by MAO-B and the resulting nitrosative stress. Also, it was observed that brain atrophy was restored to the level of control group by KDS2010 administration, and a statistically significant increase in survival was confirmed.

As a result of the toxicity test of KDS2010 conducted under Good Laboratory Practice (GLP) to date, the approximate lethal dose in single oral (PO) and intravenous (IV) administration tests for Sprague-Dawley rats was 1400 mg/kg and more (PO) and 100 mg/kg and more (IV), respectively. And in a single oral dose escalation test for Cynomolgus Monkey, the approximate lethal dose was confirmed as 100 mg/kg or more. The repeated toxicity test was conducted using rodents (rat) and non-rodents (crab monkey), and as a result of oral

administration of the test substance to rats at a dose of 50-200 mg/kg daily for 2 weeks, there were no deaths. As a result of oral administration to rats at doses of 30, 50, 75 and 100 mg/kg once daily for 4 weeks, histopathological changes due to administration of the test substance were observed in the kidneys, bladder, testis, epididymis and lungs. Renal epithelial hyperplasia and inflammatory cells infiltration in the renal pelvis observed in the male and female dose groups of 75 mg/kg or more, germ cell degeneration/loss and seminiferous tubules atrophy observed in the male dose groups of 75 mg/kg or more, and vacuole/degeneration of outer medullary tubules and basophilic tubules observed in the female dose groups were judged to be adverse effects due to administration of the test substance. Proximal tubular hyaline granules in the kidney, basophilic tubules, testicular germ cell degeneration/loss, and alveolar macrophage aggregation in the lungs(females) were observed even after a 2-week recovery period. Accordingly, in repeated administrations of rats for 4 weeks, the NOAEL(No Observed Adverse Effect Level) was determined to be 50 mg/kg/day for males and 30 mg/kg for females. As a result of repeated oral administrations of 0, 25, 50 or 100 mg/kg/day for 2 weeks to Cynomolgus Monkey, a dying condition or mortality was observed in male and female dose groups of 100 mg/kg/day and in male dose group of 50 mg/kg/day, and a distal tubule/collecting duct dilatation and papillary duct epithelial cell hypertrophy related to the test substance were observed in male and female dose groups of 100 mg/kg/day. As a result of repeated oral administrations of KDS2010 in monkeys at doses of 0, 10, 20, and 40 mg/kg for 4 weeks and observation of recovery for 2 weeks, there were no adverse effects related to the test substance and the NOAEL was judged as 40 mg/kg for both males and females. As a result of the safety pharmacology test, no effect of the test substance was observed in the tests to evaluate the effects of central nervous system, respiratory system, and cardiovascular system, and the IC50 in the hERG assay was 7.8±1.7 μM. As a result of the genetic toxicology, the reverse mutation test, the chromosomal aberration assay, and the micronucleus assay were all negative.

In a pharmacokinetic study, plasma PK parameters were measured after a single oral administration of KDS2010 at 9 mg/kg in SD rats. As a result, C_{max} was 508.3 ng/mL, half-life ($t_{1/2}$) was 10.8 hours, AUC_{all} was 3003.1 hr*ng/mL. As a result of single oral administration of KDS2010 at a dose of 10 or 30 mg/kg in male monkeys, the peak plasma concentration (C_{max}) was reached at 2 hours after administration in the 10 mg/kg dose group and at 4 to 6 hours after administration in the 30 mg/kg dose group. In the elimination phase, the mean half-life($t_{1/2}$) was 13.7 hours at 10 mg/kg and 31.1 hours at 30 mg/kg. Systemic exposure (AUC_{last}) to the test substance was 30587 and 80770 ng*hr/mL, respectively. Systemic exposure (AUC_{last}) showed an increase rate similar to the increase rate of the dose in all test substance administration groups, and in the case of C_{max}, the increase rate was slightly lower than the increase rate of the dose.

As a result of oral administration of 14C radiolabeled KDS2010 to male SD rats at a dose of 10 mg/kg, the highest blood concentration (C_{max}) was 9250 ng eq./mL at 6.3 hours (T_{max}) after administration. The drug elimination half-life ($t_{1/2}$) was 143.2 h at the interval between 48 and 168 hours. At this time, $AUC_{0\text{-}inf}$ was 250000 ng eq.h/mL. The highest concentration (C_{max}) in plasma was 6620 ng eq./mL at 7 hours (T_{max}) after administration, and the elimination half-life ($t_{1/2}$) was 139.4 h at the interval between 48 and 168 hours. At this time, $AUC_{0\text{-}inf}$ was 192000 ng eq.h/mL. Blood to plasma ratio (RB) was 3.58 at 0.25 hours after administration and decreased to 1.01 at 48 hours. Accordingly, the RB value was determined to be between 1.11 and 1.22. As a result of measuring excretion into urine, feces, expiration, and bile, 6.9% was excreted in urine and 48.5% in feces, respectively, up to 168 hours after administration. Excretion to air through respiration was about 0.0% until 48 hours after administration, and 44.1% of the administered radioactivity concentration was present in the body (carcass). From this, the main excretion route of KDS2010 is feces, and it is judged that the excretion rate is slow.

In an experiment using BDC (bile duct-cannulated) rats, 71.5% was excreted in bile, 6.8% in urine, and 6.0% in feces until 48 hours after oral administration. Also, considering that this is higher than the total excretion amount in non-BDC rats, it appears that KDS2010 in bile was reabsorbed after oral administration, and enterohepatic circulation is considered to be involved in the pharmacokinetics of KDS2010.

5.2. Rationale for Selection of Dose and Duration of Dosing

5.2.1. Selection of Dose

According to the FDA guideline (Estimating the Maximum Safe Starting Dose in Initial Clinical Trials for

Therapeutics in Adult Healthy Volunteers), with considering the safety factor and the result of the rat that showed the smallest value among the human equivalent dose (HED) calculated using the body surface area from the NOAEL calculated in the 4-week repeated toxicity test for rats and cynomolgus monkeys, the maximum recommended starting dose (MRSD) was calculated. As a result of converting 0.484 mg/kg, a value obtained by dividing this by the safety factor of 10, into a dose for a healthy adult weighing 60 kg, the MRSD was calculated to be 29.04 mg, and the initial administration dose was set to 30 mg. As a result of a single oral dose toxicity test in rats, 700 mg/kg was reported as a maximum tolerated dose (MTD), and it was calculated as 6774.2 mg/60kg when converted to a human equivalent dose (HED). Therefore, the set maximum dose is expected to be suitable in terms of safety.

Species	NOAEL	HED	Safety factor (1/10)	MRSD (Safety factor 1/10)	Safety factor (1/30)	MRSD (Safety factor 1/30)
	mg/kg	mg/kg	mg/kg	mg/60kg	mg/kg	mg/60kg
Rat	30	4.84	0.484	**29.04**	0.161	**9.68**
Cynomolgus monkey	40	12.90	1.290	77.4	0.43	25.8

5.2.2. Duration of Dosing and Timepoints of Blood samplings/Urine collections

As a result of KDS2010's non-clinical pharmacokinetic study, the T_{max} in Cynomolgus monkey was about 2 to 6 hours. In addition, considering that the T_{max} of selegiline (ZELAPAR®) and safinamide (XADAGO®), which are similar drugs, are 0.75 hours and 1.83 to 2.83 hours, respectively, pharmacokinetic blood samplings before the estimated T_{max} (about 2 hours) were included at 3 or more time points, and the pharmacokinetic blood sampling time points of 1 to 3 hours after administration near the T_{max} was closely set. Also, as a result of estimating pharmacokinetic parameters in humans by the allometry method (correlation of CL=a*TBW0.75, V=b*TBW), the expected elimination half-life of the test drug in healthy adults (60 kg basis) was about 36 hours. As presumed, it was designed to have a pharmacokinetic blood collection up to 144 hours post-dose and a pharmacokinetic urine collection up to 72 hours post-dose. In the case of repeated administration, the dosing schedule for 7 days was set in consideration of securing sufficient safety and pharmacokinetic data, and the possibility of accumulation due to non-linear pharmacokinetic characteristics.

- Single ascending dose study

Blood samplings for PK analysis: 1d 0h (pre-dose), 0.33, 0.75, 1, 2, 3, 4, 6, 8, 12, 24, 48, 72, 96, 120, 144h post-dose

Urine collections for PK analysis: 1d 0h (pre-dose), 0 ~ 12h, 12 ~ 24h (2d 0h), 24 ~ 48h (3d 0h), 48 ~ 72h (4d 0h) post-dose

- Multiple ascending dose study

Blood samplings for PK analysis: 1d 0h (pre-dose), 0.33, 0.75, 1, 2, 3, 4, 6, 8, 12h post-dose, 2d 0h(pre-dose), 3d 0h(pre-dose), 4d 0h(pre-dose), 5d 0h(pre-dose), 6d 0h(pre-dose), 7d 0h(pre-dose), 0.33, 0.75, 1, 2, 3, 4, 6, 8, 12, 24 h post-dose(8d 0h), 9d 0h, 10d 0h, 11d 0h, 12d 0h, 13d 0h

Urine collections for PK analysis: 1d 0h (pre-dose), 0 ~ 12h, 12 ~ 24h, 7d 0h (pre-dose), 0 ~ 12h, 12 ~ 24h (8d 0h), 24 ~ 48h (9d 0h), 48 ~ 72h (10d 0h)

Blood samplings for PD analysis: 1d 0h (pre-dose), 2h, 24h, 7d 0h (pre-dose), 2h, 24h

6. Study Objectives

The objective of this study is to evaluate the safety, tolerability and pharmacokinetics of KDS2010 and to explore pharmacodynamics of KDS2010 after a single and multiple oral administration of KDS2010 in healthy young and elderly subjects.

7. Study Duration

24 months from the date of IRB approval

8. Investigational Drugs

8.1. Test Drug

General Name	KDS2010 tablet
Active ingredient and content	KDS2010 (main ingredient) 30 mg, 240 mg
Formulation	Tablet
Storage condition	Tight, light-resistant containers, Room temperature (1~30℃)
Shelf life	36 months from date of manufacture
Manufacturer	Patheon

8.2. Control Drug

Placebo with the same formulation as KDS2010 is as follows:

General Name	Placebo in KDS2010 tablets
Active ingredient and content	Contains excipients other than the main ingredient of the test drug
Formulation	Tablet
Storage condition	Tight, light-resistant containers, Room temperature (1~30℃)
Shelf life	36 months from date of manufacture
Manufacturer	Patheon

8.3. Packing and Labeling of Investigational Drugs

Investigational drugs must be manufactured by the clinical study sponsor in accordance with regulations on the safety of medicines [Ordinance of the Prime Minister No. 1650, 2020.10.14. according to attached table 1, attached table 4], and then supplied to the clinical trial pharmacist of the clinical study institution. The test drug should be delivered to the clinical trial institution by the sponsor along with the label of the dosage for each subject, and the administering pharmacist divides the dosage into small portions at the time of dispensing.
In accordance with the regulations on drug manufacturing and quality control [Ministry of Food and Drug Safety Notice No. 2020-82, 2020.9.7 Attached Table 11. 7.7 Labeling matters to be entered], the sponsor attaches a label containing the following contents and then supplies it to the clinical trial pharmacist.

- A statement indicating that this product cannot be used for the purposes other than clinical study (ex. "For Clinical Trial Use Only")
- Code name of investigational drug or identified code (In the case of a blinded test, the test drug and control drug should be listed together, and in the open test, only the relevant group should be listed). If necessary, include the formulation, route of administration, quantity, content or titer of the active ingredient, etc.
- A batch number or code number that identifies the contents and packaging operation.
- The name, address and phone number(to enable inquiries about investigational drugs, clinical studies, and emergency unblinding related matters) of the sponsor (if the clinical study plan has been approved, the person who has received approval for the plan)
- Expiration(use-by) date (If tracking is possible by using a centralized electronic randomization system, it is also possible to indicate the year/month.
- Storage conditions

- ″Keep out of reach of children.” (Except when the investigational drug is not brought to the subject's home)
- A reference code that identifies the clinical study. However, if the sponsor of the clinical study deems it necessary, a reference code to identify the clinical study institution, the researcher and the sponsor may be written.
- The subject identification number, treatment number and visit number. However, if the sponsor acknowledges the specificity of unnecessary description in consideration of the characteristics of the corresponding clinical study, it may be documented and omitted.
- Name of investigator if the sponsor deems it necessary
- If the sponsor of the clinical study deems it necessary, method of use (the subject or the person administering the drug may refer to the administration manual or other documents)

The clinical trial pharmacist should confirm the receipt of the investigational drug and the number of investigational drug in writing, sign and manage them properly. They should ensure that the investigational drugs are administered to the subjects only in accordance with the protocol, and the records of investigational drugs provided to each subject and the management should be accurate.
The clinical trial pharmacist is a person in charge of only dispensing who has no interest in this clinical trial and prepares the drug in a separate space. Investigators in charge of administration and evaluation should be blinded so that independence can be maintained by delivering the control drug with the same properties without information on the drug.
When the investigational product is left unused, it is stored until the sponsor determines if it is destroyed or returned. When the study is completed, the all used(empty packs, boxes, etc.) or unused investigational drugs are returned to the sponsor and a copy of the drug management record is submitted to the sponsor's monitor in charge.

8.4. Management and Recording of Investigational Drugs

Responsibility for receipt, storage, prescription, return, and management of investigational drugs during the clinical study period rests with the principal investigator and the clinical trial pharmacist of the institution. Investigational drugs are delivered to the clinical trial pharmacist of the clinical study institution, and the clinical trial pharmacist performs tasks such as acceptance, inventory management, dispensing and returning drugs for each subject, and maintains related records. Neurobiogen Co., Ltd. or the monitor agent in charge ensures that the appropriate quantity is supplied and managed at the necessary time through smooth communication with the clinical trial pharmacist and periodic monitoring.
The clinical trial pharmacist should take back the empty containers and remaining drugs after administration of the investigational drugs, and return them to the sponsor together with the unused investigational drugs that have not been provided to the subject. When an appropriate reason occurs, the sponsor destroys the investigational drugs and documentates the evidence.

9. Study Subjects

9.1. Planned Subject Number and Rationale for sample size determination

This study is a phase 1 study to evaluate the safety, tolerability and pharmacokinetics of KDS2010 and to explore pharmacodynamics of KDS2010 after a single and multiple oral administration of KDS2010. Therefore, it is desirable to proceed with the minimum number of subjects within the limit that satisfies the purpose of the study, so this study is planned with a target number of up to 88 subjects, 8 in each cohort (including placebo).
If, according to the 9.3. Withdrawal Criteria, a subject drops out, and a replacement subject occurs according to the 9.4. Replacement of Study Subjects, the number of subjects may increase. (However, except for cases that only PSV visits are not conducted)

- **Single ascending dose study (including food effect study): Total 48 subjects**

Cohort	Dosing groups	Dosage		Number of subjects
1	30 mg	Test group	KDS2010 30 mg × 1	6
		Control group	KDS2010 30 mg placebo × 1	2
2	60 mg	Test group	KDS2010 30 mg × 2	6

		Control group	KDS2010 30 mg placebo × 2	2
3[†]	120 mg (food effect)	Test group	KDS2010 30 mg × 4	6
		Control group	KDS2010 30 mg placebo × 4	2
4	240 mg	Test group	KDS2010 240 mg × 1	6
		Control group	KDS2010 240 mg placebo × 1	2
5	480 mg	Test group	KDS2010 240 mg × 2	6
		Control group	KDS2010 240 mg placebo × 2	2
6	960 mg	Test group	KDS2010 240 mg × 4	6
		Control group	KDS2010 240 mg placebo × 4	2

[†] For cohort 3, a fixed sequence, 2 period, cross over study will be conducted to evaluate the effect of food on the bioavailability of KDS2010. (If the dose escalation plan is changed, the dose for the evaluation of food effects may also be changed.)

- **Multiple ascending dose study[††]: Total 40 subjects**

Cohort	Dosing groups	Dosage		Number of subjects
1	60 mg	Test group	KDS2010 30 mg × 2	6
		Control group	KDS2010 30 mg placebo × 2	2
2	120 mg	Test group	KDS2010 30 mg × 4	6
		Control group	KDS2010 30 mg placebo × 4	2
3	240 mg	Test group	KDS2010 240 mg × 1	6
		Control group	KDS2010 240 mg placebo × 1	2
4	480 mg	Test group	KDS2010 240 mg × 2	6
		Control group	KDS2010 240 mg placebo × 2	2
5	120 mg (elderly)	Test group	KDS2010 30 mg × 4	6
		Control group	KDS2010 30 mg placebo × 4	2

[††] In the case of a multiple ascending dose study, subjects are administered once a day for 7 days.

9.2. Inclusion Criteria/Exclusion Criteria

9.2.1. Inclusion Criteria

1) Young subjects: Healthy Korean or Caucasian volunteers between 19 and 45 years of age at the screening visit

   Elderly subjects: Healthy Korean volunteers over 65 years and under 85 years of age at the screening visit

   ※ Caucasian who were born in Europe, have lived outside Europe for less than 10 years, and both parents and grandparents were of European origin.

2) Women: Except for surgical infertility (bilateral fallopian tube occlusion, hysterectomy, bilateral salpingectomy, bilateral oophorectomy, etc.), those who tested negative in the urine pregnancy test (hCG) performed at screening

3) Subjects with body weight between 55.0 kg and 90.0 kg and Body Mass Index (BMI) between 18.0 kg/m2 and 30.0 kg/m2 at the screening visit

   ※ Body Mass Index = body weight(kg)/ [height(m)]^2

4) Subjects who completely understand the nature of study and make a voluntary decision to participate in this clinical study and provide the informed consent.

5) Subjects who are considered to be suitable to participate in this study based on physical examination, clinical laboratory test results and medical history

9.2.2. Exclusion Criteria

1) Subjects with a clinical evidence or history of hepatic, renal, neurologic, immunologic, respiratory, endocrine, hematologic, oncologic, cardiovascular, psychiatric disease (mood disorders, obsessive-compulsive disorder, etc.) (However, in the case of an elderly person with a history of mild disease, participation is possible if the researcher determines that the drug can be discontinued at least 2 weeks before the expected first dose or 5 times or more before the half-life.)
2) Subjects with a history of gastrointestinal disease (e.g., gastrointestinal ulcers, gastritis, stomach cramp, gastroesophageal reflux disease, Crohn's disease, etc.) or surgery (except for simple appendectomy or herniotomy), which can influence the safety and pharmacokinetic evaluation of investigational drugs
3) For all women of childbearing age, except for surgical infertility, those who are pregnant, may be pregnant, or are lactating
4) Subjects with clinically significant abnormalities in the neurological examination performed at the screening visit
5) Subjects with any of the following vital signs at the screening visit
   - ✓ Systolic blood pressure (SBP): < 90 mmHg or > 140 mmHg
   - ✓ Diastolic blood pressure (DBP): < 50 mmHg or > 90 mmHg
   - ✓ Heart rate: < 50 bpm or > 90 bpm
6) Subjects with any of the following clinical laboratory test results at the screening visit
   - ✓ AST, ALT: > upper normal limit × 1.5
   - ✓ Total bilirubin: > upper normal limit × 1.5
   - ✓ CPK: > upper normal limit × 1.5
   - ✓ Creatinine clearance calculated by CKD-EPI equation: < 60 mL/min/1.73m2
   - ✓ Subjects with a positive result in the serology test (syphilis test, Hepatitis B surface antigen, Hepatitis C antibody, Human immunodeficiency virus(HIV) antibody)
7) Subjects with any of the following ECG results at the screening visit
   - ✓ PR > 210 msec
   - ✓ QRS complex > 120 msec
   - ✓ QTcB > 450 msec
   - ✓ Other, clinically significant arrhythmias
8) Subjects with a history of hypersensitivities including drug allergies (caused by aspirin, antibiotics, etc.), or a history of clinically significant hypersensitivities
9) Subjects with a history of alcohol or drug abuse, or with positive reaction to any drug of concern for abuse/misuse at the urine drug screening test
10) Subjects who had taken or are expected to take any prescription drug or herbal medicine within 2 weeks or any over the counter (OTC) drug within 1 week prior to the first administration day (however, subjects fulfilling other conditions could participate in the study at the investigator's discretion)

11) Subjects who had taken an inducer or inhibitor of drug metabolism such as barbiturates etc. within 1 month prior to the first administration day

12) Subjects who had participated in another clinical study (including bioequivalence studies) within 6 months prior to the first administration day

13) Subjects who had donated whole blood or component blood, or received transfusion within 3 months prior to the first administration day

14) Subjects who drink continuously (more than 21 units/week, 1 unit = 10 g of pure alcohol) or are unable to stop drinking during the period from 3 days prior to the first administration day to the end of the study

15) Smokers(except for whom quitted smoking prior to the first administration day for at least 3months) or who cannot stop smoking during the period from 3 months prior to the first administration day to the end of the study

16) Subjects who cannot stop taking grapefruit or food containing grapefruit during the period from 3 days prior to the first administration day to the end of the study

17) Subjects who have taken excessive amounts of caffeine (> 5 units/day) on a continuous basis or who cannot stop taking caffeine or food containing caffeine (coffee, tea(black tea, green tea, etc.), carbonated drink, coffee-flavored milk, nutritional tonics, etc.) during the period from 3 days prior to the first administration day to the end of the study

18) During the entire study period and for at least 3months after the last administration of the investigational drug, subjects who are unable to use an appropriate, medically accepted double contraception method or medically acceptable contraceptive method for themselves or their spouse or partner and who do not agree not to donate sperm by that period.

    ※ Medically acceptable contraceptive methods

    - ✓ Use of an intrauterine device with a proven rate of pregnancy failure in the spouse (or partner)
    - ✓ Simultaneous use of a barrier contraception (for male or female) and spermicide
    - ✓ Surgery of the person himself or his spouse(or partner) (vasectomy, salpingectomy/ligation, hysterectomy)

19) Otherwise, subjects judged by the investigator to be inappropriate for inclusion in the study

9.3. Withdrawal Criteria

'Enrollment' means that the subject who participated in the study (ie, signed the informed consent form) met the inclusion/exclusion criteria and was assigned a subject randomization number according to randomization. 'Dropout' means that an enrolled subject does not complete the study. Dropout could be decided at any time during the study period for the following reasons. Dropouts of enrolled subjects from the study should be reported on the eCRF with the date of the dropout, date of the last study drug administration and the reason for the dropout.

1) When it is judged that it is difficult for the subject to participate in a clinical study any longer due to a clinically unacceptable serious adverse event or abnormality such as a clinical laboratory test value

2) When the subject takes any medication that is expected to influence the safety and pharmacokinetic characteristics of investigational drug without the permission of the investigator

3) When the major protocol deviation significant enough to consider discontinuation of the study is identified during the study, such as the subject was found not to be eligible based on inclusion/exclusion criteria after enrollment.

4) When the subject does not wish to proceed with investigational drug administration or withdraws consent during the study period

5) When the subject does not visit on time and cannot be contacted

6) In the cases where the principal investigator or sub-investigator decides to withdraw the subject from the clinical study for other reasons

Those who drop out after administering the investigational drug visit the clinical study institution within 7 days if possible of the dropout (or the first recognition of dropout) and conduct an equivalent post-study visit. However, if the subject refuses to visit, if possible, concomitant medications and lists related to adverse events should be checked by phone. Also, if the dropped out subject has clinically significant abnormal test results, follow-up should be continued. Subjects who have been dropped out due to adverse events or deviations of safety standards should be followed up until symptoms subside and abnormal laboratory test values return to standard values, or there is a satisfactory explanation for the observed changes. If follow-up is not possible, it is considered as 'failed to follow up'.
If discontinued due to a serious adverse event, the serious adverse event should be reported in accordance with <13.3. Reporting of Serious Adverse Events>.

9.4. Replacement of Study Subjects

In this clinical study, a small number of candidate subjects can be registered in advance in consideration of the dropout rate prior to the first administration, and when dropout occurs before the first administration, the candidate subject can be replaced with the formal subject.
As this clinical study is conducted for exploratory purposes, not hypothesis testing, there is no need to replace subjects even if they drop out. In order to maintain the ratio of subjects receiving the test drug to subjects receiving placebo by dose group, additional subjects may be registered to replace subjects who dropped out. However, in the case of a subject who dropped out due to an adverse event that occurred after administration of the investigational drug, the subject is not replaced.
In the case of replacing a subject who dropped out due to reasons other than adverse events (eg, withdrawal of consent, etc.), replaced subjects are selected in the order that they passed the screening test, and the subject number that has already been used cannot be used. In the case of subject replacement, the subject number of the subject to be replaced shall be marked so that it can be recognized that it is the replaced subject, including the last digit of the number of the subject who dropped out.

10. Study Plan

10.1. Overall Design and Plan of the Study

This clinical study was designed as a dose blocked-randomized, double-blind, placebo-controlled, single and multiple dosing, dose-escalation phase I clinical trial.

1) Single ascending dose study (30 mg, 60 mg, 240 mg, 480 mg, 960 mg)

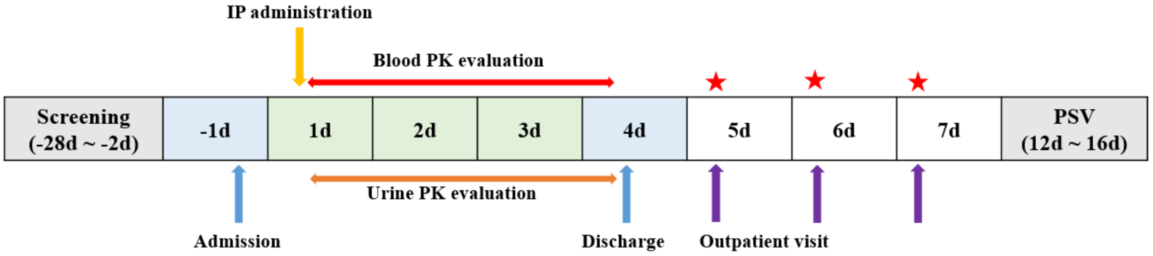

Volunteers for this study are selected as subjects if they are judged eligible for this study by undergoing screening procedures such as history taking, physical examinations, and clinical laboratory tests within 4 weeks (-28 ~ -2d) before the first administration.

Subjects are randomized after admission to the Clinical Trials Center, Seoul National University Hospital in the afternoon of the day of admission (-1d), and have dinner around 6 PM. On the dosing day (1d), the investigational drug is orally administered with 150mL of water at around 9 AM (reference time, 1d 0h), and food including drinking water is prohibited from 1 hour before administration to 4 hours after administration. Thereafter, the subjects conduct the study according to the predefined schedule and discharge at 4d. After discharge, subjects visit the Clinical Trials Center on the morning of 5d, 6d, and 7d to perform the scheduled procedures.

The subjects who complete whole study conduct the Post Study Visit(PSV) between 12d and 16d at their 4convenience.

2) Single dose study (120 mg, Food effect study)

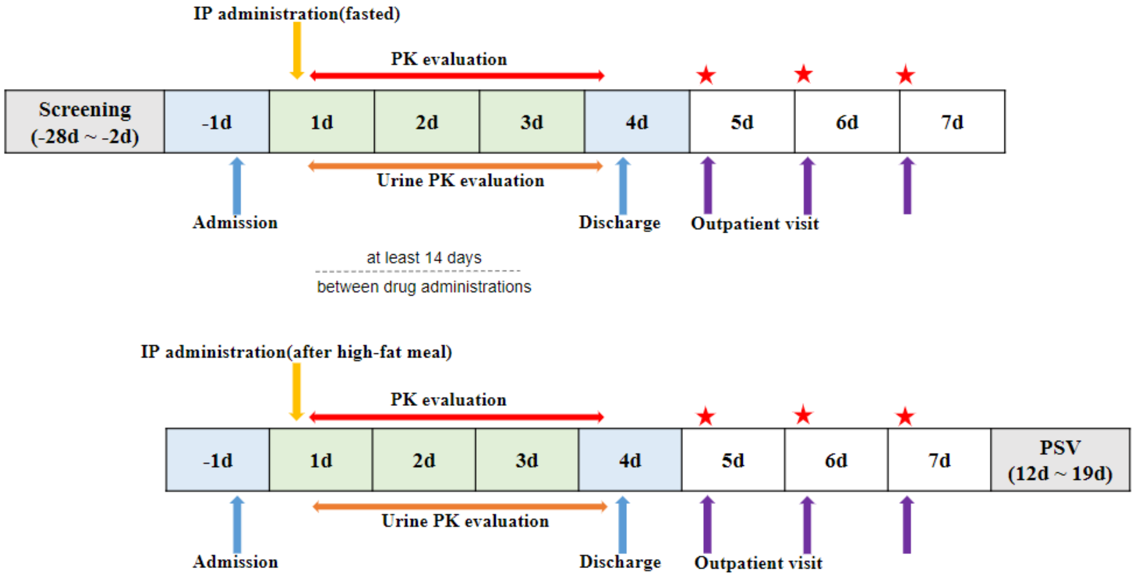

Volunteers for this study are selected as subjects if they are judged eligible for this study by undergoing screening procedures such as history taking, physical examinations, and laboratory tests within 4 weeks (-28 ~ -2d) before the first administration.

Subjects are randomized after admission to the Clinical Trials Center, Seoul National University Hospital in the afternoon of the day of admission (phase 1 -1d), and have dinner around 6 PM. On the dosing day of the first period (phase1 1d), the investigational drug is orally administered with 150mL of water at around 9 AM (reference time, phase 1d 0h), and food including drinking water is prohibited from 1 hour before administration to 4 hours after administration. Thereafter, the subjects conduct the study according to the predefined schedule and discharge at 4d. After discharge, subjects visit the Clinical Trials Center on the morning of 5d, 6d, and 7d to perform the scheduled procedures.

The 2nd dosing is planned with an at least 14 days after 1st dosing. The subject is admitted to the Clinical Trials Center, Seoul National University Hospital in the afternoon of the 2nd period admission day (phase 2 1d) and has dinner around 6 PM. On the dosing day of the second period (phase 2 1d), subjects start a high-fat meal (more than 900 kcal, more than 35% fat) 30 minutes before administration of the investigational drug and complete it within 20 minutes, and the investigational drugs are orally administered with 150mL of water at around 9 AM (reference time, phase1 1d 0h). Food including drinking water is prohibited from 1 hour before administration to 4 hours after administration. Thereafter, the subjects conduct the study according to the predefined schedule and discharge at 4d. After discharge, subjects visit the Clinical Trials Center on the morning of 5d, 6d, and 7d to perform the scheduled procedures.

The subjects who complete the whole study conduct the Post Study Visit(PSV) between 12d and 19d from the phase2 dosing date at their convenience.

3) Multiple ascending dose study (60mg, 120 mg, 240 mg, 480 mg, 120 mg(elderly)

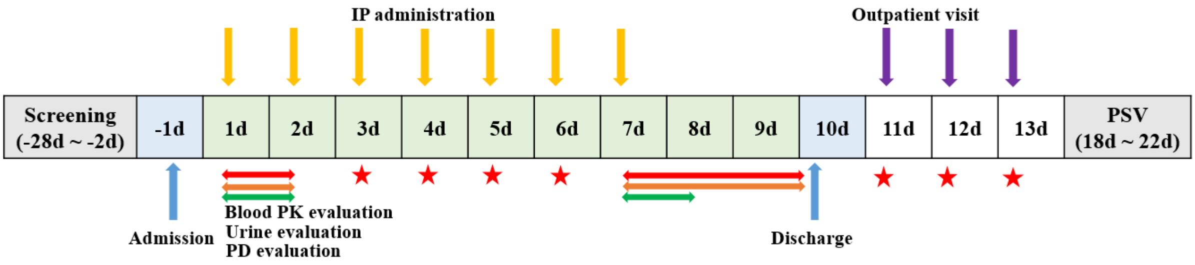

Volunteers for this study are selected as subjects if they are judged eligible for this study by undergoing screening procedures such as history taking, physical examinations, and clinical laboratory tests within 4 weeks (-28 ~ -2d) before the first administration.

Subjects are randomized after admission to the Clinical Trials Center, Seoul National University Hospital in the afternoon of the day of admission (-1d), and have dinner around 6 PM. On the dosing day (1d), the investigational drug is orally administered with 150mL of water at around 9 AM (reference time, 1d 0h), and food including drinking water is prohibited from 1 hour before administration to 4 hours after administration. (For the elderly, drinking water is prohibited for up to 2 hours and food intake is prohibited for up to 4 hours after administration.) Thereafter, the subjects conduct the study according to the predefined schedule and the administration of the investigational drug is continued in the hospitalized state for the repeated administration period (7 days). Subjects should maintain a fasting state for at least 10 hours before administration, and food including drinking water is prohibited from 1hour before administration. On 1d and 7d, drinking water and meals are prohibited until 4 hours after the end of administration (For the elderly, drinking water is prohibited for up to 2 hours and food intake is prohibited for up to 4 hours after administration.), and on the other administration days except for 1d and 7d, food including drinking water is prohibited until 2 hours after the end of administration. On 2d, 3d, 4d, 5d, and 6d, pharmacokinetic blood sampling is performed before administration to confirm that steady state is reached. On the morning of 7d, after oral administration of the investigational drug once, examination and evaluation are performed according to the planned schedule and then discharged on 10d. After discharge, subjects visit the Clinical Trials Center on the morning of 11d, 12d, and 13d to perform the scheduled procedures.

The subjects who complete whole study conduct the Post Study Visit(PSV) between 18d and 22d at their convenience.

10.2. Blinding and Unblinding Procedures

10.2.1. Blinding

The label indicated in the protocol is for the study drug and placebo provided only to pharmacies, and neither the principal investigator nor the subject cannot know the type of drug being administered before the end of the study. Clinical investigational drugs are monitored by a separate monitoring agent not involved in this clinical trial, and the monitoring agent and the managing pharmacist are excluded from blinding. The investigational drug manager must confirm that the appearance of the investigational drug is the same between the test drug and the placebo, that is, the test drug and the placebo are indistinguishable from each other before assignment of the investigational drug and code breaking. This clinical study is a dose blocked-randomized, double-blind, placebo-controlled, single and multiple dosing, dose-escalation phase I clinical trial and is conducted in a double-blind manner to exclude subjective judgments of subjects and investigators.
In order to maintain the double-blindness, a placebo with the same formulation and appearance that cannot be distinguished from the KDS2010 tablet will be used, and the packaging will be the same. By maintaining double-blindness for the investigator and the subject, make sure that the investigator or subject avoids bias in evaluating treatment effects and adverse events.
The person in charge of randomization generates a randomization code according to the randomization method and provides the randomization code to the laboratory management pharmacist. Accordingly, the managing pharmacist dispenses the investigational drug (test drug or placebo) assigned to each subject number. For each subject, the drug for clinical trial is determined according to their own randomization number, and the randomization code is not disclosed unless the randomization code is released after the clinical trial for each stage is completed. However, in case the blinding is removed due to the occurrence of an emergency such as a serious adverse reaction, the person in charge of randomization should make a blinding envelope with the randomization number and deliver it to the principal investigator and keep it. In addition, the blindfold bag must be properly stored until the end of the clinical trial by the principal investigator and can only be opened according to the established procedure.

10.2.2. Unblinding Procedures

If it is determined that the blinding should be removed due to the occurrence of an emergency that threatens the safety of the subject, the investigator must immediately notify Neurobiogen Co., Ltd. or the monitor of the contract research organization delegated by Neuro Biogen Co., Ltd. The monitor has an obligation to immediately contact the person in charge of Neurobiogen. After obtaining the relevant information, the person in charge of NeuroBiogen determines whether or not to remove the blinding in consultation with the investigator and leaves a document on the decision. In case that immediate contact with the sponsor Neurobiogen is not possible, the investigator must proceed with unblinding and inform Neurobiogen Co., Ltd. or the monitor of the contract research organization delegated by Neurobiogen Co., Ltd. as soon as possible of the breaking of the double blinding. And the reason for unblinding without consulting with the sponsor should be documented.
If the investigator becomes aware of the subject identification code during the study period, the investigator should endeavor to exclude bias in the evaluation of safety.
After the clinical study is over, all data is resolved through the 'Query'. When the database is confirmed to be complete and correct, the data will be locked and the randomization code information will be released. Subsequent changes to the database are possible only with the written consent of the sponsor and the database administrator.

10.3. Assignment of Subject Identification Number

10.3.1. Screening Number

Screening numbers are assigned to the subjects who provided a written consent to participate in the clinical study in the order of the informed consent form signed. The screening numbers start with S001 and will be given in regular sequence.

10.3.2. Randomization Number

On the day of admission (-1d), the randomization numbers are assigned in the order of passing the screening tests finally(If the subjects pass the screening tests on the same day, subject numbers will be assigned in the order of randomization numbers). They are randomly assigned to the test group or Control group according to the randomization number.
The randomization number consists of five digits (RXXXX) and has a certain rule of one letter (R) and four digits. The first number represents each single/multiple dose study (1: single ascending dose study; 2: multiple ascending dose study). The second number represents the cohort. The third digit indicates whether the subject is a replacement subject or not (0: existing subject; 1: first replacement subject). The last single digit represents the order of participating subjects. The randomization number assigned to each subject is used as a subject identification code to recognize the subject until the end of the clinical study. Subjects who withdraw consent or have the study discontinued for any reason retain their subject number regardless of the administration of investigational drugs.

**Single ascending dose study**		**Multiple ascending dose study**	
Cohort	Randomization number	Cohort	Randomization number
1	R1101 ~	1	R2101 ~
2	R1201 ~	2	R2201 ~
3	R1301 ~	3	R2301 ~
4	R1401 ~	4	R2401 ~
5	R1501 ~	5	R2501 ~
6	R1601 ~		

The randomization number is used as the subject identification code during the clinical study period. After generating the randomization list, the person in charge of randomization sends it to the clinical trial pharmacist. The clinical trial pharmacist dispenses investigational drugs for each subject according to the randomization list.
Until the end of this clinical study, all investigators, subjects, and those who conduct clinical study, except for the clinical trial pharmacist, must remain blinded. And if it is necessary to unblind during the clinical study, proceed according to the procedure mentioned in 10.2.2 of this clinical study protocol.

10.4. Administration of Investigational Drugs

10.4.1. Administration methods

After fasting for at least 10 hours, subjects should orally administer the investigational drug together with 150 mL of water on an empty stomach around 9:00 am on the day of administration of the investigational drug. (However, in the case of the food effect study among single ascending dose study, both the administration in the fasting state (phase 1) and the administration (phase 2) after a high-fat meal (900 Kcal or more, fat content 35% or more) are carried out.)

Subjects should swallow the investigational drug whole with 150 mL of water, and should not chew the drug before swallowing. Subjects should fast for 4 hours after administration, and should sit at 45 degrees or more or stand upright until 2 hours after administration.

10.4.2. Administration compliance

Compliance is ensured by administering all investigational drugs under the supervision of the investigator or the person who has been delegated by the investigator at the clinical study institution. Non-compliant subjects (e.g, those who are not present for a scheduled visit or who break the restrictions) may be excluded from the clinical study and will be recorded in the eCRF accordingly. The investigator administers the investigational drug to the subject and confirms the intake through oral examination.

10.4.3. Concomitant Medications

Subjects are prohibited from taking concomitant medications from the time of screening to the end of the study. However, if concomitant medication is used for medical need, the concomitant medication may be administered under the judgment of the investigator (principal investigator or doctor in charge). If it is expected that the drug taken by the subject arbitrarily may affect the safety, tolerability, and pharmacokinetic evaluation, which is the object of this clinical study, the subject may be withdrawn at the discretion of the investigator. For all administered concomitant medications, the drug name (ingredient name, brand name in case of combination), route of administration, daily dose, duration of administration (start and end), and reason for taking are recorded in the source document and eCRF.

10.4.4. Prohibited Concomitant Medications and Foods

- All prescription drugs and herbal medicines are prohibited from 2 weeks prior to first investigational drug administration to the end of clinical study.
- Antidepressants (SSRI, SNRI, tricyclic, tetracyclic, triazolopyridine) are prohibited from 2 weeks before administration of the investigational drug to 2 weeks after administration of the last investigational drug, and Fluoxetine is prohibited from 5 weeks before the administration of the investigational drug to 2 weeks after the last administration of the investigational drug.
- Dietary supplements or vitamin supplements including all over the counter drugs (OTCs) or liver function supplements are prohibited from 1 week prior to first investigational drug administration to the end of clinical study.
- Those who continuously drink alcohol (more than 21 units/week, 1 unit = 10 g of pure alcohol) cannot participate in the clinical study, and alcohol must be abstained from 3 days before the first administration to the end of the clinical study.
- Grapefruit, grapefruit juice, foods containing grapefruit, and caffeine/foods containing caffeine (coffee, tea (black tea, green tea, etc.), carbonated drinks, coffee-flavored milk, nutritional tonics, etc.) are prohibited during the period from 3 days before the first administration to the end of the clinical study.
- Foods rich in tyramine (cheese, processed meat, pickled vegetables, soybean paste, citrus fruits, etc.) are prohibited during the period from 1 day before the first administration to discharge.

10.5. Dose Escalation to the Next Dose Level

When safety data up to the the last pharmacokinetic sampling day of the all subjects for each dose group are available, the next dose level can proceed if it is determined that safety and tolerability have been adequately demonstrated through the review of the internal safety review committee (SRC) composed of the Investigators and the Sponsor. The elderly dose group(120 mg) of the multiple dose study proceeds after the maximum tolerated dose (MTD) group or the 480 mg dose group is completed. When proceeding from a single dose study to a multiple dose study, and when determining the administration dose in the elderly dose group within the multiple dose study, the appropriate administration dose is determined after review by the Safety Review Committee (SRC) including independent experts. The SRC is made up of the investigators, the sponsor, and cardiovascular or neurological experts.

When determining whether safety and tolerability have been adequately demonstrated, the Principal Investigator remains blind to the treatment group (test group or placebo group). However, when the treatment information is critical to the subject safety and/or dose escalation decision, the Principal Investigator could be unblinded.
Considering the drug's half-life after administration and study schedule in the single and multiple ascending dose study, safety and tolerability is evaluated within the last pharmacokinetic sampling day of all subjects in the dose group. If it is judged acceptable through the internal SRC review, proceed to the next dose level after discussion and the investigator's judgment has priority for adverse events occurring within the period.
After evaluating the severity of adverse events(safety test results(including clinical laboratory tests, vital signs, etc.) and adverse reactions of all subjects who administered the investigational drug) based on the Common

Terminology Criteria for Adverse Events (CTCAE) version 5.0 or the latest version, the Principal Investigator can decide whether or not to discontinue the entire study. In addition, if the following occurs, the dose escalation can be stopped through discussion between the investigator and the sponsor.

- When a Grade 3 or higher adverse drug reaction occurs in more than 1/4 of the subjects
- When a Grade 4 or higher adverse drug reaction occurs in one or more subjects

During this clinical study period, the administered dose of the investigational drug may be changed based on the results of the previous dose group. And in this case, it will proceed after obtaining approval from the Ministry of Food and Drug Safety to amend the clinical study protocol.

10.6. Dose Escalation Stopping Criteria

After evaluating the severity of adverse events(safety test results(including clinical laboratory tests, vital signs, etc.) and adverse reactions of all subjects who administered the investigational drug) based on the Common Terminology Criteria for Adverse Events (CTCAE) version 5.0 or the latest version, the Principal Investigator can decide whether or not to discontinue the entire study. If two or more subjects in a dose group have a dose limiting toxicity defined as a Grade 3 or more than one subject in a dose group have a dose-limiting toxicity defined as a Grade 4 AE related to the investigational drug, the dose escalation may be stopped through discussion.

10.7. Observations & Assessments

10.7.1. Screening test

The screening tests to review the eligibility of the subjects who voluntarily provided a written consent to participate in the clinical study will be performed within 4 weeks from the first administration, and subjects who have clinically significant abnormalities in the following tests will be excluded. Prior to the screening visit, perform the test after fasting for at least 8 hours. (However, if the screening test results are clinically significant, retesting can be carried out up to two times at the discretion of the investigator.)

In order to smoothly conduct clinical study, some subjects may participate as candidate subjects. Candidate subjects will be hospitalized on the same day as the formal subjects assigned with randomization numbers, and if there is a vacancy in the formal subject while participating as a candidate subject (e.g., no show of a formal subject or withdrawal of consent, etc.), the candidate subject may participate in the clinical study as a substitute for a formal subject. If there is no vacancy and the treatment group is terminated, the candidate subject may participate as a formal subject in the other treatment group with the next available schedule.

1) Explanation of consent form and obtaining written consent
2) Inclusion and exclusion criteria evaluation
3) Investigation of demographic data and measurement of height and weight

   ① At the time of screening, demographic information such as gender and age of the test subject is checked, and drinking and smoking history are asked.

   ② Measurement of height and weight

   - Height: To 1 decimal place, cm
   - Weight: Rounded from 2 decimal places to 1 decimal place, kg
   - Body mass index: Rounded from 2 decimal places to 1 decimal place, kg/m^2

   ※ Body mass index, kg/m^2 = weight (kg)/[height (m)]^2

4) Investigation of drug administration history and medical history

   Investigate recent medical history, including past medical history within 3 years, and drug administration

history within 5 weeks.

5) Physical examination

Physical examination includes examination of general condition, nutritional status, skin/mucous membranes, eyes (except for visual loss), otolaryngology, thyroid, lung/respiratory system, heart/circulatory system, abdomen, kidney/urogenital system, nerve/psychiatric system, spine/limb/tumors, peripheral circulation, lymphatic system, and other organs.

6) Vital signs

Vital signs include systolic blood pressure, diastolic blood pressure, pulse rate, and body temperature (tympanic membrane). Blood pressure and pulse rate are measured after maintaining a seated position for more than 3 minutes without sudden changes in position.

7) 12-Lead ECG

In addition to the basic recording, the ventricular rate (beats/min), PR interval (msec), QRS (msec), QT/QTcB (msec) at which automatic analysis & recording is output are recorded.

8) Pregnancy test

For all women of childbearing potential except surgical infertility, urine hCG test is performed.

9) Clinical laboratory test

Clinical laboratory tests include the following lists.

**Hematology test**	WBC with differential count (segmented neutrophil, eosinophil, basophil, lymphocyte, monocyte), RBC, hemoglobin, hematocrit, platelet
**Blood chemistry test**	AST, ALT, ALP, γ-GTP, LDH, CPK, glucose, total cholesterol, triglyceride, creatinine, BUN, uric acid, total bilirubin, total protein, albumin, sodium, potassium, calcium, chloride, phosphorus, eGFR (measured using the CKD-EPI formula)
**Coagulation test**	PT (INR), aPTT
**Urine test**	dipstick (color, pH, specific gravity, albumin, bilirubin, glucose, urobilinogen, ketone, nitrite, occult blood, WBC) microscopy (RBC, WBC, squamous cell, others)

10) Alcohol breathing test

11) Cotinine & Urine drug screening

Amphetamines, barbiturates, benzodiazepines, cannabinoids, cocaine, opiates, cotinine

12) Serology test

HBsAg, HCV-Ab, HIV-Ag/Ab, RPR

13) Neurological examination

A full neurological examination (Appendix 1) is performed.

14) Concomitant medication monitoring

15) Education on precautions for subjects

10.7.2. Admission and Pharmacokinetic Evaluation Period

10.7.2.1. Single ascending dose study(30 mg, 60 mg, 240 mg, 480mg, 960 mg)

1) Physical examination

Physical examination is performed on 1d pre-dose (0h), 2d 0h, 3d 0h, 4d 0h, 5d 0h, and 7d 0h.

Scheduled time point	Time window
1d 0h, 2d 0h, 3d 0h, 4d 0h	-60 min

2) Vital sign

Blood pressure, pulse rate, and body temperature(tympanic) are measured on 1d pre-dose (0h), 1, 2, 4, 8, 12h post-dose, 2d 0h, 3d 0h, 4d 0h, 5d 0h, 6d 0h, and 7d 0h.

Scheduled time point	Time window
1d 0h, 2d 0h, 3d 0h, 4d 0h	-60 min
1d 1, 2, 4, 8, 12h	±30 min

3) 12-Lead ECG

Electrography is performed on 1d pre-dose(0h), 1, 2, 4, 8, 12h post-dose, 2d 0h, 3d 0h, 4d, 0h, 5d 0h and 7d 0h.

Scheduled time point	Time window
1d 0h, 2d 0h, 3d 0h, 4d 0h	-60 min
1d 1, 2, 4h	±20 min
1d 8, 12h	±30 min

4) Pregnancy test

For all women of childbearing potential except surgical infertility, urine hCG test is performed before -1d randomization.

5) Clinical laboratory test

Clinical laboratory tests are performed on 1d pre-dose (0h), 2d 0h, 3d 0h, 4d 0h, 5d 0h, and 7d 0h.

Scheduled time point	Time window
1d 0h, 2d 0h, 3d 0h, 4d 0h	-60 min

Clinical laboratory tests include the following lists.

**Hematology test**	WBC with differential count (segmented neutrophil, eosinophil, basophil, lymphocyte, monocyte), RBC, hemoglobin, hematocrit, platelet
**Blood chemistry test**	AST, ALT, ALP, γ-GTP, LDH, CPK, glucose, total cholesterol, triglyceride, creatinine, BUN, uric acid, total bilirubin, total protein, albumin, sodium, potassium, calcium, chloride, phosphorus, eGFR (measured using the CKD-EPI formula)
**Coagulation test**	PT (INR), aPTT
**Urine test**	dipstick (color, pH, specific gravity, albumin, bilirubin, glucose, urobilinogen, ketone, nitrite, occult blood, WBC) microscopy (RBC, WBC, squamous cell, others)

6) Neurological examination

A brief neurological examination (Appendix 2) is performed on 1d pre-dose (0h), 4h, 12h post-dose, 4d 0h, and 7d 0h.

Scheduled time point	Time window
1d 0h, 4d 0h	-60 min
1d 4, 12h	±30 min

7) Blood samplings and urine collections for PK analysis

Blood samplings for PK analysis are performed on 1d 0h (pre-dose), 0.33, 0.75, 1, 2, 3, 4, 6, 8, 12, 24, 48, 72, 96, 120, 144h post-dose, and urine collections for PK analysis are performed on 1d 0h (pre-dose), 0 ~ 12h, 12 ~ 24h (2d 0h), 24 ~ 48h (3d 0h), 48 ~ 72h (4d 0h) post-dose.

Scheduled time point for urine collections	Time window
1d 0h	-60 min
1d 0~12h, 12~24h (2d 0h),<br>24~48h (3d 0h), 48~72h (4d 0h)	±30 min

10.7.2.2. Single dose study (120 mg, Food effect study)

1) Physical examination

Physical examination is performed on phase 1/2 1d pre-dose(0h), 2d 0h, 3d 0h, 4d 0h, 5d 0h, and 7d 0h.

Scheduled time point	Time window
phase 1/2 1d 0h, 2d 0h, 3d 0h, 4d 0h	-60 min

2) Vital sign

Blood pressure, pulse rate, and body temperature(tympanic) are measured on phase 1/2 1d pre-dose(0h), 1, 2, 4, 8, 12h post-dose, 2d 0h, 3d 0h, 4d 0h, 5d 0h, 6d 0h, and 7d 0h.

Scheduled time point	Time window
phase 1/2 1d 0h, 2d 0h, 3d 0h, 4d 0h	-60 min
phase 1/2 1d 1, 2, 4, 8, 12h	±30 min

3) 12-Lead ECG

Electrography is performed on phase 1/2 1d pre-dose(0h), 1, 2, 4, 8, 12h post-dose, 2d 0h, 3d 0h, 4d 0h, 5d 0h, and 7d 0h.

Scheduled time point	Time window
phase 1/2 1d 0h, 2d 0h, 3d 0h, 4d 0h	-60 min
phase 1/2 1d 1, 2, 4h	±20 min
phase 1/2 1d 8, 12h	±30 min

4) Pregnancy test

For all women of childbearing potential except surgical infertility, urine hCG test is performed before phase1 -1d randomization and phase2 -1d.

5) Clinical laboratory test

Clinical laboratory tests are performed on phase 1/2 1d pre-dose(0h), 2d 0h, 3d 0h, 4d 0h, 5d 0h, and 7d 0h.

Scheduled time point	Time window
phase 1/2 1d 0h, 2d 0h, 3d 0h, 4d 0h	-60 min

Clinical laboratory tests include the following lists.

**Hematology test**	WBC with differential count (segmented neutrophil, eosinophil, basophil, lymphocyte, monocyte), RBC, hemoglobin, hematocrit, platelet
**Blood chemistry test**	AST, ALT, ALP, γ-GTP, LDH, CPK, glucose, total cholesterol, triglyceride, creatinine, BUN, uric acid, total bilirubin, total protein, albumin, sodium, potassium, calcium, chloride, phosphorus, eGFR (measured using the CKD-EPI formula)
**Coagulation test**	PT (INR), aPTT
**Urine test**	dipstick (color, pH, specific gravity, albumin, bilirubin, glucose, urobilinogen, ketone, nitrite, occult blood, WBC) microscopy (RBC, WBC, squamous cell, others)

6) Neurological examination

A brief neurological examination (Appendix 2) is performed on phase 1/2 1d pre-dose(0h), 4h, 12h post-dose, 4d 0h, and 7d 0h.

Scheduled time point	Time window
phase 1/2 1d 0h, 4d 0h	-60 min
phase 1/2 1d 4, 12h	±30 min

8) Blood samplings and urine collections for PK analysis

Blood samplings for PK analysis are performed on phase 1/2 1d 0h (pre-dose), 0.33, 0.75, 1, 2, 3, 4, 6, 8, 12, 24, 48, 72, 96, 120, 144h post-dose, and urine collections for PK analysis are performed on phase 1/2 1d 0h (pre-dose), 0 ~ 12h, 12 ~ 24h (2d 0h), 24 ~ 48h (3d 0h), 48 ~ 72h (4d 0h) post-dose.

Scheduled time point for urine collections	Time window
phase 1/2 1d 0h	-60 min
phase 1/2 1d 0~12h, 12~24h (2d 0h),<br>24~48h (3d 0h), 48~72h (4d 0h)	±30 min

10.7.2.3. Multiple ascending dose study (60mg, 120 mg, 240 mg, 480mg, 120 mg(elderly))

1) Physical examination

Physical examination is performed on 1d pre-dose(0h), 2d 0h, 3d 0h, 5d 0h, 7d pre-dose(0h), 8d 0h, 9d 0h, 11d 0h, and 13d 0h.

Scheduled time point	Time window
1d 0h, 2d 0h, 3d 0h, 5d 0h, 7d 0h, 8d 0h, 9d 0h	-60 min

2) Vital sign

Blood pressure, pulse rate, and body temperature(tympanic) are measured on 1d pre-dose(0h), 1, 2, 4, 8, 12h post-dose, 2d 0, 2h, 3d 0, 2h, 4d 0, 2h, 5d 0, 2h, 6d 0, 2h, 7d pre-dose(0h), 1, 2, 4, 8, 12h post-dose, 8d 0h, 9d 0h, 10d 0h, 11d 0h, 12d 0h, and 13d 0h.

Scheduled time point	Time window
1d 0h, 2d 0h, 3d 0h, 4d 0h, 5d 0h, 6d 0h,<br>7d 0h, 8d 0h, 9d 0h, 10d 0h	-60 min
1d 1, 2, 4, 8, 12h<br>2d 2h, 3d 2h, 4d 2h, 5d 2h, 6d 2h	±30 min

7d 1, 2, 4, 8, 12h	

3) 12-Lead ECG

Electrography is performed on 1d pre-dose(0h), 1, 2, 4, 8, 12h post-dose, 2d 0h, 3d 0h, 5d 0h, 7d pre-dose(0h), 1, 2, 4, 8, 12h post-dose, 8d 0h, 9d 0h, 10d 0h, 11d 0h, and 13d 0h.

Scheduled time point	Time window
1d 0h, 2d 0h, 3d 0h, 5d 0h, 7d 0h<br>8d 0h, 9d 0h, 10d 0h	-60 min
1d 1, 2, 4h, 7d 1, 2, 4h	±20 min
1d 8, 12h, 7d 8, 12h	±30 min
11d 0h, 13d 0h	±120 min

4) Pregnancy test

For all women of childbearing potential except surgical infertility, urine hCG test is performed before -1d randomization.

5) Clinical laboratory test

Clinical laboratory tests are performed on 1d pre-dose(0h), 2d 0h, 3d 0h, 5d 0h, 7d pre-dose(0h), 8d 0h, 9d 0h, 10d 0h, 11d 0h, and 13d 0h.

Scheduled time point	Time window
1d 0h, 2d 0h, 3d 0h, 5d 0h, 7d 0h, 8d 0h,<br>9d 0h, 10d 0h	-60 min

Clinical laboratory tests include the following lists.

**Hematology test**	WBC with differential count (segmented neutrophil, eosinophil, basophil, lymphocyte, monocyte), RBC, hemoglobin, hematocrit, platelet
**Blood chemistry test**	AST, ALT, ALP, γ-GTP, LDH, CPK, glucose, total cholesterol, triglyceride, creatinine, BUN, uric acid, total bilirubin, total protein, albumin, sodium, potassium, calcium, chloride, phosphorus, eGFR (measured using the CKD-EPI formula)
**Coagulation test**	PT (INR), aPTT
**Urine test**	dipstick (color, pH, specific gravity, albumin, bilirubin, glucose, urobilinogen, ketone, nitrite, occult blood, WBC) microscopy (RBC, WBC, squamous cell, others)

6) Neurological examination

A brief neurological examination (Appendix 2) is performed on 1d pre-dose(0h), 4h, 12h post-dose, 3d 0h, 5d 0h, 7d pre-dose(0h), 4h, 12h post-dose, 10d 0h, and 13d 0h.

Scheduled time point	Time window
1d 0h, 3d 0h, 5d 0h, 7d 0h, 10d 0h	-60 min
1d 4, 12h, 7d 4, 12h	±30 min

7) Blood samplings and urine collections for PK analysis

Blood samplings for PK analysis are performed on 1d (pre-dose), 0.33, 0.75, 1, 2, 3, 4, 6, 8, 12h post-dose, 2d 0h, 3d 0h, 4d 0h, 5d 0h, 6d 0h, 7d 0h (pre-dose), 0.33, 0.75, 1, 2, 3, 4, 6, 8, 12h post-dose, 8d 0h, 9d 0h,

10d 0h, 11d 0h, 12d 0h, 13d 0h, urine collections for PK analysis are performed on 1d 0h (pre-dose), 0 ~ 12h, 12 ~ 24h (2d 0h), 7d 0h (pre-dose), 0 ~ 12h, 12 ~ 24h (8d 0h), 24 ~ 48h (9d 0h), 48 ~ 72 h (10d 0h) post-dose.

Scheduled time point for urine collections	Time window
1d 0h, 7d 0h	-60 min
1d 0~12h, 12~24h (2d 0h),<br>7d 0~12h, 12~24h (8d 0h), 24~48h (9d 0h), 48~72h (10d 0h)	±30 min

8) Blood samplings for exploratory PD analysis

Blood samplings for exploratory PD analysis are performed on 1d/7d 0h (pre-dose), 2, 24h post-dose.

Scheduled time point	Time window
1d 0h, 7d 0h	-60 min
1d 2h, 7d 2h	±10 min
1d 24h, 7d 24h	-30 min

10.7.3. Post-Study Visit

Subjects who have completed all clinical study visit the clinical trials center and conduct the following observations and tests. Those who drop out after administering the investigational drug visit the clinical trials center within 7 days of the dropout (or the first recognition of dropout) and conduct a planned safety test at the time of post-study visit. However, if the subject refuses to visit, if possible, concomitant medications and lists related to adverse events should be checked by phone.

1) Physical examination

Physical examination includes examination of general condition, nutritional status, skin/mucous membranes, eyes (except for visual loss), otolaryngology, thyroid, lung/respiratory system, heart/circulatory system, abdomen, kidney/urogenital system, nerve/psychiatric system, spine/limb/tumors, peripheral circulation, lymphatic system, and other organs.

2) Vital sign

Vital signs include systolic blood pressure, diastolic blood pressure, pulse rate, and body temperature (tympanic membrane). Blood pressure and pulse rate are measured after maintaining a seated position for more than 3 minutes without sudden changes in position.

3) 12-lead ECG

In addition to the basic recording, the ventricular rate (beats/min), PR interval (msec), QRS (msec), QT/QTcB (msec) at which automatic analysis & recording is output are recorded.

4) Pregnancy test

For all women of childbearing potential except surgical infertility, urine hCG test is performed.

5) Clinical laboratory test

The same hematology tests, blood chemistry tests, blood coagulation tests, and urine tests are performed

as in screening.

**Hematology test**	WBC with differential count (segmented neutrophil, eosinophil, basophil, lymphocyte, monocyte), RBC, hemoglobin, hematocrit, platelet
**Blood chemistry test**	AST, ALT, ALP, γ-GTP, LDH, CPK, glucose, total cholesterol, triglyceride, creatinine, BUN, uric acid, total bilirubin, total protein, albumin, sodium, potassium, calcium, chloride, phosphorus, eGFR (measured using the CKD-EPI formula)
**Coagulation test**	PT (INR), aPTT
**Urine test**	dipstick (color, pH, specific gravity, albumin, bilirubin, glucose, urobilinogen, ketone, nitrite, occult blood, WBC) microscopy (RBC, WBC, squamous cell, others)

6) Neurological examination

A full neurological examination (Appendix 1) is performed.

7) Concomitant medication monitoring

8) Adverse events monitoring

10.8. Subject Cautions/Restrictions

1) Dietary Compliance

① Drinking water intake

- In the case of a single dose study, drinking water is prohibited from 1 hour before administration of the investigational drug to 4 hours after administration of the investigational drug. In case of multiple dose study, drinking water is prohibited from 1 hour before administration of the investigational drug to 4 hours after administration of the investigational drug on 1d and 7d(For the elderly, drinking water is prohibited for up to 2 hours and food intake is prohibited for up to 4 hours after administration). In 2d ~ 6d, drinking water is prohibited from 1 hour before administration of the investigational drug to 2 hours after administration of the investigational drug. (However, drinking water consumed when administering the investigational drug is excluded.)
- The Investigational drug can be administered orally with 150 mL of water, and additional drinking water required for oral administration can be taken if necessary.
- During the period of admission to the clinical trial institution, free intake of prescribed drinking water is permitted, but all other beverages are prohibited.

② Meal

- During the period of admission to the clinical trial institution, only meals provided by the institution are allowed.
- Subjects should fast for at least 10 hours prior to administration of the investigational drug.
- Grapefruit, grapefruit juice, foods containing grapefruit, foods containing caffeine/caffeine (coffee, tea (black tea, green tea, etc.), carbonated drinks, coffee milk, nourishing tonic drinks, etc.) should be taken from 3 days prior to the first administration of the clinical trial drug. Prohibited during the period until the end of the clinical trial.

③ Drinking

- Abstain from alcohol from 3 days before the first administration of the investigational drug to the end of the clinical trial

2) Activity restrictions

① Period of stay at the clinical trial institution

- As stated in the principle, subjects are admitted to the clinical trial institution one day before administration of the investigational drug, and discharged after the completion of each scheduled clinical study.

② Smoking

- Smoking is prohibited for 3 months before the first administration of the investigational drug, and until the time of post-study visit (PSV).

③ Posture

- Subjects must sit at 45 degrees or higher or stand upright for up to 2 hours after administration. However, if the investigator determines that it is medically necessary, such as dizziness when standing up, the supine position is permitted.
- Abrupt changes in posture should be avoided on other days of the study period.

④ Activity

- Subjects should avoid irregular behaviors such as excessive exercise, excessive intake of food or beverages, and prolonged standing that may affect safety endpoints during the entire study period.

10.9. Blood Samplings/Urine collection, Sample Storage and Analysis Methods for PK, PD Analysis

10.9.1. Blood Samplings/Urine collection, Sample Storage and Analysis Methods for PK Analysis

1) Blood sampling method and pretreatment and storage method of separated plasma

For pharmacokinetic analysis, the investigator or participating research team collects blood from the subject at a scheduled time point. Collect more than 8 mL of blood in a K2-EDTA tube, store in an icebox. And within 30 minutes, centrifuge at 3,000 rpm for 10 minutes at 4°C. After that, only plasma from the supernatant is separated, and approximately 1 mL each is dispensed into 4 polypropylene tubes (2 Primary, 2 Back-up) and stored frozen at an average of -70℃ until delivered to a sample analysis institution. Thereafter, the sample for analysis (primary) is transported to the analysis institution, and the sample for storage (back-up) is stored at the testing institution. Specimens for storage will not be used for any purpose other than this test, and will be discarded in accordance with the relevant laws and regulations and the SOP of the concentration analysis institution.

When transporting to a concentration analysis institution, keep the temperature below -70℃ and keep the temperature record result and sample management record during transport. The sample transport address and contact information are separately provided by the sponsor to the clinical trial institution prior to the initiation of the clinical study.

During hospitalization, a catheter can be placed in the brachial vein to minimize the patient's pain due to frequent blood samplings. In the case of blood sampling using a catheter, use a sterile disposable device to prevent infection, collect blood for pharmacokinetic analysis, and inject 1.0 mL of saline after blood sampling to prevent blood clotting. When collecting blood after the first blood collection, collect the blood after withdrawing about 1.0 mL to remove the previously injected saline. After the drug concentration analysis is completed, the remaining samples will not be used for any purpose other than this study, and will be discarded in accordance with the relevant laws and regulations and the SOP of the concentration analysis institution.

The maximum allowable time windows that are not considered protocol violations in blood samplings for pharmacokinetic analysis are as follows.

① Single ascending dose study (30 mg, 60 mg, 240 mg, 480 mg, 960 mg)

Scheduled time point	Time window
Pre-dose (1d 0h)	Within -60 minutes before administration
1d 0.33, 0.75, 1h post-dose	±5 minutes of scheduled blood sampling time point
1d 2, 3, 4, 6, 8, 12h post-dose	±10 minutes of scheduled blood sampling time point
1d 24, 48, 72h post-dose	±30 minutes of scheduled blood sampling time point
1d 96, 120, 144h post-dose	±120 minutes of scheduled blood sampling time point

② Single dose study (120 mg, Food effect study)

Scheduled time point	Time window
Pre-dose (phase 1/2 1d/ 0h)	Within -60 minutes before administration
phase 1/2 1d 0.33, 0.75, 1h post-dose	±5 minutes of scheduled blood sampling time point
phase 1/2 1d 2, 3, 4, 6, 8, 12h post-dose	±10 minutes of scheduled blood sampling time point
phase 1/2 1d 24, 48, 72h post-dose	±30 minutes of scheduled blood sampling time point
phase 1/2 1d 96, 120, 144h post-dose	±120 minutes of scheduled blood sampling time point

③ Multiple ascending dose study (60mg, 120 mg, 240 mg, 480 mg, 120 mg(elderly))

Scheduled time point	Time window
Pre-dose (1d/7d 0h)	Within -60 minutes before administration
1d/7d 0.33, 0.75, 1h post-dose	±5 minutes of scheduled blood sampling time point
1d/7d 2, 3, 4, 6, 8, 12h post-dose	±10 minutes of scheduled blood sampling time point
2d 0h, 3d 0h, 4d 0h, 5d 0h, 6d 0h post-dose	Within -30 minutes before administration
8d 0h, 9d 0h, 10d 0h post-dose	±30 minutes of scheduled blood sampling time point
11d 0h, 12d 0h, 13d 0h post-dose	±120 minutes of scheduled blood sampling time point

2) Urine collection method and pretreatment and storage method of collected urine

Just before drug administration, collect 6 mL of baseline spot-urine, and then collect urine continuously. At the end of each urine collection period, all subjects must empty their bladders to collect urine, and the urine collected during each collection period must be immediately stored in a refrigerator maintained at about 4°C until the end of the collection period. At the end of each urine collection period, the collected urine is weighed using a scale, etc. (assuming that 1 mL of urine is 1 g) and recorded in the case report form. After measuring the volume, shake it appropriately to make it homogeneous, put about 12 mL each in a polypropylene tube, and transfer 1.5 mL of this to each of the 4 eppendorf tubes. Samples are stored in a freezer at -70 °C or lower until analysis.

3) Analysis method

Analysis of KDS2010 in plasma and urine is applied to sample analysis after verification of the method is completed using LC-MS/MS. If necessary, the pharmacokinetics of the KDS2010 metabolite can be analyzed exploratory using plasma and urine samples.

10.9.2. Blood Samplings, Sample Storage and Analysis Methods for Exploratory PD Analysis

About 20 mL of blood is collected in K2-EDTA in the same way as blood sampling for pharmacokinetic analysis, and pretreatment and storage are carried out according to a separate manual.

11. Criteria for Suspension and Early Termination of Clinical Study

11.1. Suspension of Clinical Study

If the Principal Investigator decides that it is not wise to continue the clinical study in light of the results observed during the clinical study, part or all of the study may be discontinued in consultation with the sponsor.

1) The principal investigator may suspend part or all of the clinical study without consultation with the sponsor if there is a serious reason that the subject cannot continue the clinical study and it is judged that it is urgent.
2) The sponsor may suspend part or all of the clinical study for safety or management reasons.
3) If the clinical study is early terminated or suspended, the principal investigator must immediately notify the subject of this fact and ensure that appropriate measures and follow-up can be made.
4) The eCRF and clinical study progress status and results for the subject who have progressed until the time of suspension are organized and delivered to the sponsor, and all study-related data (complete, incomplete or undescribed eCRF and investigational drug, etc.) must be returned to the sponsor.
5) If the clinical study is suspended, it must be reported to the IRB and the Ministry of Food and Drug Safety.

If the principal investigator determines that unexpected problems, serious protocol violations, or serious adverse events occurring in the course of the clinical study are not enough to qualify for the suspension of the clinical study, the clinical study will be continued in accordance with the results of consultation with the sponsor and the IRB's review. If necessary, the clinical study can proceed by amending the clinical study protocol.

11.2. Early Termination

The sponsor may terminate the study at the clinical trial institution early. The reasons for termination of clinical study by sponsor are as follows.

1) When the clinical trial institution fails to register the target number of subjects

2) When safety information is generated that can affect continuation of clinical trials
3) In case the degree of violation of GCP, protocol, or contract by a clinical trial center or investigator causes problems in the continuation of clinical study
4) Other administrative reasons that may have an important impact on the continuation of clinical study

In the event that the sponsor early terminates or temporary suspends the clinical study, the investigator should immediately inform IRB of this fact and submit a detailed statement of reasons for early termination or temporary suspension. If the clinical study is early terminated or temporary suspended, the investigator should immediately notify the subject of this fact and ensure that appropriate measures and follow-up can be made. The principal investigator should organize the case report form and clinical study progress status and results for the subject who have progressed until the time of suspension and deliver to the sponsor. The investigational drugs and all study-related data should be returned to the sponsor.

11.3. Protocol Deviations

In the event of unexpected serious protocol deviation, the principal investigator or investigator should inform the sponsor. In case of non-compliance with the clinical study protocol, the date of occurrence, the fact of the occurrence, the reason for the occurrence, measures, and a plan to prevent recurrence should be written in documents so that the monitoring staff can confirm it.
If the sponsor or monitor staff becomes aware of an unexpected problem or deviation from the clinical study protocol, it should be immediately notified to the principal investigator or investigator.
The principal investigator or sponsor should report the deviation to IRB according to the reporting procedures prescribed by the institution and some or all of the clinical study can be terminated according to IRB decision.

12. Safety/Tolerability and PK, PD Assessments

12.1. Safety/Tolerability Assessments

12.1.1. Safety/Tolerability Endpoint

- Adverse events (AEs) including subjective/objective symptoms and signs etc.,
- Vital signs, physical examinations, clinical laboratory tests, 12-lead ECGs
- Neurologic examination (Full or brief)

12.1.2. Safety/Tolerability Analysis

All AEs are classified using the MedDRA (Medical Dictionary for Regulatory Activities). Summarize the number of subjects (occurrence rate) and the number of occurrences of adverse events/adverse drug reactions and serious adverse events/adverse drug reactions by dose group and treatment group. Vital signs, 12-lead ECGs, and clinical laboratory tests are presented as descriptive statistics (mean, standard deviation, median, minimum, maximum) for the values measured at each time point and the amount of change from the baseline by dose group for continuous variables. In addition, for test results classified as normal, not clinically significant abnormal (NCS), and clinically significant abnormal (CS), a shift table after administration compared to the baseline is presented. A separate list is presented when there is a change from a normal or a not clinically significant abnormal (NCS) to a clinically significant abnormal (CS). Neurological examination summarizes the frequency of clinically significant abnormalities at each time point, and descriptive statistics according to appropriate classification such as dose group.

12.2. Pharmacokinetic Aseessments

12.2.1. Pharmacokinetic Endpoints

- Single ascending dose study
  - Primary endpoints: AUC_{last}, AUC_{inf}, C_{max}, T_{max}, $t_{1/2}$, CL/F, V_z/F, fe, CL_R for KDS2010
  - Secondary endpoints: AUC_{last}, AUC_{inf}, C_{max}, T_{max}, $t_{1/2}$, metabolic ratio for KDS8170(main metabolite of

KDS2010)

- Multiple ascending dose study
  - Primary endpoints: AUC_{tau}, $C_{max,ss}$, $C_{min,ss}$, $C_{av,ss}$, $T_{max,ss}$, $t_{1/2}$, PTF, CL_{ss}/F, $V_{d,ss}$/F, fe, CL_R, accumulation ratio (Ra) for KDS2010
  - Secondary endpoints: AUC_{tau}, $C_{max,ss}$, $C_{min,ss}$, $C_{av,ss}$, $T_{max,ss}$, $t_{1/2}$, PTF, accumulation ratio (Ra), metabolic ratio for KDS8170(main metabolite of KDS2010)

12.2.2. Pharmacokinetic Analysis

In pharmacokinetic analysis, blood sampling/urine collection time is analyzed using the actual blood sampling/urine collection time for each subject. If the measured concentration is below the lower limit of quantification(LLOQ), or no actual blood sampling has been performed (not applicable), or the sample is missing (missing data), it is written as "LLOQ", "NA", or "MD" in the blood drug concentration data, respectively. If the blood concentration is less than the lower limit of quantification before T_{max}, it is regarded as 0, and if the blood concentration observed near the terminal after T_{max} is less than the lower limit of quantification, it is regarded as a missing data. The obtained data are used to calculate pharmacokinetic parameters by a noncompartmental method using WinNonlin® (Certara, CA, USA).

12.3. Pharmacodynamic Assessments

12.3.1. Pharmacodynamic Endpoints

- MAO-B activity

12.3.2. Pharmacodynamic Analysis

Analyze the change and rate of change of MAO-B activity compared to the baseline.

13. Reporting of Adverse Events

13.1. Definition of Safety Terms

13.1.1. Adverse Event (AE)

An adverse event (AE) refers to all harmful and unintended signs(including an abnormal laboratory finding), symptom or disease that occur in subjects who have administered the investigational drug, and it does not necessarily have to have a causal relationship with the investigational drug.

13.1.2. Adverse Drug Reaction (ADR)

An adverse drug reaction (ADR) refers to all noxious and unintended responses to the investigational drug related to any dose. The phrase “responses to the investigational drug” means that a causal relationship between investigational drug and an adverse event is at least a reasonable possibility, i.e., the relationship cannot be ruled out.

13.1.3. Serious Adverse Event/Adverse Drug Reaction (AE/ADR)

Any AE/ADR occurring at any dose of the investigational drug that results in any of the following outcomes:

1) Results in death or life-threatening

2) Requires inpatient hospitalization or prolongation of existing hospitalization
3) Results in persistent or significant disability/incapacity
4) Results in congenital anomaly/birth defect
5) In addition to cases 1) to 4), cases where drug dependence or abuse occurs or other medically important circumstances such as occurrence of blood diseases

If a situation that is medically considered to have a significant impact on the subject's safety and health condition other than those listed above occurs, it is decided whether or not to be regarded as a serious adverse event according to the medical judgment of the investigator and related experts. And take appropriate action accordingly. However, the following subjects are not considered SAEs.

- Hospitalization for pre-planned treatment/surgical procedures prior to participation in the clinical study, or hospitalization selected by the subject for pre-existing concomitant diseases that did not worsen during the study period, and extension of hospitalization period accordingly (Hospitalization for a rehabilitation institution, nursing institution, nursing home, or temporary care that continued before administration of the investigational drug, and hospitalization for laboratory abnormalities, when the subject lives far from the hospital or there is no caregiver at home, etc.)
- Discharge within 24 hours of hospitalization (Emergency room visits that do not result in hospitalization)

13.1.4. Suspected Unexpected Serious Adverse Reaction (SUSAR)

Suspected Unexpected Serious Adverse Reaction (SUSAR) is suspected of being drug-related, unexpected, and serious adverse drug reaction.

13.2. Recording and Reporting of Adverse Events

1) Adverse events must be specified in source document and case report form. In source document, the symptoms or signs are freely indicated as subject expression or opinion of principal investigator or clinical study doctor. However, case report form based on source document is recorded using MedDRA (version 24.0 or the latest available version) as much as possible and record as LLT (Lowest Level Term), and encode according to SOC and PT during analysis.
2) The symptoms and signs from screening to drug administrationare recorded in "medical history" of CRF.
3) In the case report form, the symptoms and signs, the date and time of onset (if possible), the action taken, the outcome, the severity of the adverse events and the causal relationship with the investigational drug should be described. The clinical trial coordinator may write the case report form for adverse events, but the final review and confirmation must be done by the principal investigator. In addition, the content, start, termination, total duration, and action taken of the adverse events must be written in a case report form based on the source document. The degree of occurrence, result, causal relationship, and reporting method can be directly recorded in the case report form by the principal investigator or the clinical study doctor, and in this case, the content of the case report form becomes the source data.
4) In case the symptoms and signs of an adverse event persist, that is, if the outcome of the adverse event is 'continuation of adverse event', even if observation of the subject is terminated at the discretion of the principal investigator or the clinical study doctor, the records of the voluntary reports of subject should be kept in the source document. If necessary, the case report form may be rewritten including this.
5) Information to be recorded in the case report form

   ① Symptom and sign

   ② Date and time of onset (if available)

   ③ Date and time of end (if available)

   ④ Outcome

   ⑤ Severity

⑥ Causal relationship with the investigational drug
⑦ Action taken
⑧ SAE or not
⑨ Progress

13.3. Reporting of Serious Adverse Events

In the case of a serious adverse event, the investigator sends a report of the serious adverse events by e-mail or fax to the person in charge of Neurobiogen Clinical Development Headquarters within 24 hours of recognizing it, regardless of whether there is a causal relationship between the adverse event and the investigational drug.

**Neurobiogen Co., Ltd.**	**PV manager**	**Fax: 02-572-9011**	**E-mail: ekpark@iprotox.com**
**C&R Research Inc.**	**PV manager**	**Fax: 02-6251-1504**	**E-mail: cnr_pv@cnrres.com**

Whenever possible, the initial report should include all items on the serious adverse event form, and the completed form should be sent to the sponsor. In addition to the serious adverse event form, the investigator should record the adverse event page of the case report form. If necessary, a follow-up report including all new information on the serious adverse event should be written in a serious adverse event form and sent to the sponsor, and should also be reported to the IRB according to the reporting procedure prescribed by the institution.
After receiving the initial report, the sponsor and the IRB should review the information and, if necessary, contact the investigator to obtain more detailed information. After reviewing the information of adverse event, the causal relationship with the investigational drug should be investigated.
If a suspected unexpected serious adverse reaction occurs, the sponsor should report it to the Minister of Food and Drug Safety within the following deadlines.

- Resulted in death or is life-threatening: Within 7 days from the date the sponsor was notified or became aware of the fact. In this case, the detailed information on the according adverse event must be submitted additionally within 8 days from the date of initial report.
- For all other suspected unexpected serious adverse reactions : Report within 15 days from the date the sponsor receives or becomes aware of the fact.

Although it does not correspond to a serious adverse event, the investigator is obliged to report in a special case (pregnancy). The sponsor is responsible for follow-up of pregnancy outcomes reported during the clinical study. If pregnancy occurs during the clinical study period (up to 30 days after the end of the clinical study) in the spouse (or partner) of the subject who administered the investigational drug, report it to the IRB and the sponsor within 24 hours of recognizing the pregnancy, and pregnancy processes and outcomes should be tracked and documented.

13.4. Adverse Events and Causal Relationship Evaluation with Investigational Drugs

13.4.1. Severity

The severity of adverse events is classified according to NCI-CTCAE version 5.0 or higher, and adverse events not classified as NCI-CTCAE are evaluated according to the following five steps.

- Grade 1: Mild - Awareness of signs or symptoms, but easily tolerated
- Grade 2: Moderate - Discomfort severe enough to cause interference with usual activities
- Grade 3: Severe - Incapacitating and causing inability to perform normal activities
- Grade 4: Life-threatening consequences
- Grade 5: Death

13.4.2. Actions Taken in relation to Adverse Events

1) Action taken with Investigational drug

- Drug-withdrawn
- Dose reduced
- Dose not changed
- Not applicable

2) Others

- Co-medication given
- Non-drug therapy given
- Not Done
- Unknown
- Not Applicable

13.4.3. Outcome of Adverse Events

- Recovered
- Recovering
- Not recovered
- Recovered with sequelae
- Fatal
- Unknown

13.4.4. Progress of Adverse Events

- Once
- Intermittent
- Continuous

13.4.5. Causal Relationship with Investigational Drugs

Causal relationships with the investigational drugs are classified into the following six categories. And if necessary, the views of the principal investigator or investigator are recorded. Among the causal relationships, if it is evaluated "definitely related", "probably related", "possibly related", and "unknown" except for "unlikely related" and "definitely not related", it is evaluated as an adverse drug reaction that cannot exclude a causal relationship.

① Definitely related

- When there is evidence of drug administration and the temporal sequence of adverse events is appropriate
- The adverse event is most likely explained by the administration of the drug rather than any other reason.
- If the result is positive when re-administered (only if possible)

- When the adverse event is consistent with known information about the drug or drug in the same class
- If there is a clinically reasonable response to discontinuation of administration

② Probably related

- When there is evidence of drug administration and the temporal sequence of adverse events is appropriate
- The adverse event is most likely explained by the administration of the drug rather than any other reason.
- If there is a clinically reasonable response to discontinuation of administration
- If there is no re-administration information

③ Possibly related

- When there is evidence of drug administration and the temporal sequence of adverse events is appropriate
- When the adverse event is judged to be attributable to the drug administration to the same extent as other probable causes.
- In case of insufficient or unclear information regarding discontinuation of administration

④ Unlikely related

- When there is evidence of drug administration and the temporal sequence of the onset of adverse events is not appropriate
- In the case of a temporary case that is unlikely to have a causal relationship with the administration of the drug
- When there is a more probable cause for the adverse event

⑤ Definitely not related, None

- When the clinical study subject has not been administered the drug
- When the temporal sequence between drug administration and the onset of the adverse event is not appropriate
- When there is another apparent cause for the adverse event

⑥ Unknown, Unassessable

- When there is insufficient evidence to determine the relationship
- In case the quality of the source data is low or there is inconsistency between the data
- In case the information is insufficient or conflicting and cannot be determined, and it cannot be supplemented or verified.

13.5. Follow up of Adverse Events

Investigators should follow-up the subjects with adverse events until symptoms subside and abnormal clinical laboratory test results return to baseline, or until a satisfactory explanation for the observed changes is provided. If the subject does not make follow-up visits, check their health status in writing or by phone, etc., and record the reason why the follow-up visit is impossible as a source document.
When there is a change in status of the subject who has been previously reported a serious adverse event (eg, discharge after hospitalization, a worsening of the condition) or additional information is collected, the investigator delivers a serious adverse event report via fax or e-mail to the person in charge of Neurobiogen Clinical Development Headquarters within 24 hours of recognizing this.

14. Analytical and Statistical Considerations

14.1. Analysis Populations

1) Demographic information analysis group
Demographic information is analyzed for all subjects (Intention-To-Treat) assigned a randomization number.

2) Safety/tolerability evaluation population
All subjects who have been administered investigational drugs more than once. Analyze according to the type of dose group and treatment group.

3) Pharmacokinetic evaluation population
After completing the clinical study, the subjects who can evaluate the pharmacokinetic results are analyzed. Pharmacokinetic evaluation basically targets subjects with evaluable drug concentrations. If a subject drops out after receiving drug administration, the results obtained from the subject until the time of discontinuation or withdrawal may also be reviewed.

4) Pharmacodynamic evaluation population
After completing the clinical study, the subjects who can evaluate the pharmacodynamic results are analyzed.

14.2. Statistical Analysis Methods

1) Demographic information
For demographic information such as age, height, and weight, descriptive statistics (mean, standard deviation, median, minimum, maximum, etc.) or category frequency and ratio are presented according to the characteristics of the data.

2) Safety and Tolerability Analysis
For safety/tolerability analysis, the abnormal findings observed in safety evaluation lists such as the occurrence pattern of adverse events, vital signs and clinical laboratory results are described, and if necessary, each list is compared according to appropriate classification such as dosing group. Medical history and AEs are coded using the MedDRA (ver. 24.0 or later).

3) Pharmacokinetic Analysis
After calculating pharmacokinetic parameters for each subject, descriptive statistics are summarized by appropriate classification such as dosing group.

To evaluate the pharmacokinetic linearity according to the dose increase, the relationship between C_{max}, AUC(AUC_{inf}, AUC_{last}) and the administered dose is evaluated through regression analysis, and C_{max}, AUC corrected for the administered dose are compared between dosing groups through a parametric or non-parametric statistical test. In addition, parameters such as T_{max}, $t_{1/2}$, CL/F can be compared between dosing groups through an appropriate method.

Food effect evaluation is performed by calculating the geometric mean ratio (GMR) of the pharmacokinetic parameters (C_{max}, AUC) after a high-fat meal to the fasting and its 90% confidence interval (CI).

4) Exploratory Pharmacodynamic Analysis
After calculating exploratory pharmacodynamic parameters for each subject, descriptive statistics are summarized by appropriate classification such as dosing group.

14.3. Interim Analysis

No interim analysis is scheduled in this clinical study.

15. Ethics of Research

This clinical trial will be conducted ethically and scientifically in accordance with the KGCP and all relevant regulations. In addition, this clinical study will be conducted in accordance with the Declaration of Helsinki, respecting human dignity and rights and not causing any disadvantage to the subject.

16. Data Processing and Quality Assurance

16.1. Source Documents

Source documents mean all documents, data, and records such as medical records, electrocardiograms, clinical laboratory test results, and drug dispensing records. Data collected during the study period should be recorded in appropriate source data. Data recorded in the screening/registration log should include the subject randomization number, screening date, and reason for dropping out (in case of dropout), and all screened subjects should be recorded in this log. Access to source data is permitted during clinical study monitoring, inspection, IRB review, and fact-finding by the permitting authority.

16.2. Case Report Forms

If eCRF is not recorded until end of clinical study, the appropriate reason should be recorded. All eCRF have remote data collection based on source documents. Sponsor should monitor eCRF periodically and the principal investigator should review the completion and accuracy of the eCRF before writing electronic signature. Data will be frozen after monitoring and completion of principal investigator signature. Thereafter, data validation is performed using an appropriate computer program (SAS, etc.) to verify whether there are logical errors, missing values, or outliers in the eCRF. For all errors found, it is judged whether it is a query, and a query is issued to the clinical trial institution, and the eCRF is revised after the institution confirms the original data. Source documents and eCRF should be kept after verified all data for IRB or relevant government agencies. In addition, investigator should present normal range or references in eCRF to identify and verify the computerized record before clinical study initiation.

16.3. Data Entry Process

eCRF will be used in this study. All information recoded in the eCRF is based on subject's source documents and completed the eCRF of all subjects who signed the consent form. The data of eCRF is input through the database system developed using CRScube, and the data input process is performed according to the SOP of the clinical trial institution. Since complete pharmacokinetic data are created after eCRF input is completed, pharmacokinetic data are excluded from the database system entry. After reviewing the completeness and accuracy of the eCRF, the principal investigator digitally signs it.

16.4. Storage of Study Documents

In accordance with the KGCP, clinical study-related documents have to be retained for 3 years from the date of product approval or 3 years from the date of completion of the study. The retention period may be extended if the Minister of Food and Drug Safety instructs to do so or if the sponsor determines that it is necessary.

16.5. Data Security

All documents, including source documents and eCRF obtained during the clinical study, have to be retained securely, and it should be stored in a locked laboratory (such as a nurse station in a clinical trial center or an investigator's office). The investigator shall not disclose any of the information without consent from the sponsor. Since anonymity of subjects has to be guaranteed, all documents shall use subject numbers and initials instead of names of subjects. Any documents that may identify subjects shall be retained securely by the investigator.
If clinical information collected for the purpose of this study is provided to a third party or registered in an open database, information from those who have agreed will be provided, and will be provided as anonymously.

16.6. Confidentiality and Publication of Study Results

Since all information obtained from the clinical study is the exclusive intellectual property of the sponsor, the investigator or anyone else involved must strictly maintain confidentiality. Investigators may publish information or data related to this study after consultation with the sponsor.

17. Other Considerations Required for Conduct of Safe and Scientific Clinical Study

17.1. Institutional Review Board (IRB)

Before starting the clinical study, the investigator must be reviewed by IRB about the investigator's brochure, clinical trial protocol, subject informed consent form, method of recruiting subjects (including the Internet homepage (http://ctcr.snuh.org), etc.), and other various information provided to the subject in the form of documents. The IRB communicates the results of its review on study implementation to the sponsor and investigator in writing before the initiation of the clinical study. The principal investigator should conduct the clinical study after obtaining the IRB's approval for the clinical trial protocol and amendments. In addition, events that may affect patient safety or the continuous performance of the clinical study, including serious adverse events, in particular safety-related changes, must be reported to the IRB. In accordance with the relevant IRB standards, if necessary, a report on the progress should be submitted to the IRB, and the IRB should be notified at the end of the clinical study.

17.2. Clinical Study Institution

The clinical study institution shall be fully equipped with facilities and specialized personnel required to conduct the clinical study and be fully prepared to conduct this clinical study properly.

17.3. Informed Consent Procedures

The investigator explains to the subject (or his/her representative) participating in the clinical study using pure Korean (native language) so that they can easily understand the nature, scope, and expected result of the study in advance. However, in the case of Caucasians, explanations are made using language that the subject can easily understand. The investigator confirms that the subject clearly understands that he or she can decide to participate in the clinical study at his or her own will, and that he or she can refuse to participate in the clinical study or stop it at any time during the study period without any loss or loss of benefit. After hearing the explanation about the clinical study, the subject has enough time to consider participating in this clinical study, and can sufficiently discuss with family or acquaintances. In addition, if the subject does not understand the clinical study, he or she can ask the investigator for explanation at any time and should be able to receive a satisfactory answer. The investigator must obtain the subject's consent in writing, and the subject's consent form must be signed and dated by the investigator and subject in his/her own handwriting. Among the elderly subjects, if necessary, consent capacity evaluation (e.g., the mini-mental state examination (MMSE) or the Korean Version CSS (Capacity-to-Consent Screen), etc.) can be performed by the researcher. If it is determined that the consent capacity is incomplete, written consent may be obtained from the agent. The original signed consent form is kept by the investigator, and a copy of the signed consent form and an information sheet must be given to the subject (or his/her representative). Investigator should not conduct any tests for the purpose of clinical study before obtaining consent from the subjects. By signing the consent form, the subject consents to the collection and use of the subject's personal information in relation to the participation in the clinical study. Subject's personal information is used for the purpose of identifying the subject's identity and linking it with the clinical information collected during the clinical study process. And collected personal information is not directly used as clinical study data by encoding it so that the identity of the individual cannot be identified. The scope of personal information collected in relation with clinical study includes personally identifiable information (name, contact information, etc.), demographic information, medical records (past medical history, treatment history, etc.) and test results performed in relation with clinical study. All collected data is handled in accordance with laws, rules and regulations on the protection of personal information.

17.4. Actions to Secure Subject Safety

The investigator should thoroughly confirm that the subjects are healthy enough to participate in clinical trials by checking the health status of each subject before enrollment. In addition, the investigator will fully understand

protocol and investigational drugs, and conduct clinical study according to the protocol. Investigators must be best to ensure the safety of the subject. If an adverse event occurs that requires treatment, the subject should be given a diagnosis and appropriate treatment by a medical doctor. In addition, if necessary, observation should be made until the relevant adverse event disappears or follow-up becomes impossible. During the clinical study period, if a subject wants to receive treatment due to an adverse event, or if the investigator determines that medical treatment is necessary, he/she must immediately visit the clinical trial institution and undergo relevant examinations. If the investigator evaluates the adverse event and test results and determines that the study should be stopped, or if the subject wants to stop the study, the investigator will promptly proceed with the procedure following the end of the clinical study and actively provide treatment to the subject to alleviate the symptoms.

17.4.1. Subjects Confidentiality

All subject's names should be kept confidential. The signed consent form is kept by the principal investigator and the investigators delegated by the principal investigator. The subject's real name and medical record number are kept in a separate file under the responsibility of the principal investigator, and information about the subject's identity is managed so that it is not known to anyone except those who are permitted to directly view the data.

17.4.2. Storage and Disposal of Human Derived Materials

Human derived materials such as samples for drug concentration analysis are analyzed through an external analysis institution, and some may be stored for re-analysis. At this time, the subject's personal information will not be recorded in the storage tube, and after the relevant period has elapsed, it will be disposed of in an appropriate manner in accordance with the relevant laws and internal regulations. The subject may request the investigator to discard the samples in storage at any time.

17.5. Data Quality Control and Quality Assurance

To protect the rights and welfare of subjects, to ensure that reported clinical study data are accurate, complete and verifiable against source documents, and to ensure that clinical study is conducted in compliance with approved protocols, KGCP and related regulations, the sponsor may conduct periodical monitoring. Monitoring of clinical study will be conducted through regular visits and contacts to the clinical trial institution by the sponsor (or a person authorized by the sponsor) or the monitor. The schedule of these visits should be distributed through appropriate consultation between the investigator and the monitor.
The monitor will visit the clinical study institution and check the completeness and clarity of the case report form, contrast with source data, and check the management tasks. And the investigator must cooperate with this. During the monitoring visit, the monitor will review informed consent, recruitment and follow-up of subjects, recording and reporting of serious adverse events, assignment of investigational drugs, patient's compliance to use and dosage of investigational drugs, quantity of investigational products, concomitant medications, and data quality, etc. together with the investigator.
To ensure reliability during the clinical study period, the sponsor may conduct an inspection independent of routine monitoring. Inspection includes checking whether this clinical study is being conducted in accordance with the clinical trial protocol, SOP, and relevant regulations such as KGCP, and reviewing all source data, drug records, medical records, etc. The sponsor (or the person delegated with the sponsor) may request to read the source documents and other basic documents for inspection of the clinical trial institution, and the investigator should allow this and cooperate with this process.
The Ministry of Food and Drug Safety may conduct a fact-finding survey during or after the clinical study. When a fact-finding survey is scheduled, the investigator must immediately notify the sponsor of this fact. The Ministry of Food and Drug Safety may request access to the source documents and other basic documents for the fact-finding survey of the clinical trial institution, and the investigator should allow this and cooperate with this process.

17.6. Protocol Amendments

The contents of this clinical trial protocol cannot be changed/modified without mutual consent after detailed discussion with the sponsor. Amendment in the clinical trial protocol are applied to all subjects after obtaining approval from the Medical Research Ethics Review Board (IRB) and, if necessary, the Ministry of Food and Drug Safety.

18. References

1) FDA Label: ZELAPAR® (selegiline hydrochloride) tablets

2) FDA Label: XADAGO® (safinamide) tablets

3) Estimating the Maximum Safe Starting Dose in Initial Clinical Trials for Therapeutics in Adult Healthy Volunteers, FDA Guidance for Industry, July 2005

4) Food-effect bioavailability and fed bioequivalence studies. FDA Guidance for Industry, Dec 2002.

5) The Validation of a Measurement for Assessing the Capacity of Korean Older Adults to Consent to Research, Korean Journal of Social Welfare, Vol. 61, No. 3, 2009. 8, pp. 55-76

19. Appendices

Appendix 1. Full Neurologic Examination

Appendix 2. Brief Neurologic Examination

Appendix 3. MMSE (Mini-Mental State Exam)

Appendix 4. K-CCS (Korean Version of Capacity-to-Consent Screen)

20. Annex

Annex 1. Informed Consent Form

Annex 2. Indemnity agreement for human participant

Annex 3. List of Investigator and Pharmacist

Appendix 1. Full Neurologic Examination

GENERAL	Normal	Abnormal, NCS	Abnormal, CS	Not Done	If Abnormal, Specify Findings – including laterality, as applicable
1. Level of Consciousness	☐	☐	☐	☐	
2. Mental Status	☐	☐	☐	☐	
3. Visual Fields (II)	☐	☐	☐	☐	
4. Eye Movements (III, IV, VI)	☐	☐	☐	☐	
5. Jaw Movement and Facial Sensation (V)	☐	☐	☐	☐	
6. Facial Motion (VII)	☐	☐	☐	☐	
7. Hearing (VIII)	☐	☐	☐	☐	
8. Swallowing, pharynx, larynx (IX, X)	☐	☐	☐	☐	
9. SCM, trapezius (XI)	☐	☐	☐	☐	
10. Tongue (XII)	☐	☐	☐	☐	
11. Biceps Reflexes	☐	☐	☐	☐	
12. Triceps Reflexes	☐	☐	☐	☐	
13. Patellar Reflexes	☐	☐	☐	☐	
14. Achilles Reflexes	☐	☐	☐	☐	
15. Plantar Reflexes	☐	☐	☐	☐	
16. Gait	☐	☐	☐	☐	
17. Romberg	☐	☐	☐	☐	
18. Nystagmus	☐	☐	☐	☐	
19. Tremor	☐	☐	☐	☐	
20. Finger-Nose	☐	☐	☐	☐	
21. Heel-Shin	☐	☐	☐	☐	
22. Rapid Alternating Movements	☐	☐	☐	☐	
23. Muscle Strength	☐	☐	☐	☐	
24. Pin	☐	☐	☐	☐	
25. Vibration	☐	☐	☐	☐	

Appendix 2. Brief Neurologic Examination

GENERAL	Normal	Abnormal, NCS	Abnormal, CS	Not Done	If Abnormal, Specify Findings – including laterality, as applicable
**1. Level of Consciousness**	☐	☐	☐	☐	
**2. Mental Status**	☐	☐	☐	☐	
**3. Biceps reflexes**	☐	☐	☐	☐	
**4. Knee reflexes**	☐	☐	☐	☐	
**5. General movement**	☐	☐	☐	☐	
**6. Gait**	☐	☐	☐	☐	
**7. Romberg**	☐	☐	☐	☐	
**8. Nystagmus**	☐	☐	☐	☐	
**9. Tremor**	☐	☐	☐	☐	
**10. Finger-Nose**	☐	☐	☐	☐	
**11. Heel-Shin**	☐	☐	☐	☐	
**12. Rapid Alternating Movements**	☐	☐	☐	☐	

Appendix 3. MMSE (Mini-Mental State Exam)

MINI MENTAL STATE EXAMINATION (MMSE)

Name:

DOB:

Hospital Number:

One point for each answer **DATE:**			
**ORIENTATION**<br>Year Season Month Date Time	....../ 5	....../ 5	....../ 5
Country Town District Hospital Ward/Floor	....../ 5	....../ 5	....../ 5
**REGISTRATION**<br>Examiner names three objects (e.g. apple, table, penny) and asks the patient to repeat (1 point for each correct. THEN the patient learns the 3 names repeating until correct).	....../ 3	....../ 3	....../ 3
**ATTENTION AND CALCULATION**<br>Subtract 7 from 100, then repeat from result. Continue five times: 100, 93, 86, 79, 72, 65 (Alternative: spell "WORLD" backwards: DLROW).	....../ 5	....../ 5	....../ 5
**RECALL**<br>Ask for the names of the three objects learned earlier.	....../ 3	....../ 3	....../ 3
**LANGUAGE**<br>Name two objects (e.g. pen, watch).	....../ 2	....../ 2	....../ 2
Repeat "No ifs, ands, or buts".	....../ 1	....../ 1	....../ 1
Give a three-stage command. Score 1 for each stage. (e.g. "Place index finger of right hand on your nose and then on your left ear").	....../ 3	....../ 3	....../ 3
Ask the patient to read and obey a written command on a piece of paper. The written instruction is: "Close your eyes".	....../ 1	....../ 1	....../ 1
Ask the patient to write a sentence. Score 1 if it is sensible and has a subject and a verb.	....../ 1	....../ 1	....../ 1
**COPYING:** Ask the patient to copy a pair of intersecting pentagons	....../ 1	....../ 1	....../ 1
**TOTAL:**	....../ 30	....../ 30	....../ 30

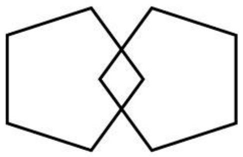

MMSE scoring
24-30: no cognitive impairment
18-23: mild cognitive impairment
0-17: severe cognitive impairment

OME Oxford Medical Education

Appendix 4. K-CCS (Korean Version of Capacity-to-Consent Screen)

Capacity-to-Consent	Questions
Understanding	1. What kind of people can participate in the study?<br>2. What is the purpose of the study?<br>3. What questions will be asked?
Reasoning and Voluntary Choice	4. What do you get from participating in the study?<br>5. Can you refuse the study if you don't want to participate?<br>6. Even though you are not participating in the study, can you take the hospital service?<br>7. Can you stop interviewing whenever you want?<br>8. If you don't want to answer, can you stop responding?
